# Synthesis, Characterization, Fluorescence Properties, and DFT Modeling of Difluoroboron Biindolediketonates

**DOI:** 10.3390/molecules28124688

**Published:** 2023-06-10

**Authors:** Angelo Maspero, Federico Vavassori, Luca Nardo, Guglielmo Vesco, Jenny G. Vitillo, Andrea Penoni

**Affiliations:** Department of Science and High Technology and INSTM, University of Insubria, Via Valleggio 9, 22100 Como, Italy; angelo.maspero@uninsubria.it (A.M.); f.vavassori1@uninsubria.it (F.V.); luca.nardo@uninsubria.it (L.N.); gvesco@uninsubria.it (G.V.)

**Keywords:** indoles, difluoroborn diketonates, fluorescence quantum yield, keto-enolic tautomerism, singlet oxygen photosensitization, TD-DFT

## Abstract

We report a simple and efficient strategy to enhance the fluorescence of biocompatible biindole diketonates (bdks) in the visible spectrum through difluoroboronation (BF_2_bdks complexes). Emission spectroscopy testifies an increase in the fluorescence quantum yields from a few percent to as much as >0.7. This massive increment is essentially independent of substitutions at the indole (-H, -Cl, and -OCH_3_) and corresponds to a significant stabilization of the excited state with respect to non-radiative decay mechanisms: the non-radiative decay rates are reduced by as much as an order of magnitude, from 10^9^ s^−1^ to 10^8^ s^−1^, upon difluoroboronation. The stabilization of the excited state is large enough to enable sizeable ^1^O_2_ photosensitized production. Different time-dependent (TD) density functional theory (DFT) methods were assessed in their ability to model the electronic properties of the compounds, with TD-B3LYP-D3 providing the most accurate excitation energies. The calculations associate the first active optical transition in both the bdks and BF_2_bdks electronic spectra to the *S*_0_ → *S*_1_ transition, corresponding to a shift in the electronic density from the indoles to the oxygens or the O-BF_2_-O unit, respectively.

## 1. Introduction

Difluoroboron β-diketonates (BF_2_bdks) are a class of tetracoordinate organoboron complexes that has been investigated only in the last 16 years, after the first example reported by Fraser and co-workers in 2007 [1]. These complexes have shown excellent photophysical properties, such as broad UV-Vis absorption range, large extinction coefficients, two-photon excited fluorescence, fluorescence in solution up to the NIR region, and, in some cases, solid-state luminescent behavior and room-temperature phosphorescence (RTP).

Another captivating feature of these adducts is the ability to produce singlet oxygen (^1^O_2_), which allows BF_2_bdks to be used as oxygen sensors in tissue visualization or as photosensitizers (PSs) in photodynamic therapy (PTD) [2].

Some modifications in molecular conformation and intermolecular interactions in the solid state influence the luminescence of BF_2_bdks, making them responsive to external stimuli, such as pressure, heat, or mechanical force. Thermochromic or mechanochromic fluorescence is useful in sensoristics for high-end applications [3,4].

Substituents at C1 and C3 of the diketone ligand greatly influence the behavior of the boron compounds, and slight structural modifications on the diketone ligands enable the fine tuning of luminescence properties. Extended π conjugation is known to positively affect fluorescence emission efficiency [5], and intramolecular charge transfer (ICT) is especially favored when the electron-poor dioxadifluoroborine ring portion is coupled with electron-rich aryl scaffolds, along with methoxy or amino groups [6].

Curcuminoid and cyanin-like motifs displaying a β-diketone fragment present a high number of alternate C-C double bonds attached to aromatic structures and have been extensively used in medicinal chemistry, given their biocompatibility, low toxicity, and ability to chelate metals [7]. These peculiarities make β-diketones suitable candidates as ligands in organoboron-based fluorescence chemistry in biomedical applications because an increase in π conjugation shifts absorption and emission spectra to the near-infrared (NIR) region [8,9,10].

Among heterocycles, indole is one of the most important, so much so that it has gained the nickname “The Lord of the Rings” [11,12]. Indole is the backbone of numerous bioactive compounds, both naturally occurring and synthetically produced, such as tryptophan (an essential amino acid), melatonin (a hormone involved in the regulation of the circadian rhythm), vinblastine (naturally present in vinca plants, an essential component in several chemotherapy regimens), and indomethacin, a potent non-steroidal anti-inflammatory drug (NSAID) used as prescription medication [13]. Cirrincione and co-workers firstly reported the use of biindole 1,3-diketones (bdks) as a frame to synthesize nortopsentin analogues, a class of marine alkaloids with anticancer and antibacterial properties [14].

**Scheme 1:**
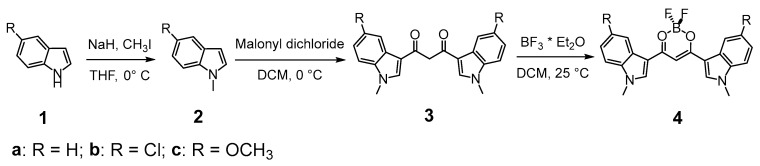
Reaction pathway.

We recently reported on the chemistry of indoles [15,16] and the use of biindole diketones (bdks) as ligands in the synthesis and characterization of biindole diketone coordination complexes [17]. Metalloorganic complexes of these bdks have been proposed as possible next-generation chemotherapeutic drugs to replace cis-platinum derivatives [17]. In this work, we present synthesis and spectroscopic characterization studies of BF_2_bdks having 5,5′-disubstituted 3,3′-biindole-1,3-propanedione (BIP) ligands (see Scheme 1).

Despite their extended π conjugation, bdks are not strongly luminescent for two main reasons: Firstly, the structures of the diketo tautomers are non-planar. Secondly, in the keto-enol tautomer, the activation of very fast, non-radiative decay mechanisms is provided by intra- and intermolecular interactions of the acidic enol proton [18]. Here, it is shown that the bdk fluorescence quantum yield can be greatly enhanced by reacting bdks with boron trifluoride etherate: this enables it to lock the 1,3-diketone into its enolate form and to introduce an acidic, electron-poor center onto an electron-rich backbone. Time-dependent (TD) density functional theory (DFT) calculations have been used to investigate the electronic structure of these new BF_2_bdks, with the aim to assign the peaks observed in the experimental spectra and to explain (i) the red shift of the maximum in the experimental UV-Vis absorption spectra upon difluoroboronation, and (ii) the increase in the fluorescence lifetime of BF_2_bdks with respect to the corresponding bdks. Additionally, we show that these new BF_2_bdks combine the fluorescence properties of the BF_2_bdk core and its ability to generate singlet oxygen with the indole moiety, which, notably, is bioactive.

## 2. Results and Discussion

### 2.1. Synthesis

Friedel–Crafts acylation, a type of electrophilic aromatic substitution, is a well-known and viable strategy for the functionalization of aromatic and heteroaromatic substrates. It allows one to obtain aryl ketones in a single synthetic step starting from a suitable aryl partner and an acyl chloride, catalyzed via a Lewis-acid-like aluminum trichloride. If the aromatic partner is electron-rich, addition of the acid is optional. 

With the idea to combine the indole moiety with the 1,3-diketone nucleus (see Scheme 1), we selected three different indole substrates as starting materials (**1** in Scheme 1): 5H-indole (**a**), 5-chloroindole (**b**), and 5-methoxyindole (**c**). These substrates were chosen as they have a neutral (**a**), a slightly electron-poor (**b**), and an electron-rich molecule (**c**), respectively. The three commercial NH indoles were alkylated at the nitrogen atom (step **1** → **2** in Scheme 1) to protect it from undesired electrophilic attack and unwanted boron complexation, in addition to conferring solubility to end complexes. The use of methyl iodide and sodium hydride as the base in dry THF under an inert atmosphere gave the methylated products in high yields (intermediates **2** in Scheme 1). Diketone synthesis (intermediates **3**) was accomplished using malonyl dichloride, without the addition of AlCl_3_, as already reported by Cirrincione and co-workers [14]. Good yields were obtained for 5H-*N*-methylindole (**3a**) and 5-methoxy-*N*-methylindole (**3c**), while poor results were obtained with 5-chloro-*N*-methylindole (**3b**). To verify if the poor results obtained with the chlorinated compound were associated to the electron poverty of the substrate, we repeated the acylation using an electron-poorer substrate, 5-nitro-*N*-methylindole. In this case, no conversion of the starting material at all was observed. Accordingly, in the literature, there are very few examples of acylation of electron-poor indoles, always requiring very strong Lewis acids such as stannic chloride. The synthesized diketones are labeled in the following ways: HBIP (compound **3a**), HClBIP (compound **3b**), and HBMIP (compound **3c**).

The isolated diketones were then treated with excess boron trifluoride etherate in dichloromethane (step **3** → **4**), causing the precipitation of the corresponding organoboron complex in the reaction mixture after a few minutes of stirring at room temperature. Filtration and washing of the precipitates usually gave the products in a satisfactory yield and good purity for the following spectroscopic analyses. The BF_2_bdks complexes are labeled BF_2_BIP (compound **4a**), BF_2_ClBIP (compound **4b**), and BF_2_BMIP (compound **4c**).

The as-synthesized BF_2_bdks **4** are bright-yellow powders, with an evident fluorescence under a 365 nm UV lamp, both in the solid state and in solution. Compounds bdks **3** and BF_2_bdks **4** were characterized using ^1^H and ^1**3**^C-NMR spectroscopy as well as infrared (IR) spectroscopy (see Supplementary Materials). Further investigation was carried out with ^11^B and ^19^F-{^1^H}NMR spectroscopy (see Supplementary Materials), confirming the structure and purity of the synthesized BF_2_bdks.

**Figure A1:**
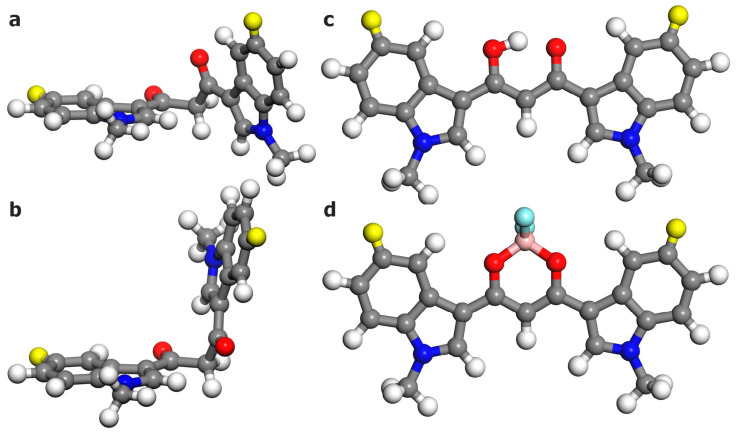
(**a**) *cis*-diketo, (**b**) *trans*-diketo, and (**c**) keto-enol conformers of **3** compounds. (**d**) difluoroboronated compounds **4**. Color code: red (oxygen), pink (boron), blue (nitrogen), bright yellow (R group), light blue (fluoride), grey (carbon), white (hydrogen). Structures as optimized at the B3LYP/def2-TZVPD level of theory for R = H.

Considering the keto-enol tautomerism, each bdk examined in this study is a mixture of three conformers: keto-enol, *trans*-diketo, and *cis*-diketo (see Figure A1c, Figure A1b, and Figure A1a, respectively). The relative amount of these conformers is influenced by different factors, mostly the temperature and the medium (solvents of different polarity, bulk versus gas phase…). ^1^H-NMR indicates a larger concentration of diketo with respect to keto-enol in all the solvents, although an increase in the latter is observed as the solvent polarity rises (see Table 1).

^1^H-NMR spectroscopy of compounds **4** confirms that diketones **3** coordinate the boron difluoride moiety via their enolate form (see Supplementary Materials), where a singlet at 7.1–7.2 ppm can be attributed to enolates C-H. This is also confirmed by comparing IR spectra of compounds **3** and **4**, in which the C=O stretching frequency is redshifted −60 cm^−1^ upon difluoroboronation (Δ${\tilde{\nu }}_{C=O}$, see Figure S1), indicating coordination at the carbonyl group (see Table S1). These results are confirmed via the DFT calculations, predicting a redshift going from **3a** to **4a** (see Section S5 and Figure S2).

### 2.2. UV-Visible Spectroscopy

#### 2.2.1. UV-Vis Absorption Spectroscopy

The UV-Vis absorption spectra of the BF_2_bdks **4a**, **4b**, and **4c** were recorded in a panel of solvents differing as to their polarity and hydrogen bonding properties, namely: toluene (aprotic, non-polar solvent), ethyl acetate, dichloromethane, acetone, acetonitrile (polar solvents, weakly H-bonding acceptors), dimethylformamide, dimethyl sulfoxide (strong H-bond acceptors), butanol, ethanol, and methanol (protic solvents). For details on the sample preparation and measurement apparatuses, see Section 3.4. To evaluate the effects of fluoroboronation on the spectroscopic properties of the compounds, similar measurements were also performed on the corresponding non-modified compounds **3a**, **3b**, and **3c** (see Figure S3).

**Figure 1:**
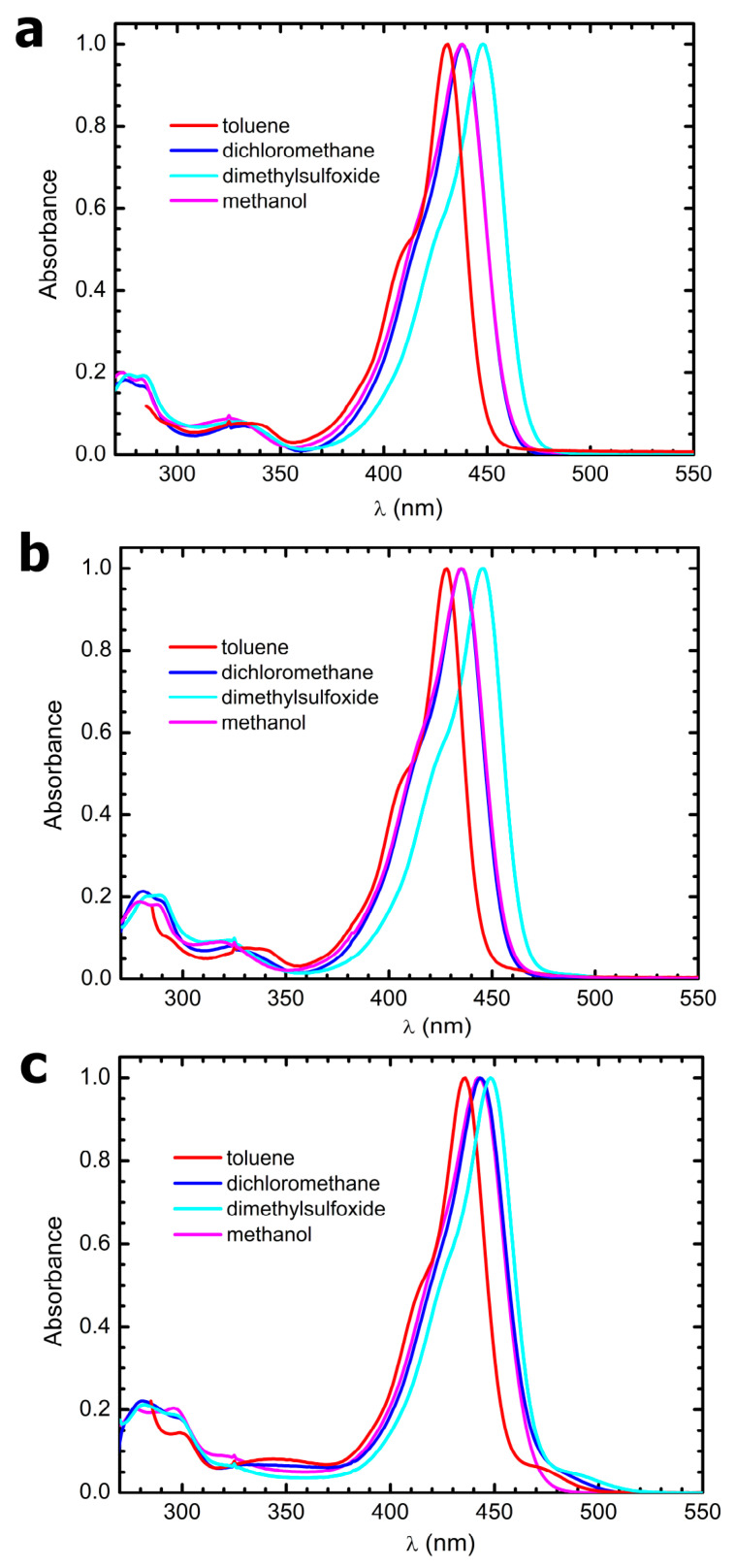
UV-Vis absorption spectra of (**a**) **4a**, (**b**) **4b**, and (**c**) **4c** in toluene (red), dichloromethane (blue), dimethyl sulfoxide (cyan), and methanol (magenta). The intensity of each spectrum is normalized to its maximum intensity.

In Figure 1, we report the UV-Vis absorption spectra of **4a** (a), **4b** (b), and **4c** (c) in toluene, dichloromethane, dimethyl sulfoxide, and methanol. The wavelengths corresponding to the maxima of the absorption spectra (λ_abs_) in all the solvents considered are reported in the third column of Table 2. The value of the molar extinction coefficient at λ_abs_, ε, as determined through linear fit of absorbance vs. concentration plots, is also reported in the fourth column of Table 2 (see Section 3.4 for details).

Like other enolized β-diketones [19,20,21,22,23,24,25,26], the BF_2_bdk compounds show, in all the solvents, a main absorption band peak in the blue portion of the visible spectrum, between 428 nm (**4b** in toluene) and 452 nm (**4c** in dimethyl sulfoxide), and a much fainter absorption in the UVB (around 280 nm). The spectral line shape of the main peak exhibits a substructure, with a shoulder around 400 nm. A systematic redshift is observed for all three compounds on increasing the solvent polarity, and a further bathochromic effect is induced by H-bonding interactions with H-bond-accepting solvents. Nevertheless, λ_abs_ is only slightly dependent on both the environment and the phenolic substituents (see Table 2), unlike what is observed for other classes of BF_2_ complexes [27]. The molar extinction coefficient ε varies by more than a factor of 2 on both the solvent and the phenolic substituent, although a systematic dependence cannot be evidenced. However, the absorption cross-section is notably high in any environment for all the compounds.

In terms of variance, the UV-Vis absorption spectra of the non-fluoroboronated compounds **3a**, **3b**, and **3c** (see Figure S3) are characterized by three bands, two in the UV region, peaking around 255 and 305 nm, and another one in the violet, between 350 nm and 400 nm (see Table 3).

The maximum absorption wavelengths measured for each of the non-fluoroboronated compounds (**3a**, **3b**, and **3c**) in all the solvents are detailed in Table 3. Based on the calculations (see Section 2.3.1), we attribute the UV peaks to absorption of the *trans*-diketo conformer (λ_abs1,diketo_ and λ_abs2,diketo_ in Table 3) and the remaining peak (λ_abs,enol_ in Table 3) to the enol conformer. The blueshifted peak of the enol species suggests reduced conjugation in the non-modified compounds with respect to the fluoroboronated ones. The typical redshift from UV to the blue region of λ_abs_ upon difluoroboronation [10] is, thus, also confirmed for the present class of compounds. The presence of relevant amounts of compounds in the *cis*-diketo conformers, which was hypothesized for other β-diketones [23,24], is excluded in DFT calculations in this instance (see Appendix A). 

By assuming that the value ε(λ_abs2,diketo_)/ε(λ_abs,enol_) = 0.356, calculated using TD-B3LYP-D3 for **3a** in toluene (see Section 2.3.1), does not vary appreciably upon either changing the solvent or shifting from **3a** to the other two compounds, we can estimate the fraction of compound in *trans*-diketo conformation as:

(1) $$f_{i}=\frac{Abs\left(\mathrm{diketo}\right)}{Abs\left(\mathrm{diketo}\right)+0.356\times Abs\left(\mathrm{enol}\right)}$$

where *Abs*(diketo) and *Abs*(enol) represent the absorbances detected at λ_abs2,diketo_ and λ_abs,enol_, respectively, in all solvents but acetone. The *trans*-diketo fractional concentration values, calculated according to Equation (1), are reported in Table 3. This analysis confirm that observed with ^1^H NMR (see Table 1), that the keto-enolic equilibrium is shifted towards the diketo species in all the cases in which it can be estimated (i.e., in any solvent but acetone).

#### 2.2.2. Steady-State Fluorescence Measurements

**Figure 2:**
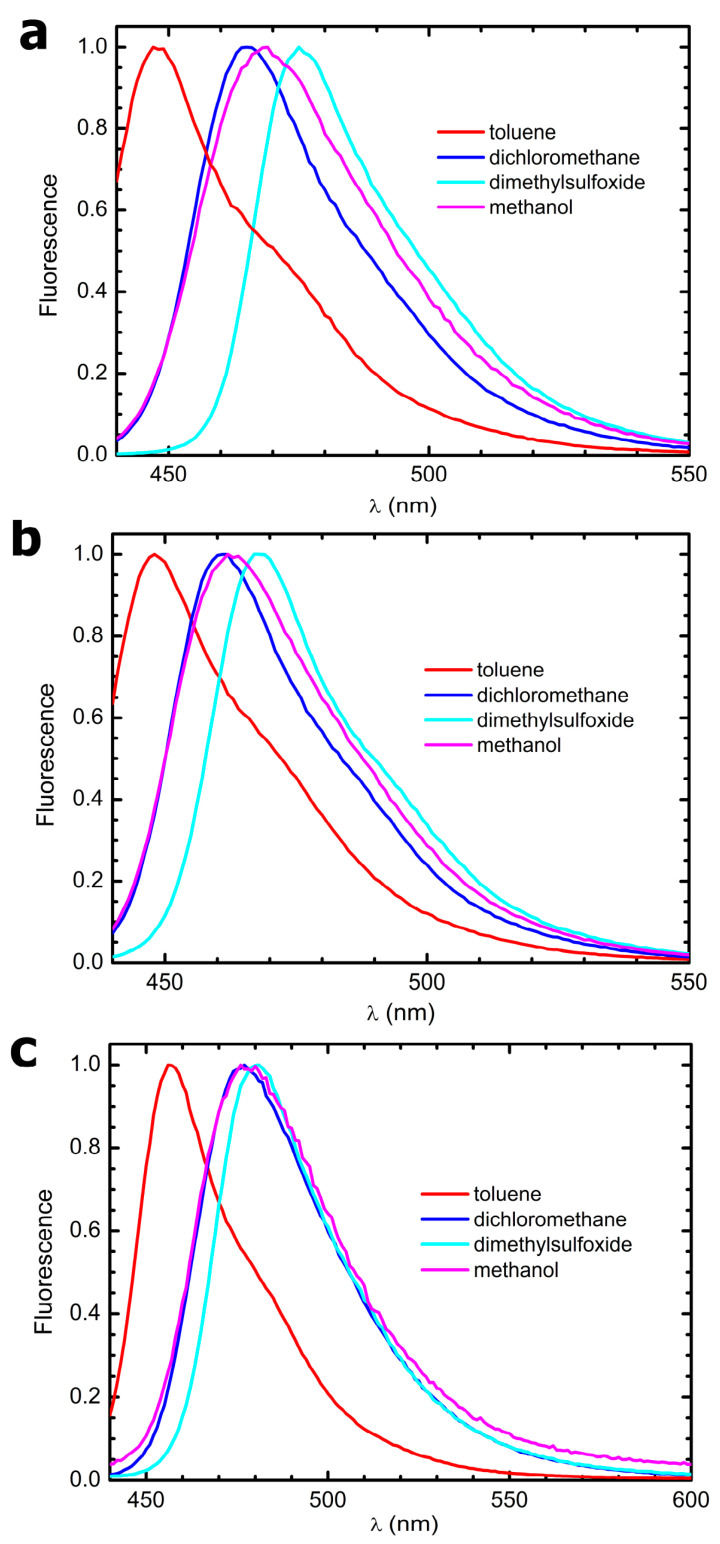
Emission spectra (upon excitation at the pertaining λ_abs_) of (**a**) **4a**, (**b**) **4b**, and (**c**) **4c** in toluene (red), dichloromethane (blue), dimethyl sulfoxide (cyan), and methanol (magenta). The intensity of each spectrum is normalized to its maximum value.

The fluorescence emission spectra of the fluoroboronated compounds **4a**, **4b**, and **4c**, were recorded in all the solvents upon excitation at the pertaining λ_abs_. The emission spectral line shapes in toluene, dichloromethane, dimethyl sulfoxide, and methanol are plotted in Figure 2, for **4a** (upper panel), **4b** (central panel), and **4c** (lower panel), respectively. The maximum fluorescence wavelengths in all the solvents are listed as λ_fluo_ in Table 4. In the same table, the fluorescence quantum yield values, ϕ_fluo_, determined via comparison with dimethyl-POPOP dissolved in cyclohexane (ϕ_fluo_ = 0.95, [26]), are also reported (see Section 3.4 for details on the calculation of ϕ_fluo_).

The emission appears to be poorly solvent-dependent, the only appreciable solvation effect being a sizeable redshift from the non-polar solvent toluene to any of the others.

With the only exception of **4c** in methanol (ϕ_fluo_ = 0.07), fluorescence is extremely intense in any of the tested solvents. Namely, the fluorescence quantum yield value is >0.17 in all the environments.

The compounds **3a**, **3b**, and **3c** emit fluorescence in distinct bands upon excitation at either of the absorption peaks. For all three species, the fluorescence of the diketo conformer is negligibly low. This might be due to a drastically reduced conjugation extent because of the non-planarity of the molecular structure. Excitation at λ_abs,diketo_ resulted in comparably faint emission peaking around 340 nm. The fluorescence quantum yield values could barely be estimated and were lower than 0.01 in all of the tested solvents. The fluorescence peak wavelengths and quantum yields for **3a**, **3b**, and **3c** measured upon excitation at the enol absorption peak, λ_abs,enol_, are listed in Table 5. The emission of the enol fraction of the unmodified compounds is systematically blueshifted with respect to that of the corresponding fluoroboronated species in all the solvents. Moreover, the fluorescence quantum yields, although being notably higher with respect to those measured for the diketo tautomers, which reflects enhanced conjugation in the planar enol conformers, are typically more than one order of magnitude lower than those measured in the case of the fluoroboronated analogues. 

**Figure 3:**
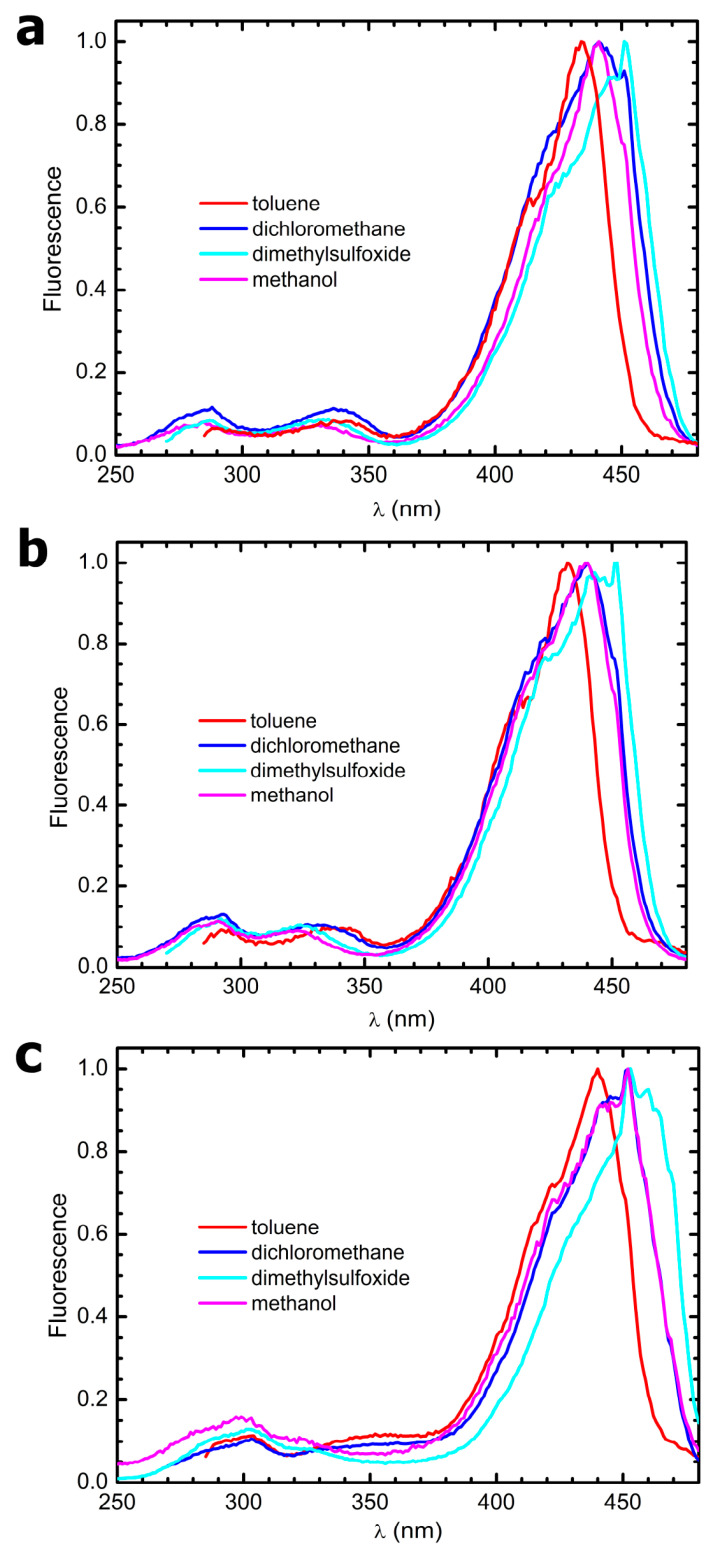
Excitation spectra (observation wavelength set at 500 nm) of (**a**) **4a**, (**b**) **4b**, and (**c**) **4c** in toluene (red), dichloromethane (blue), dimethyl sulfoxide (cyan), and methanol (magenta). The intensity of each spectrum is normalized to its maximum value.

The excitation spectra of the fluoro-boronated compounds were also recorded, with the observation wavelength set at 500 nm. Exemplary spectra are shown in Figure 3. The spectral line shapes, although somewhat redshifted, are very similar to those of the corresponding UV-Vis absorption spectra, suggesting very simple excited-state dynamics.

#### 2.2.3. Time-Resolved Fluorescence Measurements

**Figure 4:**
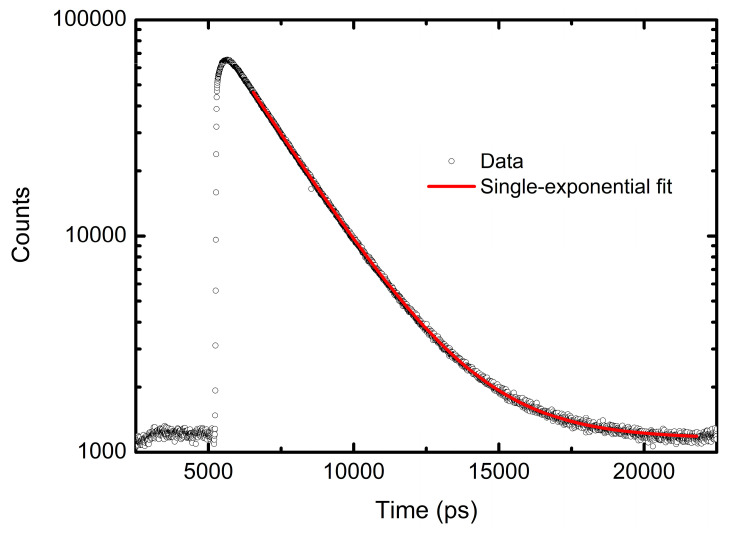
Experimental fluorescence decay pattern of **4a** in dichloromethane (black circles). The solid line represents the best-fitting curve to a single-exponential decay function.

In order to investigate the excited-state dynamics of both the non-substituted compounds **3** and their fluoroboronated analogues **4** in depth, time-resolved fluorescence measurements were performed by exploiting a time-correlated single-photon counting apparatus endowed with 30 ps temporal resolution. For the fluoroboronated species, the decay is single exponential in all the tested solvents. An exemplary decay pattern is plotted in Figure 4. The best-fitting curve is also shown as a solid line. The fluorescence lifetimes of BF_2_bdks in the different solvents are reported in Table 4. In agreement with steady-state fluorescence data, the values of the fluorescence lifetime are high and rather independent from variations in both the polarity and the H-bonding properties of the solvent. Moreover, **4a**, **4b**, and **4c** behave very similarly. This pattern suggests very simple decay dynamics, dominated by radiative decay and basic photophysical mechanisms, such as internal conversion and intersystem crossing. Combining the lifetime data with the measured quantum yield values and exploiting Equations (2), we could estimate the radiative (*k*_Fl_) and non-radiative (*k*_NR_) decay rates, whose values are reproduced in Table 4.

(2) $$k_{\mathrm{Rad}}=\frac{\phi_{\mathrm{Fluo}}}{\tau_{\mathrm{average}}};\;\;\;\;\;\;\;\;\;\;\;k_{\mathrm{NR}}=\frac{1}{\tau_{\mathrm{average}}}-k_{\mathrm{Rad}}$$

The above calculations suggest that the high luminescence of the compounds is due to the concomitance of a fast radiative decay rate with a comparable (thus, unusually slow) non-radiative decay rate. Moreover, also these indirect parameters are rather independent from both environmental properties and substitutions at the lateral aromatic rings.

The situation is much more complicated for compounds of class **3**. Fluorescence decay time distributions were reconstructed both upon excitation at 280 nm (in the absorption spectral region typical of the diketo tautomer) and upon excitation at 420 nm (in the absorption band of the enol conformers). Details on the excitation sources and timing setup are given in Section 3.4. Upon excitation in the UV, biexponential decays were invariably obtained. The values of decay times, *τ_i_*, and relative amplitudes, f_i_, as retrieved from fitting of the experimental decay patterns (see Section 3.4 for details on the fitting procedure), are reported in Table S2. A long-lived component with time constant ≅ 2 ns, similar to that detected for the fluoroboronated compounds, is still observed, but the dominant transient is a much shorter one, in the range 200–400 ps, which we attribute to a non-radiative decay mechanism encompassing photoactivated transition to the enol structures, in analogy to what was observed in other β-diketones [23,24]. The situation is very similar upon excitation at 420 nm (See Table S3), where the 200–400 ps component, ascribable to the well-established reketonization process, occurs with even higher relative amplitude with respect to long-lived radiative decay (i.e., has relatively higher probability to take place than transition from the diketo to the enol structures). This reflects the superior thermodynamic stability of diketo with respect to enol conformers, indicated by UV-Vis absorption data (see Table 3), ^1^H NMR (see Table 1), and demonstrated via DFT calculations (vide Appendix A). Moreover, for all the compounds of class **3**, a very short decay time, not exceeding a few tens of picoseconds, is detected in several solvents. We interpret this transient as due to non-radiative decay through exchange of the enol proton between the keto oxygens. The fact that this mechanism is not always evidenced might relate to a relatively weak ketoenolic intramolecular H-bonding affinity, which might, in turn, explain the unusual diketo-shifted tautomeric equilibrium of these compounds. Calculation of the average excited-state lifetime, *τ_Av_*, according to Equation (3), allows one to apply Equation (2) to estimate *k*_Rad_ and *k*_NR_.

(3) $$\tau_{Av}=\underset{i}{\sum }f_{i}\tau_{i}$$

The estimated rate constants indicate that, although *k*_Rad_ is somewhat reduced in the bdks with respect to BF_2_bdks, the main reason is that the fluorescence quenching reseeds in the activation of efficient non-radiative decay mechanisms, with the overall effect of incrementing *k*_NR_ by roughly two orders of magnitude.

According to the above-proposed rationale, the dramatic increase in fluorescence quantum yield observed upon fluoroboronation may be ascribed to the concomitant hindrance of photoinduced tautomerization and excited-state intramolecular proton transfer dynamics.

#### 2.2.4. Singlet Oxygen Production

The generation of ^1^O_2_ upon irradiation with UV-Vis light is the main source of the photobiological activity of a class of drugs named Type II photosensitizers. Upon electronic excitation, these pharmaceutical active principles undergo intersystem crossing to the metastable T_1_ state lying beneath the fluorescent singlet and the electronic ground state. As the molecular oxygen ground state is a spin triplet, collisional quenching between a photosensitizer and oxygen results in a selection-rules-allowed concomitant transition of the drug to the ground state and of oxygen to its spin-singlet radical form. This latter is extremely reactive. Accordingly, if produced within biological tissues, it is capable of inducing severe oxidative stress, thereby triggering apoptosis as well as other cell death mechanisms. Because the ability of a putative drug substance to act as Type II photosensitizer ultimately depends on the relative probability to decay through intersystem crossing, stabilizing the *S*_1_ state with respect to other non-radiative decay pathways may be beneficial to the optimization of the photopharmaceutical performance of a molecule. In this view, as a preliminary evaluation of the potential of the compounds of class **4** as photosensitizers, the ^1^O_2_ generation quantum yields, ϕ(^1^O_2_), of solutions of **3a**, **3b**, **3c**, **4a**, **4b**, and **4c** were assessed relative to that of meso-tetraphenylporphyrin tetrasulphonate (TSPP), a well-characterized photosensitizer currently undergoing clinical trials [28]. The amount of produced ^1^O_2_ was estimated in terms of the oxidation-induced quenching of the 9,10-dimethylanthracene fluorescence [29]. Singlet oxygen quantum yields are reported in Table 6 (see Section 3.6 for details on the experimental procedures).

While none of the bdk compounds exhibit significant ^1^O_2_ generation efficacy, the corresponding BF_2_bdks all display sizeable photosensitizing activity, as expected based on what was reported previously for other BF_2_bdks [2]. Although the activity of compounds **4** is lower than that of TSPP, these results confirm the effectiveness of the fluoroboronation strategy to achieve excited-state stabilization and, thereby, the enhancement of intersystem crossing dynamics.

### 2.3. DFT Calculations

Cartesian coordinates for all optimized geometries can be found as XYZ files in the Supplementary Materials.

#### 2.3.1. UV-Vis Absorption Spectra

All the functionals adopted in the study provided a qualitatively identical description of the modification of the UV-Vis spectra upon difluoroboronation and functional group on the indole. The best quantitative agreement between the computed and experimental spectra was obtained using B3LYP-D3 without TDA. For this reason, the results obtained with this level of calculation are discussed later, while the results obtained with the other methods are reported in the Supplementary Materials (see Figures S4–S8 and Tables S4–S8). This result was unexpected based on a recent assessment of several density functionals for the prediction of excitation energies of most organic and main-group molecules [30]. In this assessment, ωB97X-D was the functional characterized by a lower root mean square error in the prediction of the excitation energies, while B3LYP performed poorly. Here, the trend is the opposite. Interestingly, although the HOMO-LUMO band gap (Δ*E*_HOMO-LUMO_) is a rough approximation of the excitation energy, its value computed with B3LYP-D3 almost coincides with the excitation energy (see Table 7), while all the other functionals largely overestimate it (see Tables S4–S8). This result agrees with what was reported for other compounds [31,32]. For what concerns the TDA approximation, it introduces a negligible error with respect to TD, underestimating λ_abs_ of an additional 10–15 nm. Because of the lower computational cost of TDA calculations, it is confirmed to be a suitable approximation for the modeling of excitation energies also for bdks and their complexes.

**Figure 5:**
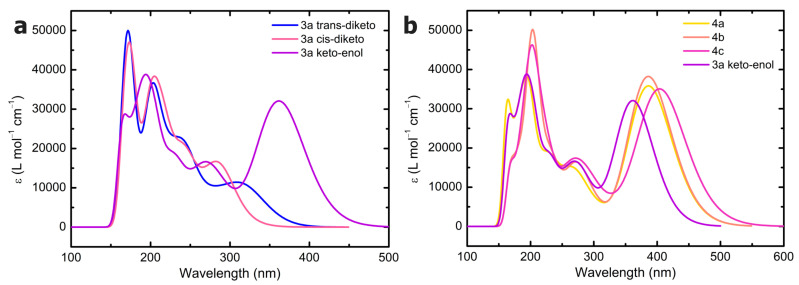
Electronic absorption spectra of (**a**) the three conformers of **3a** and (**b**) the BF_2_bdks compounds in their ground state as computed at the TD-B3LYP-D3/def2-TZVPD, including the first 100 excitations.

The computed UV-Vis absorption spectra of the three conformers of **3a** are reported in Figure 5a, while the spectra of *trans*-diketo **3a**, **4a**, **4b**, and **4c** are shown in Figure 5b.

For what concerns the three conformers of **3a**, their computed spectra are significantly different for wavelengths > 270 nm: the keto-enol has a maximum at 362 nm (see Table 7), while the *trans*-diketo and the *cis*-diketo peak at 309 and 283 nm, respectively. Based on the relative energy stability of the three **3a** conformers, both in the ground and first excited state (see Appendix A, Table A1 and Table A2), we expect to observe signals associated only to *trans*-diketo and keto-enol conformers in the experimental spectra. The computed spectra for the keto-enol and the *trans*-diketo match the signals recorded experimentally in toluene (see Table 3 and Figure S3), supporting the assignments discussed in Section 2.2.1. While the band at 362 nm (oscillator strength *f*_absB3_ = 0.70) of the keto-enol is determined to be associated to a single transition, the band at 309 nm of *trans*-diketo is the convolution of three signals at 331.34 (*f*_absB3_ = 0.08), and two almost degenerate at 314.30 (*f*_absB3_ = 0.02) and 314.28 nm (*f*_absB3_ = 0.14). The signal observed in the experiments at ~245 nm is associated based on the calculations to a higher-energy electronic transition of *trans*-diketo (excited state 12, 247.9 nm, *f*_absB3_ = 0.23).

**Figure 6:**
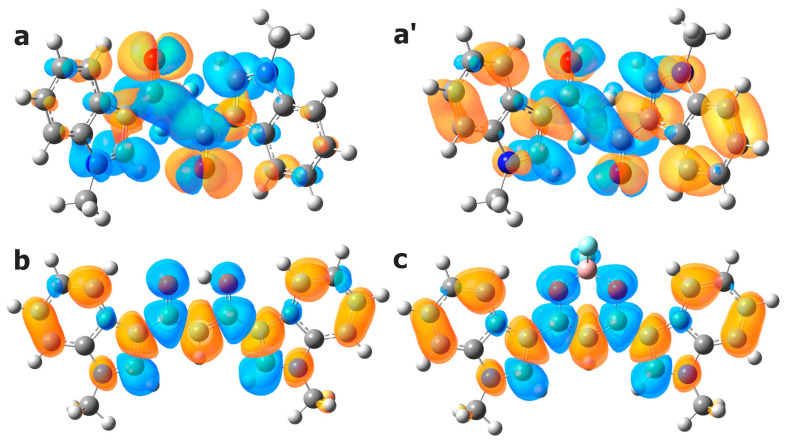
Difference of the total density of the first excited state and the ground state in (**a**) **3a** *trans*-diketo, (**b**) **3a** keto-enol, and (**c**) **4a** computed at the B3LYP-D3/def2-TZVPD. (**a’**) The third excited state of *trans*-diketo is also shown for the sake of completeness. Blue region: positive values, corresponding to an increase of the total electronic density in the excited state with respect to the ground state. Orange regions: negative values. Isosurface at 0.0006 a.u. Color code of the atoms: red (oxygen), pink (boron), blue (nitrogen), bright yellow (R group), light blue (fluoride), grey (carbon), white (hydrogen).

The difference between the total electronic density of the first excited state and of the ground state is shown for **3a** *trans*-diketo (331 nm) and **3a** keto-enol (362 nm) in Figure 6a,b, respectively. As the oscillator strength of the transition to the third excited state is double that of the first excited state for *trans*-diketo, the corresponding total density difference is also reported in Figure 6a’. For both conformers, the electronic transitions correspond to a transfer of the charge from the indole and the CH_2_/CH group of the 1,3-dicarbonyl to the C=O/C-O(H). While for **3a** *trans*-diketo, the transition causes a charge transfer at short range, often on the same atom (see, for example, the oxygen atoms in Figure 6a,a’), for **3a** keto-enol, the charge transfer involves different atoms, i.e., in the excited state, the regions with a lower electron density than the ground state (orange background in Figure 6b) are localized on different atoms than the regions with a higher electron density (blue background). The larger charge displacement is at the origin of the larger ground to excited state transition electric dipole moment ($\mu_{S0\to S1}$) of the first electronic transition in **3a** keto-enol than **3a** diketo (see Table 2 and Tables S4–S8).

After functionalization with BF_2_, the experimental spectra show the typical redshift of the absorption maxima from the UV to the visible region, as reported in the literature [10]. The TD-DFT spectra reproduce the shift in the absorption maximum, going from **3a** (keto-enol, dark-violet curve in Figure 5b) to **4a** (yellow curve). The computed spectra for compounds **4** show the same line shape of the **3a** keto-enol spectrum, although redshifted (see Figure 5b). The maximum absorption wavelength in toluene is experimentally observed at 431 nm for **4a**, while it is slightly blueshifted to 428 in **4b** and slightly redshifted to 436 nm for **4c** (see Table 2). These changes are qualitatively supported by the calculations, with corresponding computed values of 386.6 (**4a**, R = −H, *f*_absB3_ = 0.88), 386.2 (**4b**, R = −Cl, *f*_absB3_ = 0.94), and 405.2 nm (**4c**, R = −OCH_3_, *f*_absB3_ = 0.85), respectively. This small effect of the R group on the excitation energies is supported by the CM5 charges computed on the BF_2_ unit and the 1,3-dicarbonyl that are very similar in all the BF_2_bdks. The experimental band observed at ~430 nm for compounds **4** is associated based on the TD-DFT calculations to a single transition. The shoulder at 400 nm is assigned alternatively to a vibronic profile or to local distortion of the orientation of the two indoles from the full planarity (see Section 2.3.2).

Figure 6c shows, for **4a,** the effect on the electronic total density of the electronic excitation corresponding to the experimental 431 nm signal. This plot is essentially identical to the one obtained for **3a**, showing a charge transfer from the indole to the carbonyls and the fluorine. The computed oscillator strengths for compounds **4** are larger than those computed for the corresponding bdk compounds. While for the keto-enol forms, the increase in *f*_absB3_ is only 25%, for the *trans*-diketo, i.e., the majority species in the experiments, it is 500%. The larger *f*_absB3_ in compounds **4** can be explained by the larger charge displacement occurring in the excitation, going, in order, from *trans*-diketo (Figure 6a,a’) to keto-enol (Figure 6b) to BF_2_ complexes (Figure 6c). Based on Figure 6, a larger displacement of the charge is obtained in BF_2_bdks than in bdks, even considering the keto-enol conformers because of the involvement of the fluorine atoms. This results in larger $\mu_{S0\to S1}$ and then oscillator strengths: $\mu_{S0\to S1}$ computed for **3a** (keto-enol) and **4a** are, in fact, 9.4 and 11.2 a.u., respectively (see Table 7).

#### 2.3.2. UV-Vis Emission Spectra

**Figure 7:**
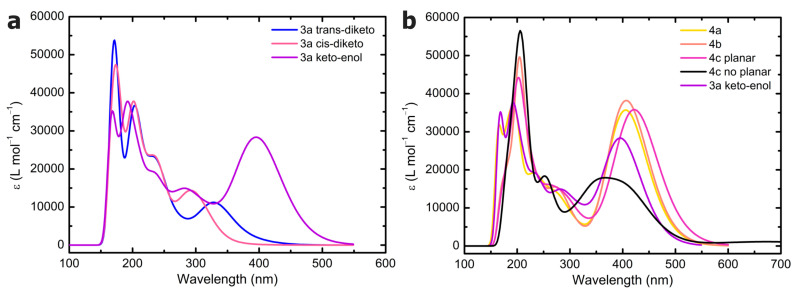
Electronic fluorescence spectra of (**a**) the three conformers of **3a** and (**b**) the BF_2_bdks compounds as computed at the TD-B3LYP-D3/def2-TZVPD level including the first 100 excitations. Two spectra are reported for **4c**: the spectrum obtained on the *S*_1_ geometry optimized with TD-B3LYP-D3 (**4c** no planar, see Figure S11) and with TDA-CAM-B3LYP-D3 (**4c** planar).

The optimized geometry for the first excited state of all the compounds is very similar to the one obtained for the ground state at M06-2X, ωB97X-D, and CAM-B3LYP-D3. TDA-B3LYP-D3 and TD-B3LYP-D3 instead predicted that, as result of the first excitation, the compound structure goes from a geometry having the two indole coplanars in *S*_0_ (as in Figure A1c) to a geometry very similar to *trans*-diketo in *S*_1_ for **3a** keto-enol, **4a**, **4b**, and **4c** using TDA-B3LYP-D3 and **4c** using TD-B3LYP-D3 (see Figure S11). The coplanar conformers in *S*_1_ are computed to be less stable by ~20 kJ mol^−1^, while the other levels of calculations agree in predicting the two indoles coplanar also in *S*_1_. For what concerns **3a** *cis*-diketo, the first excited state for these functionals will undergo cyclization. These results are independent on the number of states included (up to 100), the maximum of the optimization step, and the presence/absence of D3 correction. The experimental emission and excitation spectra support the similarity of the ground and excited state geometry for all the compounds and then a planar geometry for **3a** keto-enol, **4a**, **4b**, and **4c** in *S*_1_. The computed emission spectra for a non-planar geometry for compounds **4** would be significantly different than the corresponding absorption spectra (see the emission spectra of the two **4c** conformers in Figure 7). Reoptimization of the non-planar conformers of **3a** keto-enol, **4a**, **4b**, and **4c** in *S*_1_ using M06-2X, ωB97X-D, and CAM-B3LYP-D3 restores the planar geometry. Based on this assessment, B3LYP-D3 seems to not be a suitable method for the optimization of excited states for the bdks and BF_2_bdks class. Nevertheless, TD-B3LYP-D3 level provides the transition energies closest to the experimental values, as already noted for the absorption spectra. In order to facilitate a comparison with the experiments, the results reported in the following for *S*_1_ were obtained using TD-B3LYP-D3, using single-point calculations for **3a** *cis*-diketo and for **4c** on the *S*_1_ TDA-CAM-B3LYP-D3-optimized geometries. The emission data obtained for TDA-B3LYP-D3, TDA-M06-2X, TD-ωB97X-D, TDA-ωB97X-D, and TDA-CAM-B3LYP-D3 are reported in the Supplementary Materials.

All the methods agree with the experiments in the qualitative description of the effect of difluoroboronation and of the chemical modification of the indole on the emission spectra. The calculations quantitatively reproduce the shift in the emission bands with respect to the absorption spectra for all the compounds. While for **3a**, a redshift is predicted of 53–16 cm^−1^, for the BF_2_ complexes, the shift is about +20 cm^−1^, independently of the R group. The wavelengths corresponding to the maximum of the emission spectra in the >350 nm region are also very similar for all the BF_2_bdks, as verified for the absorption spectra: 406.0 (**4a**, R = −H, *f*_absB3_ = 0.88), 407.1 (**4b**, R = −Cl, *f*_absB3_ = 0.94), and 422.9 nm (**4c**, R = −OCH_3_, *f*_absB3_ = 0.87), respectively (see also Figure 7 and Figures S4–S8, Table 7 and Tables S4–S8). Again, very similar charges are computed on the BF_2_ unit and the 1,3-dicarbonyl also in the excited state, indicating a poor influence of the R group on the electronic properties of the compounds. The computed radiative rate constants (*k*_rad_, see Table 7 for B3LYP-D3 and Tables S4–S8 for the other methods) are able to reproduce the increase of one order of magnitude in the experimental *k*_Rad_ upon difluoroboronation (see *k*_Rad_ in Table 4 and Table 5). Diketo and the keto-enol conformers of bdks have very different computed electronic properties, with the keto-enol having an extinction coefficient and radiative decay rate associated with the first excitation energy one order of magnitude larger than diketo ones. Thus, the increase in the experimental *k*_Rad_ upon difluoroboronation is a further confirmation of the larger concentration of diketo with respect to keto-enol conformers in the solutions. In fact, *k*_radDFT_ for **3a** keto-enol is only slightly lower than the **4a** one, and both are one order of magnitude larger than **3a** *trans*-diketo. This result also explains the increase in *k*_Rad_ with the solvent polarity in compounds **3** (see Table 4), as associated with the increase in the relative concentration of keto-enol, as actually quantified with ^1^H NMR (see Table 1).

The increase in the fluorescence properties of BF_2_bdks can then mainly be associated with the stabilization of the keto-enol geometry. A positive contribution is also brought about by the introduction of the BF_2_ unit (although less important than the stabilization of a planar and more symmetric geometry), and it is associated with a decrease in the band gap with respect to the keto-enol conformers and an increase in the transition electric dipole moment. The increase in the dipole moment is explained by total density maps, showing a larger charge displacement in the first electronic transition in BF_2_bdks than in bdks because of the involvement of the BF_2_ unit (see Figure 6). Confirming the larger role played by the symmetry of the geometry in enhancing the fluorescence properties of these compounds, the lowest-energy band of the emission spectrum of a distorted **4c** conformer (see Figure S11) is associated with five excitations (λ_fluoB3_ in nm, *f*_fluoB3_ in a.u.): (674.95, 0.03), (450.25, 0.03), (409.13, 0.31), (366.22, 0.01), and (349.96, 0.32). All these bands have a lower *f*_fluoB3_ than the corresponding planar conformer (0.87 a.u., see Table 7).

## 3. Materials and Methods

### 3.1. Materials

All reactions were performed in oven-dried glassware under normal atmosphere, unless otherwise noted. All reagents and solvents were purchased from Fluorochem, TCI, or Merck and used without any further purification. Where specified, reactions were monitored via thin-layer chromatography (TLC) on POLYGRAM^®^ Xtra Sil G/UV254 (0.2 mm layer thickness; Macherey-Nagel); spots were observed under a UV lamp at 254 or 365 nm. Gravimetric column chromatography was performed using silica gel (60 Å, particles size: 0.63–0.2 mm) as stationary phase. ^1^H, ^1**3c**^, ^19^F, and ^11^B NMR spectra were acquired on a Bruker AVANCE I 400 instrument at 400.13, 100.61, 376.5, and 128.38 MHz, respectively, and are referenced using residual non-deuterated solvents (CHCl_3_ 7.26 ppm in CDCl_3_, DMSO 2.50 ppm in d_6_-DMSO); deuterated solvents were purchased from Eurisotop; multiplicities are abbreviated as singlet (s), doublet (d), doublet of doublets (dd), and triplet (t); coupling constants are reported in Hz; IR spectra were acquired in attenuated total reflectance (ATR) mode using a FT-IR Thermo Scientific Nicolet iS10 Smart iTR instrument equipped with a diamond optical element, over a range 4000–650 cm^−1^ (at 4 cm^−1^ resolution). Intensities are denoted as: br = broadened signal, sh = shoulder, vs = very strong, s = strong, m = medium, w = weak and vw = very weak.

### 3.2. Synthesis of 1, 2, and 3 Compounds

Thus, **1a**, **1b**, **1c**, and *N*-Methyl-1*H*-indole **2a** were used as received without further purification (see Supplementary Material).

Synthesis of *N*-methyl indoles **2b** and **2c** and diketones HBIP **3a**, HClBIP **3b,** and HBMIP **3c** followed a reported literature procedure (see Ref. [14] and Section S1 in the Supplementary Materials).

### 3.3. Synthesis of the Complexes

#### 3.3.1. 1,3-Bis(1-methyl-3-indolyl)propane-1,3-dione Difluoroborate (**4a**)

To a solution of HBIP (100 mg, 0.30 mmol) in dichloromethane (100 mL), BF_3_·Et_2_O (1.1 mL, 9.0 mmol) is added, and the mixture is stirred at rt for 5 min: a colored precipitate forms. The solid is filtered on a Buchner filter funnel and washed with cold dichloromethane and then dried under vacuum. BIP difluoroborate is obtained as an orange solid (73 mg, 64% yield). ^1^H-NMR (d_6_-DMSO) δ = 8.77 (s, 2H), 8.19 (m, 2H), 7.67 (m, 2H), 7.38 (m, 4H), 7.18 (s, 1H, enolate C-*H*), 3.97 (s, 6H, N-C*H_3_*). ^1**3c**^-NMR (d_6_-DMSO) δ = 174.8 (+), 138.8 (−), 137.9 (+), 125.1 (+), 123.5 (−), 122.9 (−), 121.4 (−), 111.6 (−), 109.4 (+), 91.0 (−), 33.8 (−). ^11^B-NMR (d_6_-DMSO) δ = 0.91 (br s). ^19^F-NMR (d_6_-DMSO) δ = −142.6 (br s), −142.7 (br s). IR (ATR) ῦ (cm^−1^) = 3567 (br, vs), 3134 (vw), 3053 (vw), 2899 (vw), 1610 (w), 1583 (s), 1560 (vs), 1538 (vs), 1527 (vs), 1510 (br, vs), 1489 (sh, s), 1464 (vs), 1452 (sh, s), 1411 (vw), 1384 (s), 1363 (s), 1313 (s), 1307 (s), 1257 (vw), 1235 (s), 1176 (sh, m), 1165 (s), 1148 (s), 1128 (vs), 1122 (vs), 1087 (vs), 1054 (m), 1015 (w), 967 (s), 944 (w), 916 (w), 884 (w), 841 (vs), 780 (w), 766 (w), 750 (vs), 744 (vs).

#### 3.3.2. 1,3-Bis(5-chloro-1-methyl-3-indolyl)propane-1,3-dione Difluoroborate (**4b**)

To a solution of HBClIP (**3b**) (100 mg, 0.25 mmol) in dichloromethane (100 mL), BF_3_·Et_2_O (1 mL, 7.8 mmol) is added, and the mixture is stirred at rt for 5 min: a colored precipitate forms. The solid is filtered on a Buchner filter funnel and washed with cold dichloromethane. The residue is purified via column chromatography (DCM, R_f_ = 0.45) to obtain BClIP difluoroborate as an orange solid (73 mg, 68% yield). ^1^H-NMR (d_6_-DMSO) δ = 8.59 (s, 2H, *H2*), 8.12 (d, ^4^J = 2.05 Hz, 2H, *H4*), 7.71 (d, ^3^J = 9.0 Hz, 2H, *H7*), 7.4 (dd, ^3^J = 9.0 Hz, ^4^J = 2.2 Hz, 2H, *H6*), 7.20 (s, 1H, enolate C-*H*), 3.97 (s, 6H, N-C*H3*). ^1**3c**^-NMR (d_6_-DMSO) δ = 174.8 (+), 140.1 (−), 136.5 (+), 127.8 (+), 126.1 (+), 123.5 (−), 120.5 (−), 113.5 (−), 91.1 (−), 40.4 (−). ^11^B-NMR (d_6_-DMSO) δ = 0.85 (br s). ^19^F-NMR (d_6_-DMSO) δ = −139.3 (br s), −139.4 (br s). IR (ATR) ῦ (cm^−1^) = 3123 (vw), 1616 (vw), 1558 (br, vs), 1527 (s), 1509 (m), 1466 (m), 1450 (m), 1428 (w), 1424 (vw), 1393 (vs), 1377 (m), 1368 (m), 1348 (vw), 1341 (vw), 1328 (s), 1296 (s), 1239 (sh, w), 1230 (s), 1177 (w), 1143 (m), 1137 (m), 1106 (s), 1078 (m), 1063 (m), 1034 (s), 1018 (sh, w), 993 (w), 988 (w), 928 (w), 919 (vw), 886 (w), 875 (w), 852 (w), 845 (vw), 826 (w), 802 (w), 793 (m), 783 (m), 777 (m), 736 (w), 683 (m), 638 (w).

#### 3.3.3. 1,3-Bis(5-methoxy-1-methyl-3-indolyl)propane-1,3-dione Difluoroborate (**4c**)

To a solution of HBMIP (**3c**) (100 mg, 0.26 mmol) in dichloromethane (100 mL), BF_3_·Et_2_O (1 mL, 7.80 mmol) is added, and the mixture was stirred at rt for 5 min: a colored precipitate forms. The solid is filtered on a Buchner filter funnel, washed with cold dichloromethane, and then dried under vacuum. BMIP difluoroborate is obtained as an orange solid (80 mg, 70%). ^1^H-NMR (d_6_-DMSO) δ = 8.64 (s, 2H, H2), 7.62 (d, ^4^J = 2.3 Hz, 2H, H4), 7.57 (d, 3J = 8.9 Hz, 2H, H7), 7.08 (s, 1H, enolate C-H), 7.02 (dd, 3J = 8.9 Hz, 4J = 2.4 Hz, 2H, H6), 3.93 (s, 6H), 3.85 (s, 6H). ^1**3c**^-NMR (d_6_-DMSO) δ = 174.5 (+), 156.2 (+), 138.6 (−), 132.9 (+), 126 (+), 112.4 (−), 112.3 (−), 109 (+), 104.2 (−), 90.6 (−), 55.5 (−), 33.9 (−). ^11^B-NMR (d_6_-DMSO) δ = 0.88 (br s). ^19^F-NMR (d_6_-DMSO) δ = −139.8 (br s), −139.9 (br s). IR (ATR) ῦ (cm^−1^) = 3131 (vw), 2946 (vw), 1619 (w), 1575 (sh, m), 1554 (br, vs), 1523 (vs), 1471 (vs), 1459 (sh, s), 1440 (m), 1425 (w), 1391 (vs), 1367 (s), 1345 (vw), 1330 (m), 1298 (m), 1282 (m), 1273 (m), 1228 (sh, m), 1221 (s), 1212 (sh, m), 1143 (w), 1126 (m), 1096 (s), 1060 (m), 1024 (m), 984 (m), 894 (w), 860 (vw), 850 (w), 811 (w), 800 (w), 777 (m), 736 (vw), 702 (sh, m), 698 (s).

### 3.4. Steady-State Electronic-State Transition Spectroscopy Measurements

The UV-Vis absorption spectra were recorded with a Perkin Elmer Lambda2 spectrophotometer. The fluorescence emission and excitation spectra were recorded with a PTI fluorescence master system spectrofluorimeter. The instrument was interfaced with the acquisition software Felix 2000 (version 1), which performed online correction of the data with respect to the excitation lamp spectral radiance and detector spectral quantum efficiency.

The solvents used to prepare the solutions for all electronic-state transition spectroscopy experiments were purchased from Merck and were of HPLC grade. They were used without further purification. Due to the poor solubility of the compounds in some of the solvents, a concentrated stock in acetone was prepared weighing some powder and dissolving it in 3 mL of this solvent. Afterwards, 50 µL aliquots of this stock were put in glass vials and let to evaporate for 48 h at 50 °C. Finally, the as-obtained films were resuspended in 3 mL of the desired solvent, and the samples were sonicated for 1 h to favor disaggregation. From this procedure, clear solutions were obtained in all solvents.

Molar extinction coefficients were estimated by performing linear regressions on absorbance vs. concentration plots, obtained through progressive dilution of the above stock solutions to one-tenth of the initial concentration in several steps (N > 5). An exemplary plot is reported in Figure S10.

Fluorescence quantum yields, ϕ_Fluo_, were determined by comparing the integrated fluorescence intensity with that of dimethyl-POPOP, a dye used in particle physics as a reactant of scintillator chambers. Due to its high-fluorescence quantum yield, which is tabulated to be ϕ_Fluo_ = 0.95 when dissolved in cyclohexane and excited in its UVA absorption band [26], dimethyl-POPOP has been also widely used as a fluorescence standard since the 1970s. Fluorescence was elicited exciting the samples at their absorption maxima; the emission spectra were normalized with respect to their peak absorbances. Fluorescence quantum yield values were corrected for the solvent’s refractive indexes.

### 3.5. Time-Resolved Fluorescence Measurements

The time-resolved fluorescence decay patterns of the compounds in solution were reconstructed referring to the time-correlated single-photon counting (TCSPC) technique. The experimental apparatus exploited to this aim is fully described elsewhere [23,24]. Its overall time resolution can be estimated in terms of the temporal point-spread function (TPSF), i.e., the temporal profile of the excitation laser pulses directly sent to the detector-sensitive area, as reconstructed by the TCSPC system. Typical TPSF at different wavelengths is reproduced in Figure S9. For the experiments reported hereby, the excitation source was a Ti/saffire cw-mode-locked laser (mod. Tiger-ps, Time Bandwidth Products, Zurich, CH), delivering pulses at 48 MHz repetition rate of 3.9 ps duration at the fundamental wavelength. The samples were excited at 420 nm, in proximity of the enol tautomers absorption peak, by the built-in second harmonic of the beam. To elicit the diketo tautomer fluorescence in the non-fluoroboronated compounds **3a**, **3b**, and **3c**, the third harmonic was generated out of cavity, as described elsewhere [23,24]. The fluorescence photons were detected by means of an MPD50 single-photon avalanche diode with integrated active quenching and cooling circuitry and timed by a single module of an SPC-152 integrated board (Becker & Hickl GmbH, Berlin, Germany). The decays were fitted to single-, double-, or triple-exponential model functions exploiting the Levenberg–Marquardt algorithm built in the software Origin 7 for parameter optimization. The number of components of each decay was established adding transients one by one until addition of further decay components did not lead to improvement in the fit quality (evaluated in terms of Χ^2^ value and fit residuals). The data were acquired in triplicate, and the fitting parameters reported in this article are averages over the three parallels. The errors are expressed in terms of the pertaining standard deviations.

### 3.6. Estimation of Singlet Oxygen Generation Efficiency

The improved efficiency in photosensitizing singlet oxygen (^1^O_2_) generation of the BF_2_bdk with respect to the corresponding bdk compounds was assessed in ethanol (the most biocompatible solvent in our panel) by exploiting the DMA fluorescence assay [29]. Due to the marked tendency of this sensor to oxidate, a fresh 1 mM concentrated stock of DMA in ethanol was prepared from the powdered compound immediately before each singlet oxygen generation measurement according to the procedure detailed elsewhere [25]. Solutions of the BF_2_bdk at approximate concentration of 1 µM were prepared and their exact concentration determined spectrophotometrically using the molar extinction coefficients reported in Table 2. Due to the impossibility of deriving the precise concentration of bdks in the enol conformers, we chose to assess the ^1^O_2_ generation of solutions of these latter compounds having the same absorbance value (≅0.05) at the enol absorption peak. Finally, the ability of BF_2_bdks to quench the DMA fluorescence (i.e., to generate ^1^O_2_) was compared to that of a 1 µM concentrated solution of TSPP in water. The exact concentration was assessed spectrophotometrically assuming the molar extinction coefficient value ε(413 nm) = 129,000 M^−1^cm^−1^ at the Soret band peak [28]. DMA was added from the 1 mM stocks at a final concentration of 20 μM. The obtained samples were placed in 1 × 1 cm^2^ fluorimetry quartz cells to carry out the fluorescence measurements. The DMA fluorescence was excited in correspondence of its 340 nm absorption peak and measured in the band 390–650 nm using the PTI spectrofluorimeter described in Section 3.4, prior to and after photosensitization of ^1^O_2_ through irradiation of the solutions for 30 s with a Wood lamp. Control measurements were carried out in the same experimental conditions on DMA alone.

### 3.7. Density Functional Calculations

All DFT calculations were performed using the Gaussian 16 program [33]. Four density functionals were considered: M06-2X [34], ωB97X-D [35], B3LYP-D3, and CAM-B3LYP-D3, i.e., B3LYP [36,37] and CAM-B3LYP [38], including the D3 version of Grimme’s dispersion (D3) with Becke–Johnson damping (BJ) [39]. M06-2X and ωB97X-D have been shown to be among those having the highest accuracy in a recent assessment performed by Liang et al. [30] for the determination of electronic excitations in the UV-Vis region in organic and main-group molecules. They were used in combination with the triple zeta def2-TZVPD basis sets [40,41,42], as suggested in Ref. [30], although identical results were obtained for B3LYP-D3 in combination with def2-TZVP basis sets. A (99,590) pruned grid was used (i.e., 99 radial points and 590 angular points per radial point), corresponding to the grid = ultrafine option. 

Geometry optimization was carried out by means of the Berny optimization algorithm with analytical gradient and default convergence thresholds. Unscaled, harmonic vibrational frequencies were computed analytically. Zero-point vibrational energies (ZPVE) were calculated at 1 atm and 298 K from conventional ideal gas, rigid rotator, particle in a box, and quantum mechanical harmonic oscillator partition functions. Charge and spin densities were obtained using Charge Model 5 (CM5) [43] and Hirshfeld population analysis [44], respectively.

Time-dependent (TD) density functional theory in the frame of the linear response theory [45] is the most common approach for the study of molecular electronic excited states [46,47]. All the calculations were performed using the Tamm–Dancoff approximation (TDA, [30,48]). Liang et al. [30] showed that TDA offers a good balance between computational efficiency and accuracy. For comparison, B3LYP-D3 and ωB97X-D calculations have also been performed without TDA (TD-B3LYP-D3). The electronic absorption (emission) spectra were computed by single-point calculations on the ground (first excited)-state-optimized structures by considering the first 100 excitations. All the calculations were performed on the gas phase single molecule, other than for the evaluation of the relative stability of **3a** conformers, where a polarizable continuum toluene model was also adopted (ε = 2.3741 [49]). A Gaussian broadening was applied to the TDDFT excitations using a standard deviation of 0.3 eV. No scaling factors were adopted. Visualization of the computed spectra and of the total density maps was performed using GaussView 6.0.

**Figure 8:**
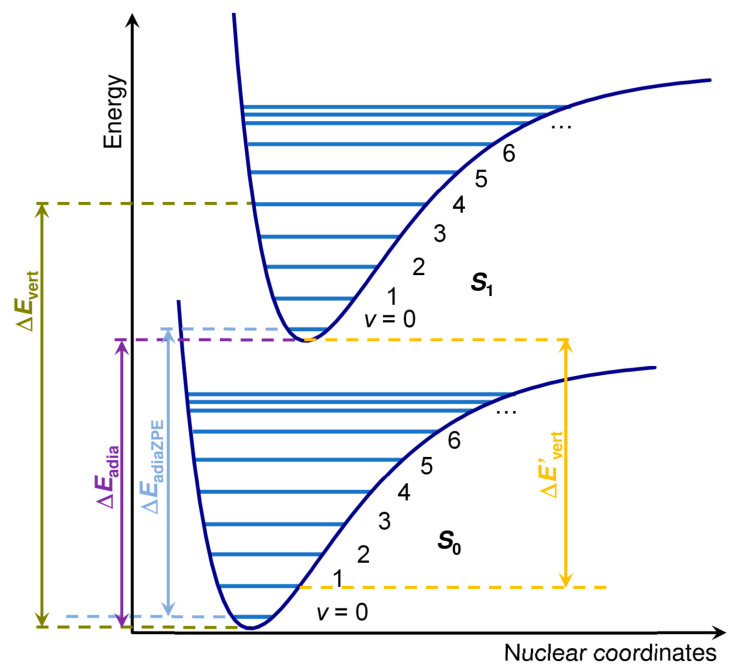
Graphical definition of the energies used in this work.

The vertical excitation energies (Δ*E*_vert_) and purely electronic adiabatic excitation energies (Δ*E*_adia_) for *S*_0_ → *S*_1_ were obtained as the energy difference between the ground state energy and the energy of the first excited state computed through single-point and geometry optimization, respectively. No difference was observed between geometry optimization, including the first 5 and 100 excitations in an initial assessment using ωB97X-D. For this reason, only the first five excitations were considered in the geometry optimization. ZPVE-corrected vertical adiabatic excitation energies were also obtained (Δ*E*_adiaZPVE_). A graphical definition of these energies is reported in Figure 8. The corresponding values for the *S*_1_ → *S*_0_ transition are indicated with a prime (see Figure 8).

The radiative decay rates $k_{\mathrm{rad}}$ of the S_1_ species can be estimated as the Einstein coefficient for the spontaneous emission, as defined in Refs. [50,51]:

(4) $$A_{S1\to S0}=k_{\mathrm{rad}}=\frac{{\left({\Delta E}_{\mathrm{vert}}^{}\right)}^{3}\cdot {\left|\mu_{S0S1}\right|}^{2}}{3\cdot \pi \cdot \varepsilon_{0}\cdot \hbar^{4}\cdot c^{3}}$$

where $\varepsilon_{0}$, c, and ħ represent the vacuum permittivity, the speed of light, and the reduced Planck constant, respectively, while $\mu_{S0\to S1}$ is the ground to excited state transition electric dipole moment.

## 4. Conclusions

In this article, we report on the synthesis of three biocompatible biindole diketonates and their BF_2_ compounds. Difluoroboronation is confirmed to be an effective strategy to increase, by as much as one order of magnitude, the fluorescence quantum yield of organic compounds, presenting a suitable chelating group [10,27,52,53] and, in particular, for diketonates [6,10]. The above features make these BF_2_bdks interesting for low-energy-consumption lighting devices, such as organic light-emitting diodes (OLEDs [54]) and fluorescent sensors. Chemical modification of the indole does not significantly influence the electronic spectroscopy of these BF_2_bdks.

These experimental observations were qualitatively reproduced by all the (TD)DFT functionals used. Although TD-B3LYP-D3 showed the best agreement with the experimental excitation energies among the levels used (overestimation of ~50 nm), this method was determined to be unreliable for the determination of the excited-state geometries, for which other methods (e.g., ωB97X-D) should be preferred.

The new BF_2_bdks presented in this article combine their biocompatibility with fluorescence properties and the indole moiety, which is notably bioactive. We show that the stabilization of the *S*_1_ excited state towards deactivation in BF_2_bdks is large enough to enable sizeable ^1^O_2_ photosensitized production. The above figures of merit promise future perspectives of these compounds for applications in biology and medicinal chemistry.

## Figures and Tables

**Table 1:** Fractional concentrations (**f_diketoNMR_**) of diketo conformers of **3a**, **3b**, and **3c** diluted in different solvents, based on ^1^H NMR.

Solvent	3a	3b	3c
toluene-*d*_8_	0.98	0.93	0.98
dichloromethane-*d*_2_	0.98	0.85	0.88
chloroform-*d*_3_	0.96	0.95	0.91
acetone-*d*_6_	0.59	0.68	0.77
dimethyl sulfoxide-*d*_6_	0.71	0.79	0.96

**Table 2:** UV-Vis absorption properties of **4a**, **4b**, and **4c** (see Figure 1). Maximum absorption wavelength (λ_abs_) and molar extinction coefficient at λ_abs_ (ε).

Compound	Solvent	λ_abs_ (nm)	ε (M^−1^ cm^−1^)
**4a**	toluene	431	77,992
	ethyl acetate	431	80,466
	dichloromethane	438	78,551
	acetone	438	92,290
	acetonitrile	440	38,198
	dimethylformamide	444	70,958
	dimethyl sulfoxide	448	114,365
	butanol	438	71,339
	ethanol	438	71,339
	methanol	438	45,636
**4b**	toluene	428	54,183
	ethyl acetate	429	73,733
	dichloromethane	435	75,012
	acetone	435	58,993
	acetonitrile	437	74,380
	dimethylformamide	441	83,860
	dimethyl sulfoxide	446	76,780
	butanol	436	45,249
	ethanol	435	54,870
	methanol	435	52,060
**4c**	toluene	436	62,211
	ethyl acetate	436	63,106
	dichloromethane	443	61,339
	acetone	442	68,664
	acetonitrile	444	72,734
	dimethylformamide	448	72,235
	dimethyl sulfoxide	452	77,678
	butanol	443	43,209
	ethanol	442	52,377
	methanol	442	69,881

**Table 3:** UV-Vis absorption properties of **3a**, **3b**, and **3c**. Peak absorption wavelengths in each of the three absorption bands resolved within the electronic transition spectra: λ_abs1,diketo_, λ_abs2,diketo_, and λ_abs,enol_. f_diketo_: fractional concentrations of *trans*-diketo (see text for details on calculation) ^1^.

Compound	Solvent	λ_abs1,diketo_ (nm)	λ_abs2,diketo_ (nm)	λ_abs,enol_ (nm)	f_diketo_
**3a**	toluene	-	309	387, 372	0.81
	ethyl acetate	-	303	384	0.75
	dichloromethane	247	308	388	0.88
	acetone	-	-	386	-
	acetonitrile	245	303	387	0.78
	dimethylformamide	-	298	391	0.83
	dimethyl sulfoxide	-	301	394	0.85
	butanol	248	309	386	0.90
	ethanol	246	308	386	0.91
	methanol	246	308	388	0.91
**3b**	toluene	-	312	382	0.85
	ethyl acetate	251	304	382	0.76
	dichloromethane	250	305	382	0.75
	acetone	-	-	382	-
	acetonitrile	260	301	382	0.85
	dimethylformamide	-	308	386	0.77
	dimethyl sulfoxide	-	281	388	0.96
	butanol	253	312	383	0.88
	ethanol	250	307	382	0.85
	methanol	251	307	382	0.89
**3c**	toluene	-	313	392	0.88
	ethyl acetate	255	304	388	0.81
	dichloromethane	257	308	396	0.89
	acetone	-	-	394	-
	acetonitrile	255	302	392	0.89
	dimethylformamide	-	298	392	0.85
	dimethyl sulfoxide	-	301	398	0.92
	butanol	254	312	398	0.91
	ethanol	254	311	390	0.92
	methanol	254	309	398	0.93

^1^ In acetone, the solvent absorbance becomes overwhelming below 330 nm, and we could only observe the enol peak. Similarly, in toluene, the solvent absorbance becomes overwhelming below 285 nm and in dimethylformamide and dimethyl sulfoxide below 270 nm.

**Table 4:** Fluorescence properties of **4a**, **4b**, and **4c**. Maximum emission wavelength, λ_fluo_, fluorescence quantum yield, ϕ_fluo_, fluorescence lifetime, τ, radiative and non-radiative rate constants, *k*_Rad_ and *k*_NR_.

Compound	Solvent	λ_fluo_ (nm)	ϕ_fluo_	τ (ps)	*k*_Rad_ (s^−1^)	*k*_NR_ (s^−1^)
**4a**	toluene	447	0.31	1889 ± 4	1.6 × 10^8^	3.7 × 10^8^
	ethyl acetate	452	0.54	2035 ± 2	2.7 × 10^8^	2.3 × 10^8^
	dichloromethane	465	0.60	2082 ± 3	2.9 × 10^8^	1.9 × 10^8^
	acetone	460	0.50	2153 ± 9	2.3 × 10^8^	2.3 × 10^8^
	acetonitrile	467	0.45	2232 ± 3	2.0 × 10^8^	2.5 × 10^8^
	dimethylformamide	468	0.72	1989 ± 1	3.6 × 10^8^	1.4 × 10^8^
	dimethyl sulfoxide	475	0.66	2125 ± 1	3.1 × 10^8^	1.6 × 10^8^
	butanol	466	0.22	2189 ± 4	1.0 × 10^8^	3.6 × 10^8^
	ethanol	466	0.17	2104 ± 4	8.1 × 10^7^	3.9 × 10^8^
	methanol	469	0.33	2139 ± 4	1.5 × 10^8^	3.1 × 10^8^
**4b**	toluene	448	0.59	1893 ± 2	3.1 × 10^8^	2.2 × 10^8^
	ethyl acetate	450	0.52	2117 ± 1	2.5 × 10^8^	2.3 × 10^8^
	dichloromethane	461	0.55	2198 ± 4	2.5 × 10^8^	2.0 × 10^8^
	acetone	455	0.62	2038 ± 2	3.0 × 10^8^	1.9 × 10^8^
	acetonitrile	461	0.56	2126 ± 3	2.6 × 10^8^	2.1 × 10^8^
	dimethylformamide	463	0.61	2137 ± 2	2.9 × 10^8^	1.^8^ × 10^8^
	dimethyl sulfoxide	467	0.67	1931 ± 5	3.5 × 10^8^	1.7 × 10^8^
	butanol	462	0.54	2084 ± 3	2.6 × 10^8^	2.2 × 10^8^
	ethanol	461	0.61	2226 ± 1	2.6 × 10^8^	1.8 × 10^8^
	methanol	462	0.56	2142 ± 1	2.6 × 10^8^	2.1 × 10^8^
**4c**	toluene	456	0.50	2165 ± 3	2.3 × 10^8^	2.3 × 10^8^
	ethyl acetate	461	0.46	2330 ± 1	2.0 × 10^8^	2.3 × 10^8^
	dichloromethane	477	0.47	2623 ± 4	1.8 × 10^8^	2.0 × 10^8^
	acetone	468	0.44	2237 ± 2	2.0 × 10^8^	2.5 × 10^8^
	acetonitrile	473	0.17	1121 ± 3	1.5 × 10^8^	7.4 × 10^8^
	dimethylformamide	476	0.39	1905 ± 1	2.0 × 10^8^	3.2 × 10^8^
	dimethyl sulfoxide	481	0.39	1684 ± 1	2.3 × 10^8^	3.6 × 10^8^
	butanol	474	0.42	1828 ± 5	2.3 × 10^8^	3.2 × 10^8^
	ethanol	475	0.28	1397 ± 25	2.0 × 10^8^	5.2 × 10^8^
	methanol	480	0.07	519 ± 3	1.3 × 10^8^	1.8 × 10^8^

**Table 5:** Fluorescence properties of **3a**, **3b**, and **3c** measured upon excitation at the enol absorption band. Peak emission wavelength, λ_fluo_, fluorescence quantum yield, ϕ_fluo_, average fluorescence lifetime, τ_Ave_, radiative and non-radiative rate constants, *k*_Rad_ and *k*_NR_.

Compound	Solvent	λ_fluo_ (nm)	ϕ_fluo_	τ_Ave_ (ps)	*k*_Rad_ (s^−1^)	*k*_NR_ (s^−1^)
**3a**	toluene	437	0.004	577	6.9 × 10^6^	1.7 × 10^9^
	ethyl acetate	411, 451	0.023	1802	1.3 × 10^7^	5.4 × 10^8^
	dichloromethane	422	0.020	409	4.9 × 10^7^	2.4 × 10^9^
	acetone	440	0.011	980	1.1 × 10^7^	1.0 × 10^9^
	acetonitrile	421	0.011	325	3.4 × 10^7^	3.0 × 10^9^
	dimethylformamide	419, 464	0.055	1521	3.6 × 10^7^	6.2 × 10^8^
	dimethyl sulfoxide	426	0.040	448	8.9 × 10^7^	2.1 × 10^9^
	butanol	440	0.059	409	1.4 × 10^8^	2.3 × 10^9^
	ethanol	440	0.069	369	1.9 × 10^8^	2.5 × 10^9^
	methanol	444	0.093	448	2.1 × 10^8^	2.0 × 10^9^
**3b**	toluene	433	0.009	463	1.9 × 10^7^	2.1 × 10^9^
	ethyl acetate	409, 430	0.006	172	3.5 × 10^7^	5.8 × 10^9^
	dichloromethane	435, 443	0.056	491	1.1 × 10^8^	1.9 × 10^9^
	acetone	414, 428	0.033	156	2.1 × 10^8^	6.2 × 10^9^
	acetonitrile	418, 433	0.013	230	5.6 × 10^7^	4.3 × 10^9^
	dimethylformamide	423, 441	0.011	232	4.8 × 10^7^	4.3 × 10^9^
	dimethyl sulfoxide	421, 435	0.044	311	1.4 × 10^8^	3.1 × 10^9^
	butanol	435, 441	0.037	427	8.7 × 10^7^	2.3 × 10^9^
	ethanol	437	0.033	277	1.2 × 10^8^	3.5 × 10^9^
	methanol	438	0.049	449	1.1 × 10^8^	2.1 × 10^9^
**3c**	toluene	416, 450	0.022	448	4.9 × 10^7^	2.2 × 10^9^
	ethyl acetate	414, 438	0.011	187	5.9 × 10^7^	5.3 × 10^9^
	dichloromethane	441	0.024	300	8.0 × 10^7^	3.2 × 10^9^
	acetone	421, 438	0.033	147	2.2 × 10^8^	6.6 × 10^9^
	acetonitrile	425, 444	0.025	354	7.1 × 10^7^	2.8 × 10^9^
	dimethylformamide	425, 449	0.061	755	8.1 × 10^7^	1.2 × 10^9^
	dimethyl sulfoxide	429, 446	0.072	400	1.8 × 10^8^	2.3 × 10^9^
	butanol	450	0.103	474	2.2 × 10^8^	1.9 × 10^9^
	ethanol	447, 450	0.122	644	1.9 × 10^8^	1.4 × 10^9^
	methanol	450	0.119	674	1.8 × 10^8^	1.3 × 10^9^

**Table 6:** Singlet oxygen quantum yields, ϕ(^1^O_2_), of the bdk and BF_2_bdk compounds dissolved in ethanol relative to TSPP(aq).

Compound	ϕ(^1^O_2_)
TSPP	0.620 *
**3a**	0.018
**3b**	0 **
**3c**	0 **
**4a**	0.389
**4b**	0.074
**4c**	0.066

* From Ref. [28]. ** The fluorescence of 9,10-dimethylanthracene is quenched less than in the absence of putative photosensitizers after irradiation with the Wood lamp.

**Table 7:** Excited state properties of **3a** keto-enol, **3a** *trans*-diketo, **4a**, **4b**, and **4c**. Vertical (Δ*E*_vert_), adiabatic (Δ*E*_adia_), and ZPVE-corrected adiabatic (Δ*E*_adiaZPVE_) excitation energies for the *S*_0_ → *S*_1_ transition in the gas phase. The HOMO to LUMO energy difference in *S*_0_ (Δ*E*_HOMO-LUMO_) and the vertical de-excitation energy for *S*_1_ → *S*_0_ transition ($\Delta E_{\mathrm{vert}}^{\prime }$) are also showed for comparison. All the energies are reported in eV. The absorption wavelength (λ_absB3_, nm) corresponding to the *S*_0_ → *S*_1_ transition is also showed with its oscillator strength (*f*_absB3_, a.u.), ground to excited state transition electric dipole moment (${}_{S0\to S1}$ a.u.), and the radiative decay rates of the *S*_1_ state (*k*_radB3_, s^−1^), defined as in Equation (4). *k*_radB3_ is computed considering the intensity and the maximum of the corresponding band in the computed spectrum. The emission wavelength (λ_fluoB3_) corresponding to *S*_1_ → *S*_0_ computed on *S*_1_ geometry, and its oscillator strength (*f*_fluoB3_) is also reported. All the values have been obtained at DFT/TD-DFT with B3LYP-D3/def2-TZVPD.

	3a Keto-Enol	3a *trans*-Diketo ^1^	4a	4b	4c ^2^
Δ*E*_HOMO-LUMO_	3.84	4.41	3.58	3.61	3.49
$\Delta E_{\mathrm{vert}}^{}$	3.42	3.74	3.21	3.21	3.06
Δ*E*_adia_	3.30	3.52	3.13	3.13	3.01
Δ*E*_adiaZPVE_	3.13	3.33	2.96	2.96	2.92
$\Delta E_{\mathrm{vert}}^{\prime }$	−3.13	−3.22	−3.05	−3.05	−2.93
$\mu_{S0\to S1}$	9.4	(0.9, 0.2, 1.5)	11.2	12.0	11.3
λ_absB3_	362.0	308.4 (331.34, 314.30, 314.28)	386.6	386.2	405.2
*f*_absB3_	0.79	(0.08, 0.02, 0.14)	0.88	0.94	0.85
*k*_radB3_	3.76 × 10^9^	1.41 × 10^8^	4.41 × 10^9^	5.05 × 10^9^	3.91 × 10^9^
λ_fluoB3_	396.6	327.8 (384.57, 331.87, 331.49)	406.0	407.1	422.9
*f*_fluoB3_	0.69	(0.03, 0.02, 0.22)	0.88	0.94	0.87

^1^ The lowest energy band in the absorption, emission, and excitation spectra of **3a** *trans*-diketo is the result of the convolution of more than one signal. The wavelength value is the maximum of the band resulting from the convolution of these signals, while the three lowest energy transitions contributing to this band are reported in parentheses. For **3a** *trans*-diketo, *k*_radB3_ is computed for the third transition, i.e., the one having the higher oscillator strength. ^2^ The first excited state values for **4c** have been obtained as single point on the CAM-B3LYP-D3 geometry. The ZPE has been assumed coincident with the one computed at the CAM-B3LYP-D3 level.

## Data Availability

Not applicable.

## Supplementary Materials

The following supporting information can be downloaded at: https://www.mdpi.com/article/10.3390/molecules28124688/s1, Figure S1. ATR-IR spectra recorded on loose powders in the air of 3a before (black curve) and after difluoroboronation (4a, yellow curve), 4b (pink), 4c (violet). Figure S2. Theoretical IR spectra computed at the B3LYP/def2-TZVPD in the gas phase for the three possible conformers of 3a: trans-diketo (black curve), cis-diketo (pink), keto-enol (violet). Figure S3. UV-Vis absorption spectra of HBIP (3a, top), BClIP (3b, middle) and BMIP (3c, below) in representative solvents. Figure S4. Electronic absorption spectra of (a) the three conformers of 3a and (b) the BF2bdks compounds as computed at the TDA-ωB97X-D/def2-TZVPD including the first 100 excitations. Figure S5. Electronic absorption spectra of (a) the three conformers of 3a and (b) the BF2bdks compounds as computed at the TDA-M06-2X/def2-TZVPD including the first 100 excitations. Figure S6. Electronic absorption spectra of (a) the three conformers of 3a and (b) the BF2bdks compounds as computed at the TDA-CAM-B3LYP-D3/def2-TZVPD including the first 100 excitations. Figure S7. Electronic absorption spectra of (a) the three conformers of 3a and (b) the BF2bdks compounds as computed at the TDA-B3LYP-D3/def2-TZVPD including the first 100 excitations. Figure S8. Electronic absorption spectra of (a) the three conformers of 3a and (b) the BF2bdks compounds as computed at the TD-ωB97X-D/def2-TZVPD including the first 100 excitations. Figure S9. The TCSPC apparatus instrumental response to <10 ps laser pulses at 420 nm (violet, laser pulse duration 2.8 ps), 532 nm (green, laser pulse duration 6.4 ps), and 1064 nm (dark red, laser pulse duration 9 ps). The full-width at half maximum of the temporal point-spread functions is in any case < 30 ps. Figure S10. Exemplary dataset for determination of molar extinction coefficient values (namely 4a in dimethyl sulfoxide). Figure S11. Global minimum geometry for the first excited state of 4c as obtained at the TD- and TDA-B3LYP-D3/def2-TZVPD. Figure S12. Electronic fluorescence spectra of (a) the three conformers of 3a and (b) the BF2bdks compounds as computed at the TDA-ωB97X-D/def2-TZVPD for S1 including the first 100 excitations. Figure S13. Electronic fluorescence spectra of (a) the three conformers of 3a and (b) the BF2bdks compounds as computed at the TD-ωB97X-D/def2-TZVPD for S1 including the first 100 excitations. Figure S14. Electronic fluorescence spectra of (a) the three conformers of 3a and (b) the BF2bdks compounds as computed at the TDA-M06-2X/def2-TZVPD for S1 including the first 100 excitations. Figure S15. Electronic fluorescence spectra of (a) the three conformers of 3a and (b) the BF2bdks compounds as computed at the TDA-CAM-B3LYP-D3/def2-TZVPD for S1 including the first 100 excitations. Figure S16. Electronic fluorescence spectra of (a) the three conformers of 3a and (b) the BF2bdks compounds as computed at the TDA-B3LYP-D3/def2-TZVPD for S1 including the first 100 excitations. The spectra are obtained on the TDA-CAM-B3LYP-D3 geometry for S1. Table S1. Carbonyl stretching frequency as measured by ATR-IR in the air of the as-synthetized bdks (3) and BF2bdks (4). Table S2. Fluorescence decay times and relative amplitudes, fi, of compounds 3a, 3b, and 3c, as retrieved from fitting the experimental decay patterns measured upon excitation at 280 nm to a biexponential decay model function. Table S3. Fluorescence decay times and relative amplitudes, fi, of compounds 3a, 3b, and 3c, as retrieved from fitting the experimental decay patterns measured upon excitation at 420 nm to a biexponential decay model function. Table S4. Excited state properties of 3a keto-enol, 3a trans-diketo, 4a, 4b, and 4c as computed at DFT/TDA-DFT with ωB97X-D/def2-TZVPD. Table S5. Excited state properties of 3a keto-enol, 3a trans-diketo, 4a, 4b, and 4c as computed at DFT/TD-DFT with ωB97X-D/def2-TZVPD. Table S6. Excited state properties of 3a keto-enol, 3a trans-diketo, 4a, 4b, and 4c as computed at DFT/TDA-DFT with M06-2X/def2-TZVPD. Table S7. Excited state properties of 3a keto-enol, 3a trans-diketo, 4a, 4b, and 4c as computed at DFT/TDA-DFT with CAM-B3LYP-D3/def2-TZVPD. Table S8. Excited state properties of 3a keto-enol, 3a trans-diketo, 4a, 4b, and 4c as computed at DFT/TDA-DFT with B3LYP-D3/def2-TZVPD.

## Sample Availability

Samples of the compounds are available from the authors upon request.

## Appendix A

The DFT-optimized structure of the three conformers of HBIP (**3a**) is reported in Figure A1: *trans*-diketo, *cis*-diketo, and keto-enol. Several input structures were used for the three conformers, for which optimization resulted in one of the three conformers reported in Figure A1. Only the keto-enol compound is planar, while the *cis-* and *trans*-diketo prefers a bent geometry, with an angle of ~110° between the rings. This difference is expected based on the different hybridization (sp^2^ and sp^3^, respectively) of the C atom connecting the C=O groups in the keto-enol and the diketo conformers, respectively. For the *cis*-diketo, the two C=O groups are rotated to maximize the distance of the two negatively charged oxygen atoms, i.e., to minimize their repulsive interaction.

The relative energy stability of the three conformers of **3a** in the gas phase is listed in Table A1, as obtained using the different functionals here employed. All the levels of approximation provide coincident results and agree in indicating the *trans*-diketo as the most stable conformer. Its energy is lower by about 10 kJ mol^−1^ than the keto-enol and 25 kJ mol^−1^ than *cis*-diketo. The relative stability of the conformers is slightly changed if the optimization is conducted by including toluene as implicit solvent. Based on a Boltzmann distribution, we can estimate at 25 °C a relative ratio among *trans*-diketo:keto-enol: *cis*-diketo of 94:6:0.

**Table A1:** Relative energy stability (in kJ mol^−1^) of the ground state of the three conformers of **3a** in the gas phase, as obtained using different DFT functionals. The values obtained by performing the optmization by considering an implicit solvent having the same dielectric constant of toluene (ε = 2.379) are also reported for comparison.

	*trans*-Diketo	*cis*-Diketo	Keto-Enol
B3LYP-D3	0.0	26.8	11.6
B3LYP-D3 (toluene)	0.0	19.7	10.8
M06-2X	0.0	- ^1^	9.4
CAM-B3LYP-D3	0.0	25.8	10.9
ωB97X-D	0.0	24.7	18.6

^1^ The optimization of the *cis* conformer was not possible at this level of calculations, always resulting in the corresponding *trans* conformer.

We also compared the relative energy stability of the **3a** conformers in the first excited state in Table A2. All the levels employed agree that **3a** *cis*-diketo is the most unstable conformer, as already discussed for the ground state. For what concerns the relative stability of **3a** *trans*-diketo and **3a** keto-enol, this value is both functional and TD/TDA-dependent, not allowing one to draw clear conclusions like for the ground state.

**Table A2:** Relative energy stability (in kJ mol^−1^) of the first excited state of the three conformers of **3a** in the gas phase, as obtained using different TDDFT functionals.

	*trans*-Diketo	*cis*-Diketo	Keto-Enol
TD-B3LYP-D3	9.5	38.6 ^1^	0.0
TDA-B3LYP-D3	0.0 ^1^	24.4 ^1^	1.7 ^1^
TDA-M06-2X	0.2	16.3	0.0
TDA-CAM-B3LYP-D3	3.0	21.1	0.0
TD-ωB97X-D	3.5	20.1	0.0
TDA-ωB97X-D	0.0	16.7	10.1

^1^ Single-point calculations on the CAM-B3LYP geometry.
