# Supplementary material for: Synthesis, Characterization, Fluorescence Properties, and DFT Modeling of Difluoroboron Biindolediketonates
Source: Molecules. 2023 Jun 10;28(12):4688. doi: 10.3390/molecules28124688 (PMC10305660; doi:10.3390/molecules28124688)
Supplement: Supplementary file 1 [file molecules-28-04688-s001.zip › 2023-Maspero-Molecules-SI.pdf]

Supplementary Materials

Synthesis, Characterization, Fluorescence Properties, and DFT Modeling of Difluoroboron Biindolediketonates

Angelo Maspero [1], Federico Vavassori [1], Luca Nardo [1], Guglielmo Vesco [1], Jenny G. Vitillo [1*], Andrea Penoni [1*]

[1]Department of Science and High Technology and INSTM, University of Insubria, Via Valleggio 9, 22100 Como, Italy.

*Correspondence: (J.G.V.) jg.vitillo@gmail.com, (A.P.) andrea.penoni@uninsubria.it

Summary

S1. Synthesis of starting materials 4

S1.1. 5-Chloro-N-methyl-1H-indole (2b) 4

S1.2. 5-Methoxy-N-methyl-1H-indole (2c) 4

S1.3. 1,3-Bis(1-methyl-1H-indol-3-yl)propane-1,3-dione HBIP (3a) 4

S1.4 1,3-Bis(5-chloro-1-methyl-1H-indol-3-yl)propane-1,3-dione HBClIP (3b) 4

S1.5 1,3-Bis(5-methoxy-1-methyl-1H-indol-3-yl)propane-1,3-dione HBMIP (3c) 5

S2. NMR spectroscopy data on 4a 6

S2.1. ^{1}H-NMR spectrum of BF2BIP (4a) 6

S2.2. ^{13}C-NMR spectrum of BF2BIP (4a) 7

S2.3. ^{19}F-NMR spectrum of BF2BIP (4a) 8

S2.4. ^{11}B-NMR spectrum of BF2BIP (4a) 9

S3. NMR spectroscopy data on 4b 10

S3.1. ^{1}H-NMR spectrum of BF2ClBIP (4b) 10

S3.2. ^{13}C-NMR spectrum of BF2ClBIP (4b) 11

S3.3. ^{19}F-NMR spectrum of BF2ClBIP (4b) 12

S3.4. ^{11}B-NMR spectrum of BF2ClBIP (4b) 13

S4. NMR spectroscopy data on 4c 14

S4.1. ^{1}H-NMR spectrum of BF2BMIP (4c) 14

S4.2. ^{13}C-NMR spectrum of BF2BMIP (4c) 15

S4.3. ^{19}F-NMR spectrum of BF2BMIP (4c) 16

S4.4. ^{11}B-NMR spectrum of BF2BMIP (4c) 17

S5. IR spectra 18

S6. Supplementary experimental UV-Vis absorption spectroscopy data 20

S7. Supplementary theoretical UV-Vis absorption spectra and data 21

S7.1. TD-B3LYP-D3: Excitation energies and oscillator strengths of the first excited states 23

3a keto-enol ... 23
3a cis-diketo ... 23
3a trans-diketo ... 23
4a ... 24
4b ... 24
4c ... 24
S7.2. TDA-B3LYP-D3: Excitation energies and oscillator strengths of the first excited states ... 24
3a keto-enol ... 24
3a cis-diketo ... 25
3a trans-diketo ... 25
4a ... 25
4b ... 26
4c ... 26
S7.3. TDA-ωB97X-D: Excitation energies and oscillator strengths of the first excited states ... 26
3a keto-enol ... 26
3a cis-diketo ... 27
3a trans-diketo ... 27
4a ... 28
4b ... 28
4c ... 28
S7.4. TDA-M06-2X: Excitation energies and oscillator strengths of the first excited states ... 29
3a keto-enol ... 29
3a cis-diketo ... 29
3a trans-diketo ... 30
4a ... 30
4b ... 30
4c ... 31
S7.5. TDA-CAM-B3LYP-D3: Excitation energies and oscillator strengths of the first excited states ... 31
3a keto-enol ... 31
3a cis-diketo ... 31
3a trans-diketo ... 32
4a ... 32
4b ... 33
4c ... 33
S8. Supplementary electronic-state transition spectroscopy data ... 38
S9. Supplementary theoretical emission data ... 42

S1. Synthesis of starting materials

S1.1. 5-Chloro-N-methyl-1H-indole (2b)

Under inert atmosphere 5-chloro-1H-indole (3 g, 20 mmol, 1 equiv.) is dissolved in dry THF (80 mL) and sodium hydride (1.2 g, 60% suspension in mineral oil, 1.5 equiv.) is slowly added at 0 °C. After being stirred at 0 °C for 1.5 h methyl iodide (1.62 mL, 26 mmol, 1.3 equiv.) is added. The mixture is then warmed to rt and stirred overnight. After cooling again to 0°C, the reaction is quenched with water (40 mL) and extracted with diethyl ether (3x50 mL); the combined organic layers are dried over Na_2SO_4, filtered and the solvent removed under reduced pressure. The resulting crude product is purified via column chromatography (n-Hexane/EtOAc 9:1, R_f = 0.56) to give 5-chloro-*N*-methyl-1H-indole (2.84 g, 85% yield). ^{1}H-NMR ($CDCl_3$) δ = 7.58 (d, *J* = 1.6 Hz, 1H, ArH), 7.26 - 7.15 (m, 2H, ArH), 7.06 (d, *J* = 3.2 Hz, 1H, ArH), 6.42 (d, *J* = 3.2 Hz, 1H, ArH), 3.27 (s, 3H, N*CH₃*).

S1.2. 5-Methoxy-N-methyl-1H-indole (2c)

Under inert atmosphere, 5-Methoxy-1*H*-indole (2 g, 13.6 mmol, 1 eq.) is dissolved in dry THF (60 mL) and sodium hydride (0.81 g, 60% suspension in mineral oil, 1.5 eq.) is slowly added 0 °C. After being stirred at 0 °C for 1.5 min, methyl iodide (1.1 mL, 17.7 mmol, 1.3 eq.) is added. The mixture is warmed to rt and stirred overnight. After cooling again to 0°C, the reaction is quenched with water (40 mL) and extracted with diethyl ether (3x50 mL); the combined organic layers are dried over Na_2SO_4, filtered and the solvent removed under reduced pressure. The resulting crude product is purified via column chromatography (n-Hexane/EtOAc 9:1) to give 5-methoxy-N-methyl-1*H*-indole (2.09 g, 94% yield). ^{1}H-NMR ($CDCl_3$) δ = 7.30 (d, 1H, *J* = 8.5 Hz, ArH), 7.13 (s, 1H, ArH), 7.05 (s, 1 H, ArH), 6.92 (d, 1H, *J* = 8.8 Hz, ArH), 6.43 (d, 1H, *J* = 1.0 Hz, ArH), 3.90 (s, 3H, NCH_3), 3.80 (s, 3H, OCH_3).

S1.3. 1,3-Bis(1-methyl-1H-indol-3-yl)propane-1,3-dione HBIP (3a)

Under inert atmosphere, a solution of malonyl dichloride (1 mL, 10 mmol) in dichloromethane (10 mL) is added dropwise to a stirred solution of 1-methyl-1H-indole (2.55 mL, 20 mmol) in dichloromethane (15 mL) at 0°C. The reaction is stirred for 2h at rt. The mixture is then added to 5% aqueous sodium carbonate, vigorously stirred for 2 min and extracted with dichloromethane, dried over sodium sulfate and evaporated. The residue is purified via column chromatography (DCM/EtOAc 95:5) to obtain HBIP as a yellow powder (1.42 g, 42% yield). ^{1}H-NMR (d_6-DMSO) δ = 17.4 (keto-enol, br s, 1H, enol O*H*) 8.44 (diketo, s, 2H), 8.40 (keto-enol, s, 2H), 8.23 (keto-enol, d, ^3J = 7.6 Hz, 2H), 8.16 (diketo, d, ^3J = 7.6 Hz, 2H), 7.56 (diketo/keto-enol, m, 4H), 7.29 (diketo/keto-enol, m, 4H), 7.23 (diketo/keto-enol, m, 4H), 6.77 (keto-enol, s, 1H, enol C*H*), 4.39 (diketo, s, 2H, diketo CH_2), 3.91 (keto-enol, s, 6H, N-CH_3) 3.89 (diketo, s, 6H, N-CH_3). IR (ATR) ũ (cm^{-1}) = 3105 (w), 1618 (vs), 1575 (w), 1524 (vs), 1487 (vw), 1461 (s), 1421 (vw), 1391 (vw), 1365 (vs), 1334 (w), 1218 (s), 1126 (m), 1089 (s), 1081 (s), 1056 (w), 919 (s), 855 (w), 762 (w), 746 (s), 683 (w).

S1.4 1,3-Bis(5-chloro-1-methyl-1H-indol-3-yl)propane-1,3-dione HBClIP (3b)

Under inert atmosphere a solution of malonyl dichloride (973 µL, 10 mmol, 1 eq.) in dichloromethane (10 mL) is added dropwise to a stirred solution of 5-chloro-1-methyl-1H-indole (3.31 g, 20 mmol, 2 eq.) in dichloromethane (20 mL) at 0°C. The reaction is stirred for 2h at rt and then added to a 5% aqueous sodium carbonate solution, vigorously stirred for 2 min and extracted with dichloromethane, dried over Na_2SO_4 and evaporated. The residue is purified via column chromatography (DCM/n-Hexane 9:1) to obtain HBClIP as a yellow powder (0.911 g, 23% yield). ^{1}H-NMR (d_6-DMSO) δ = 8.5 (diketo, s, 2H, H2), 8.47 (keto-enol, s, 2H, H2) 8.19 (keto-enol, d, 2H, ^4J = 1.8 Hz, H4), 8.14 (diketo, d, 2H, ^4J = 1.5 Hz, H4), 7.63 (keto-enol, d, 2H, ^3J = 8.4 Hz) 7.61 (diketo, d, 2H, ^3J = 8.7 Hz, H7), 7.32 (diketo/keto-enol dd, ^3J = 8.8 Hz , ^4J = 1.6 Hz, H6, 4H), 6.77 (keto-enol, s, 1H, enol C*H*), 4.4 (diketo s, 2H, diketo CH_2), 3.91 (keto-enol, s, 6H, NCH_3), 3.90 (diketo, s, 6H, NCH_3). IR (ATR) ũ (cm^{-1}) = 3105 (vw); 2928 (vw); 2894 (vw); 1645 (m); 1631 (s); 1614 (m); 1572 (w); 1528 (vs); 1471 (sh, m); 1461 (s), 1452 (s), 1421 (w), 1405 (vw), 1385 (w), 1363 (vs), 1341 (sh, w), 1311 (vw), 1289 (vw), 1271 (w), 1235 (m), 1234 (m), 1217 (s), 1176 (w), 1159 (w), 1137 (s), 1089 (vs), 1058 (m), 1044 (m), 972 (w), 955 (w), 916 (vw), 888 (w), 883 (w), 856 (vw), 844 (w), 837, (w), 820 (m), 802 (s), 776 (m), 752 (w), 747 (w), 730 (s).

S1.5 1,3-Bis(5-methoxy-1-methyl-1H-indol-3-yl)propane-1,3-dione HBMIP (3c)

Under inert atmosphere a solution of malonyl dichloride (700 µL, 7.17 mmol, 1 eq.) in dichloromethane (8 mL) is added dropwise to a stirred solution of 5-methoxy-1-methyl-1H-indole (2.31 g, 14.3 mmol, 2 eq.) in dichloromethane (15 mL) at 0°C. The reaction is then stirred for 2h at rt and then added to a 5% aqueous sodium carbonate solution, vigorously stirred for 2 min and extracted with dichloromethane, dried over Na_2SO_4 and evaporated. The residue is purified via column chromatography (toluene/EtOAc 7:3) to obtain HBMIP (1.26 g, 45% yield) as a yellow solid. ^{1}H-NMR (d_6-DMSO) δ = 8.36 (diketo, s, 2H, H2) 7.67 (diketo d, ^4J = 2.1 Hz, 2H, H4), 7.45 (diketo, d, ^3J = 9 Hz, 2H, H7), 6.91 (diketo, dd, ^3J = 9 Hz, ^4J = 2.3 Hz, 2H, H6), 4.34 (diketo, s, 2H, diketo CH_2), 3.85 (diketo, s, 6H), 3.77 (diketo, s, 6H). IR (ATR) (cm^{-1}) = 2941 (w), 1713 (w), 1619 (vs), 1521 (s), 1477 (s), 1453 (vs), 1361 (vs), 1264 (s), 1222 (s), 1210 (s), 1181 (m), 1139 (m), 1131 (s), 1083 (s), 1021 (s), 912 (m), 873 (s), 853 (s), 835 (s), 799 (vs), 778 (m), 683 (m), 654 (vs), 640 (s).

S2. NMR spectroscopy data on 4a

S2.1. ^{1}H-NMR spectrum of BF2BIP (4a)

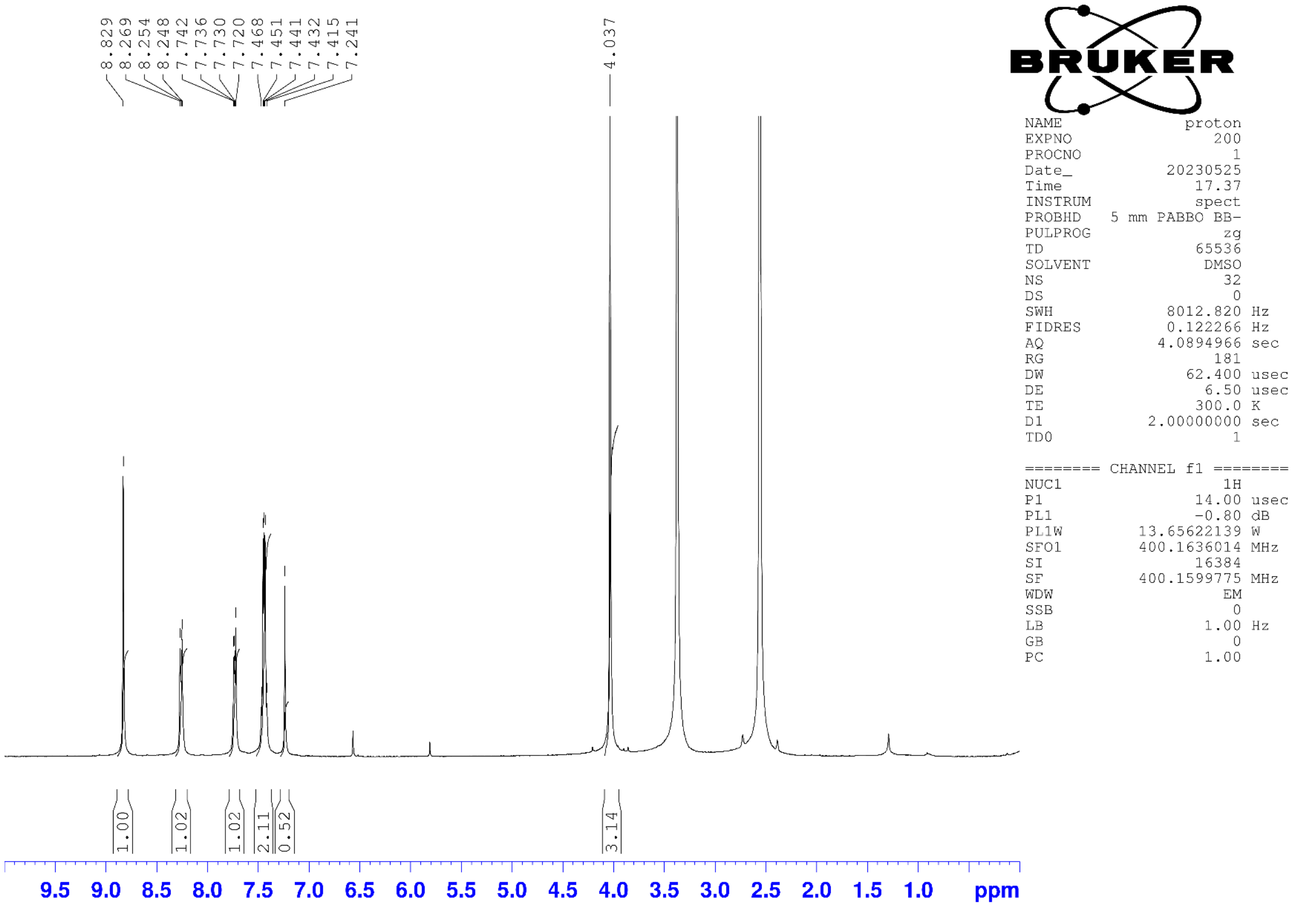

S2.2. ^{13}C-NMR spectrum of BF2BIP (4a)

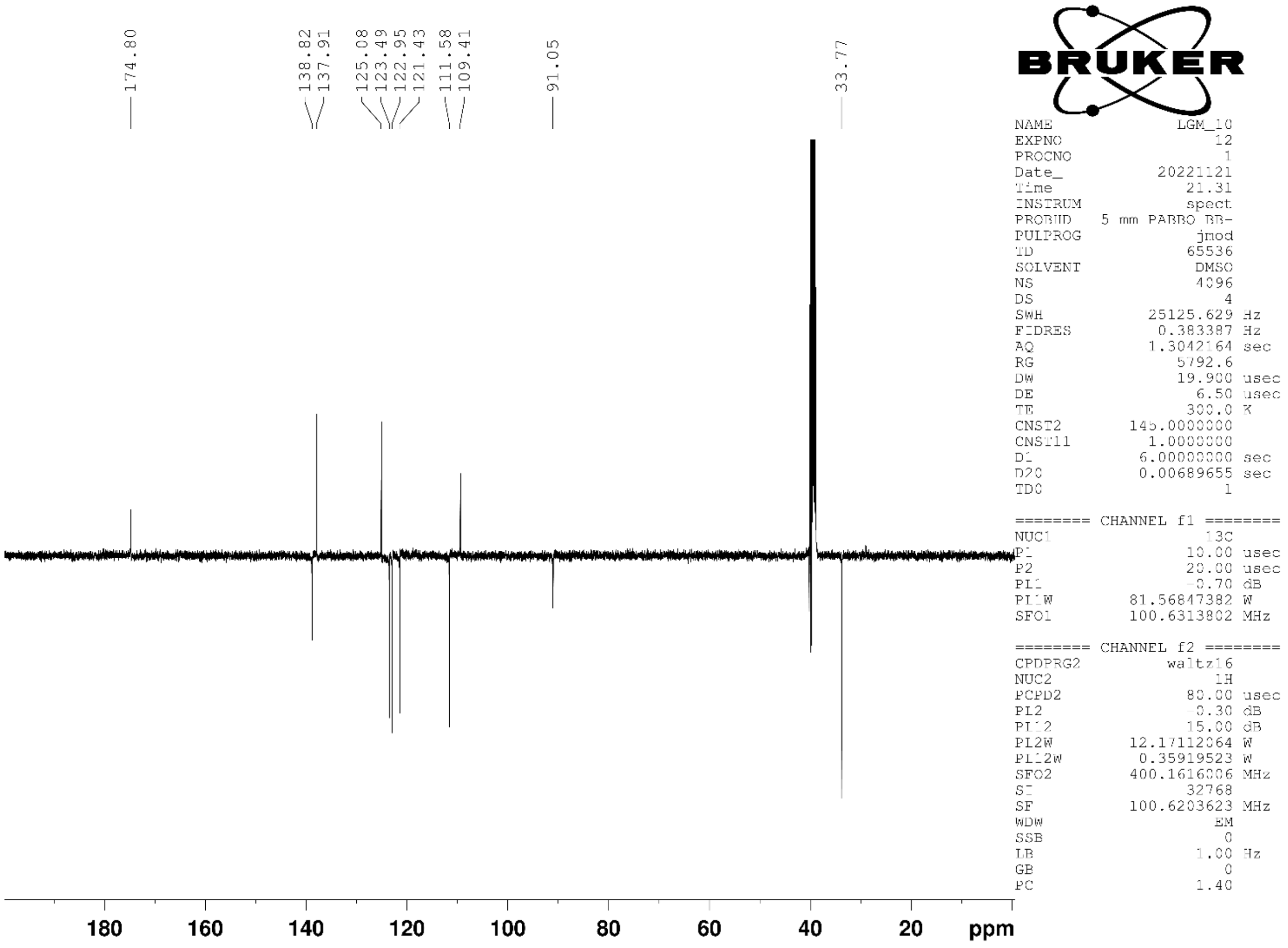

S2.3. ^{19}F-NMR spectrum of BF2BIP (4a)

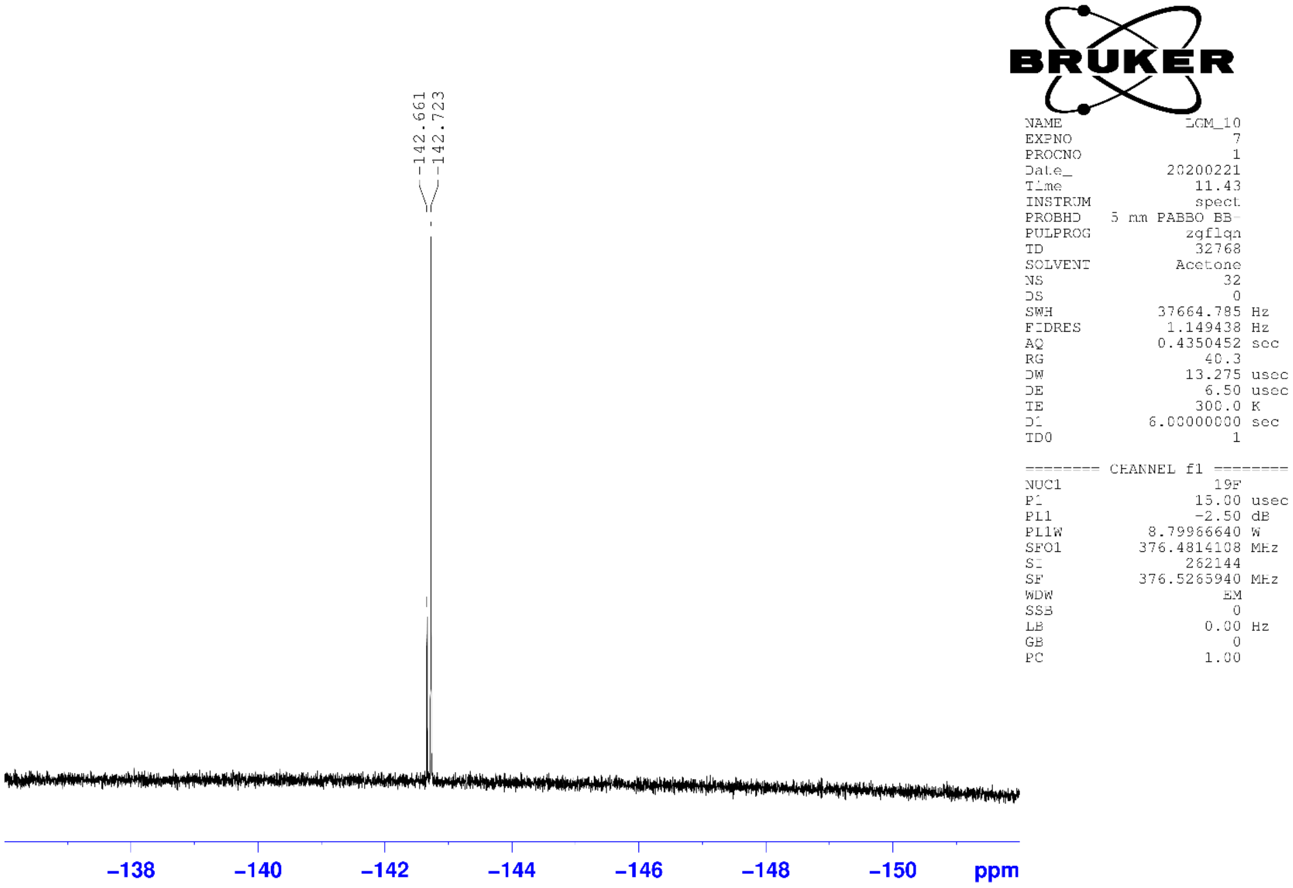

S2.4. ^{11}B-NMR spectrum of BF2BIP (4a)

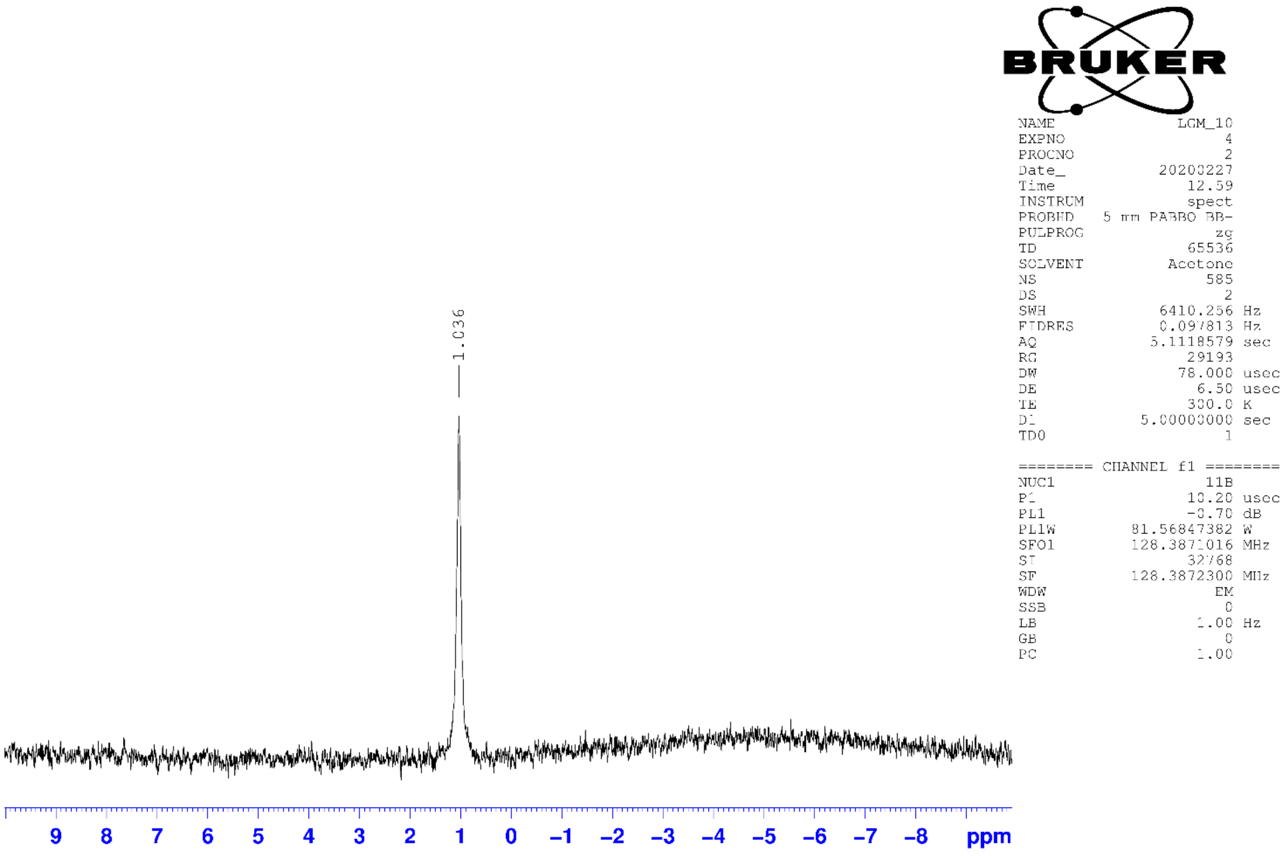

S3. NMR spectroscopy data on 4b

S3.1. ^{1}H-NMR spectrum of BF2ClBIP (4b)

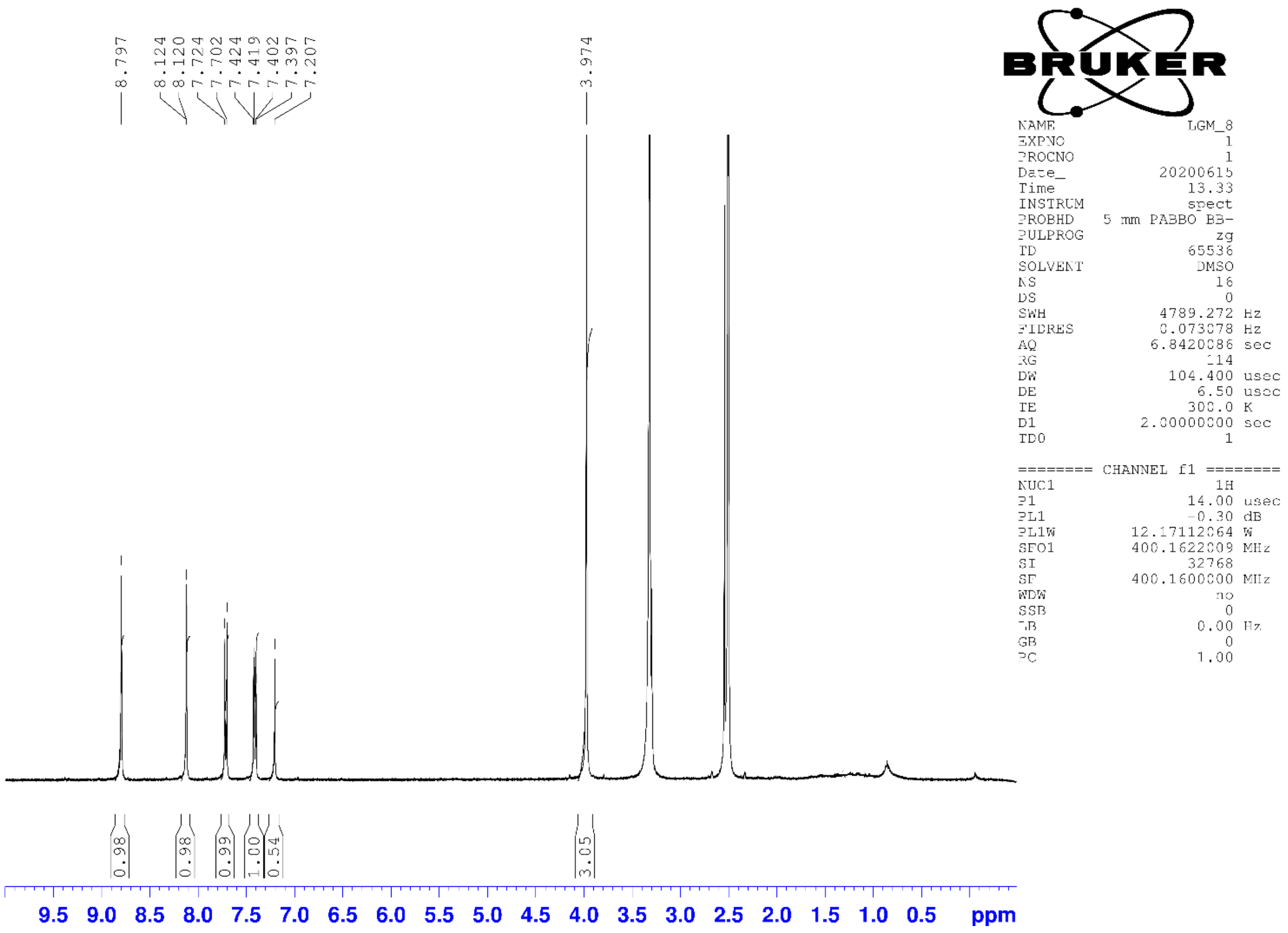

S3.2. ^{13}C-NMR spectrum of BF2ClBIP (4b)

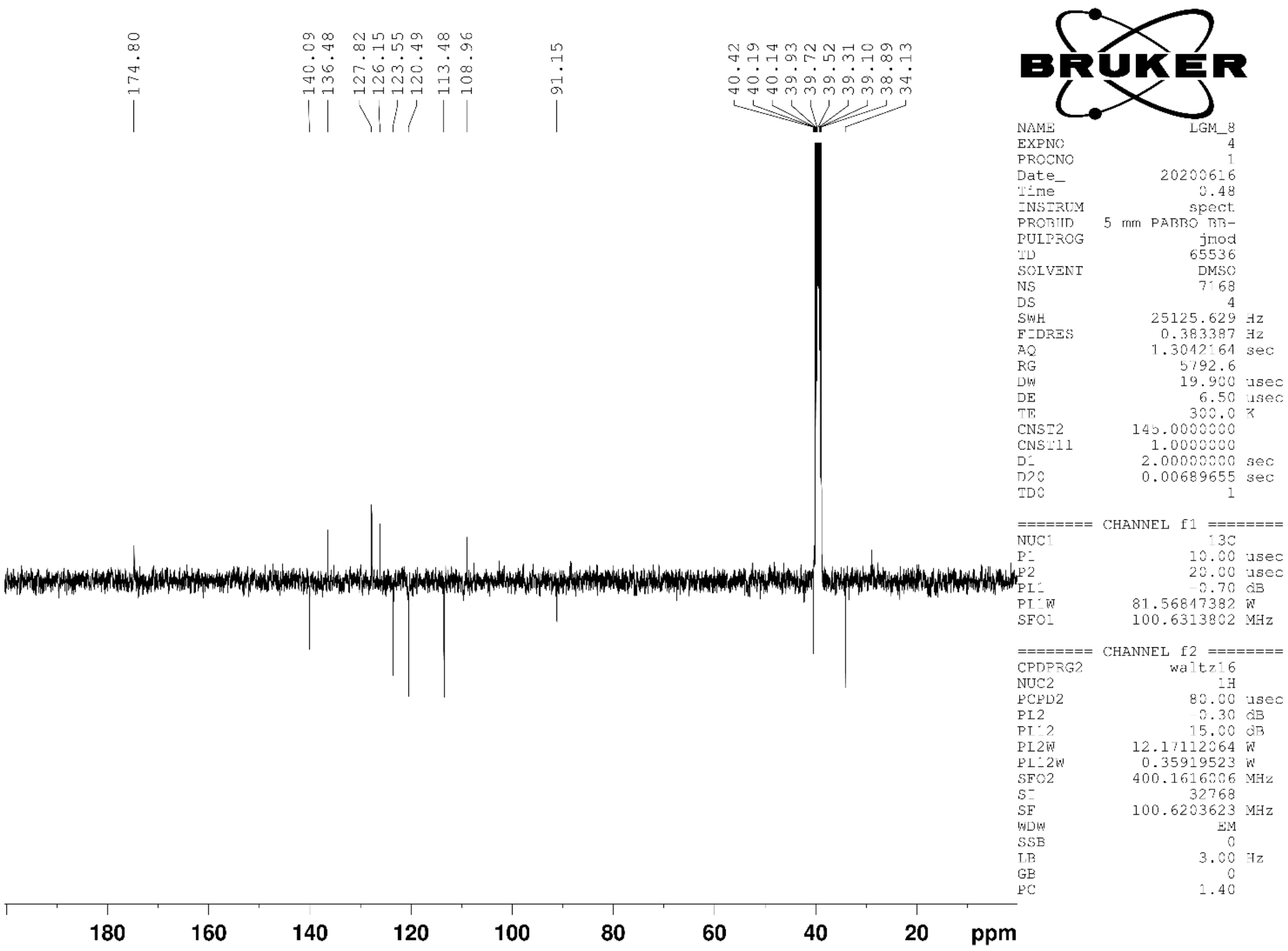

S3.3. ^{19}F-NMR spectrum of BF2ClBIP (4b)

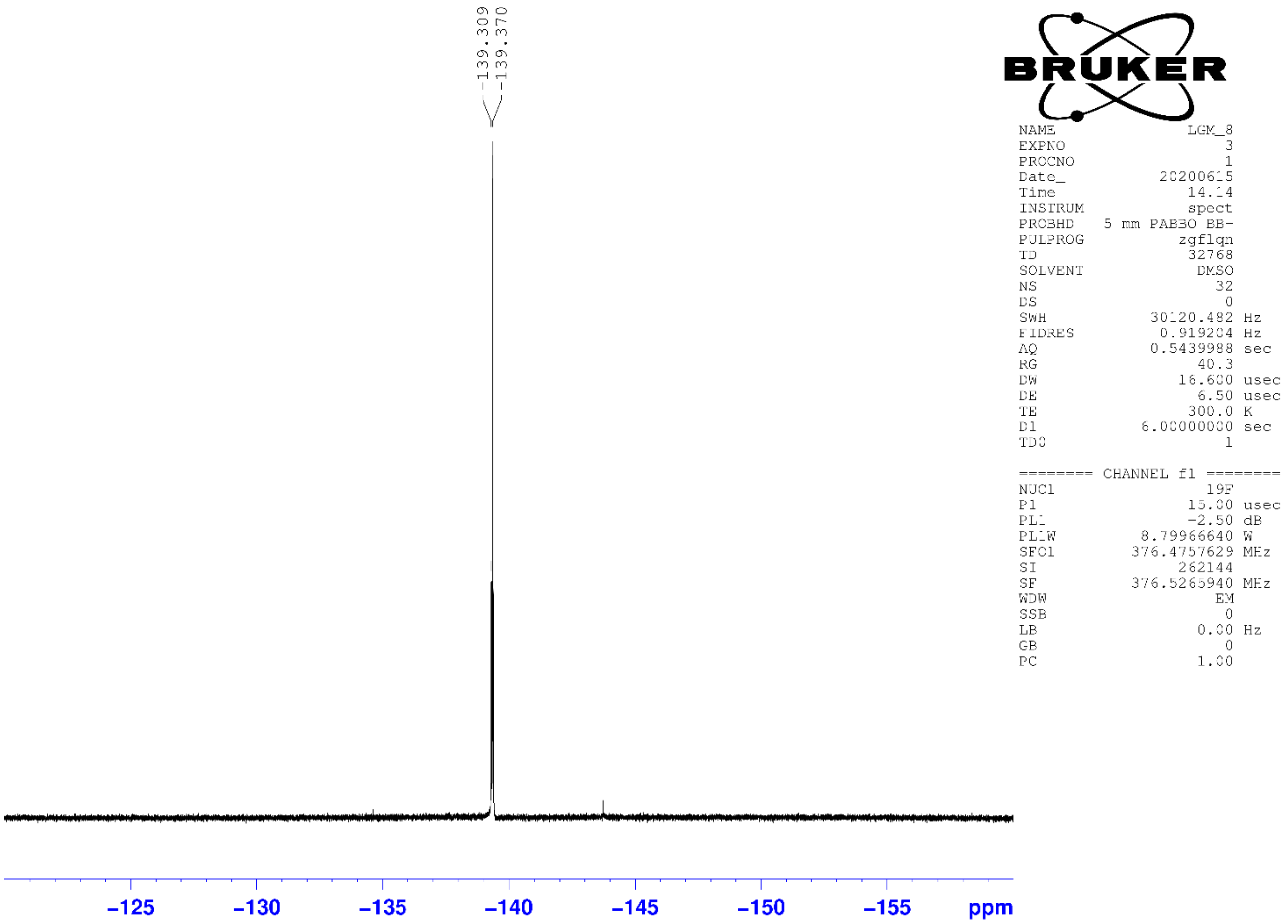

S3.4. ^{11}B-NMR spectrum of BF2ClBIP (4b)

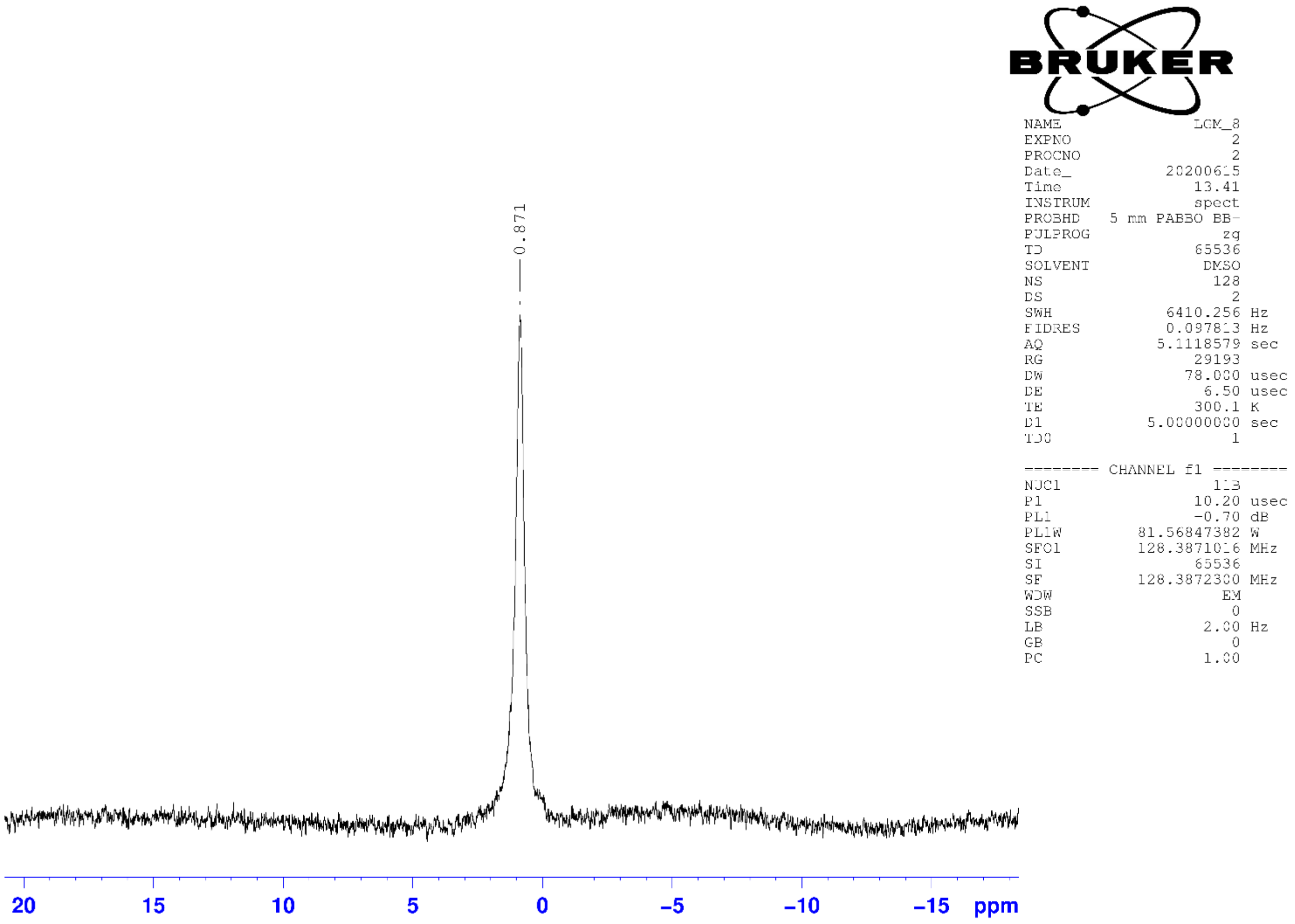

S4. NMR spectroscopy data on 4c

S4.1. ^{1}H-NMR spectrum of BF2BMIP (4c)

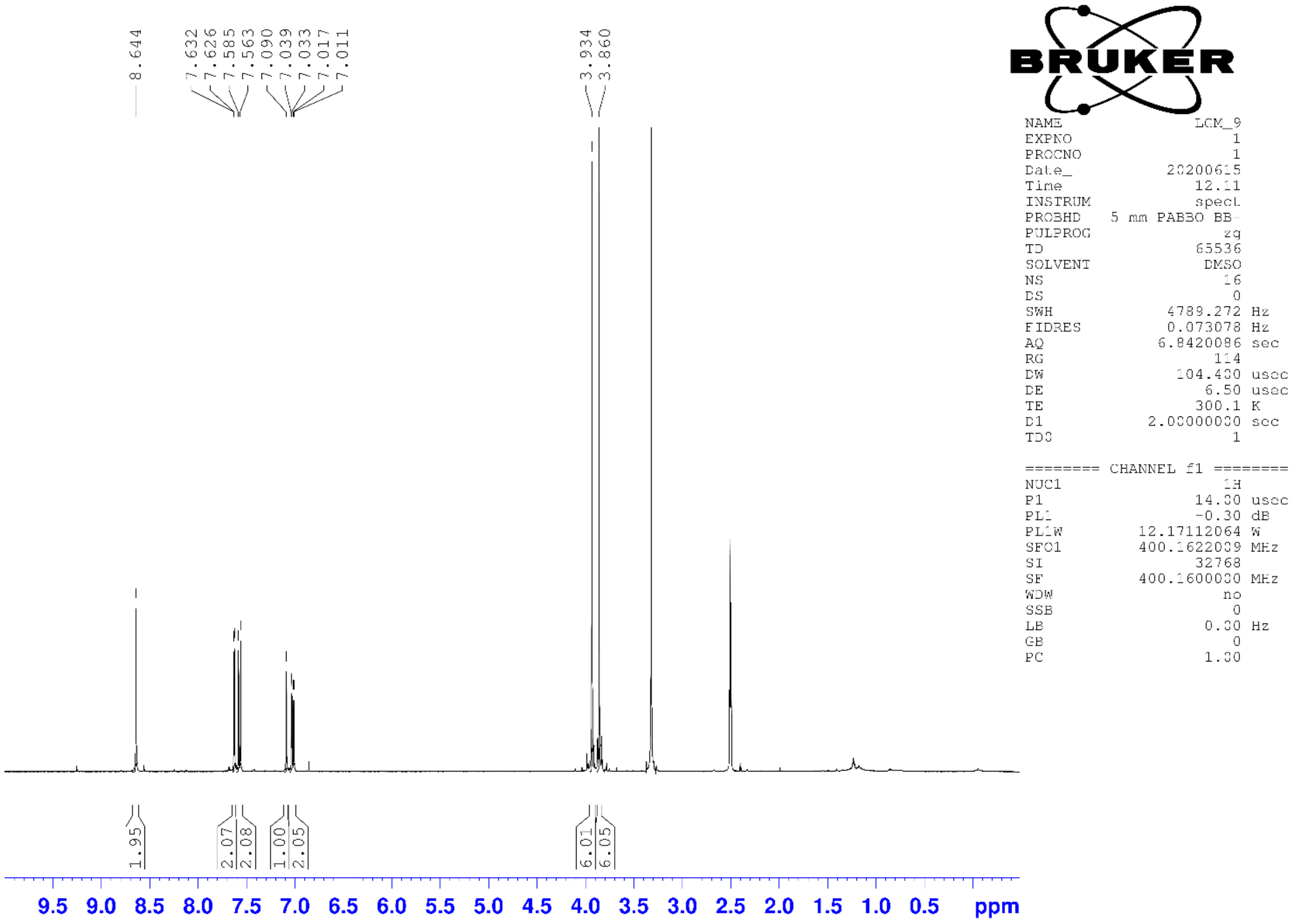

S4.2. ^{13}C-NMR spectrum of BF2BMIP (4c)

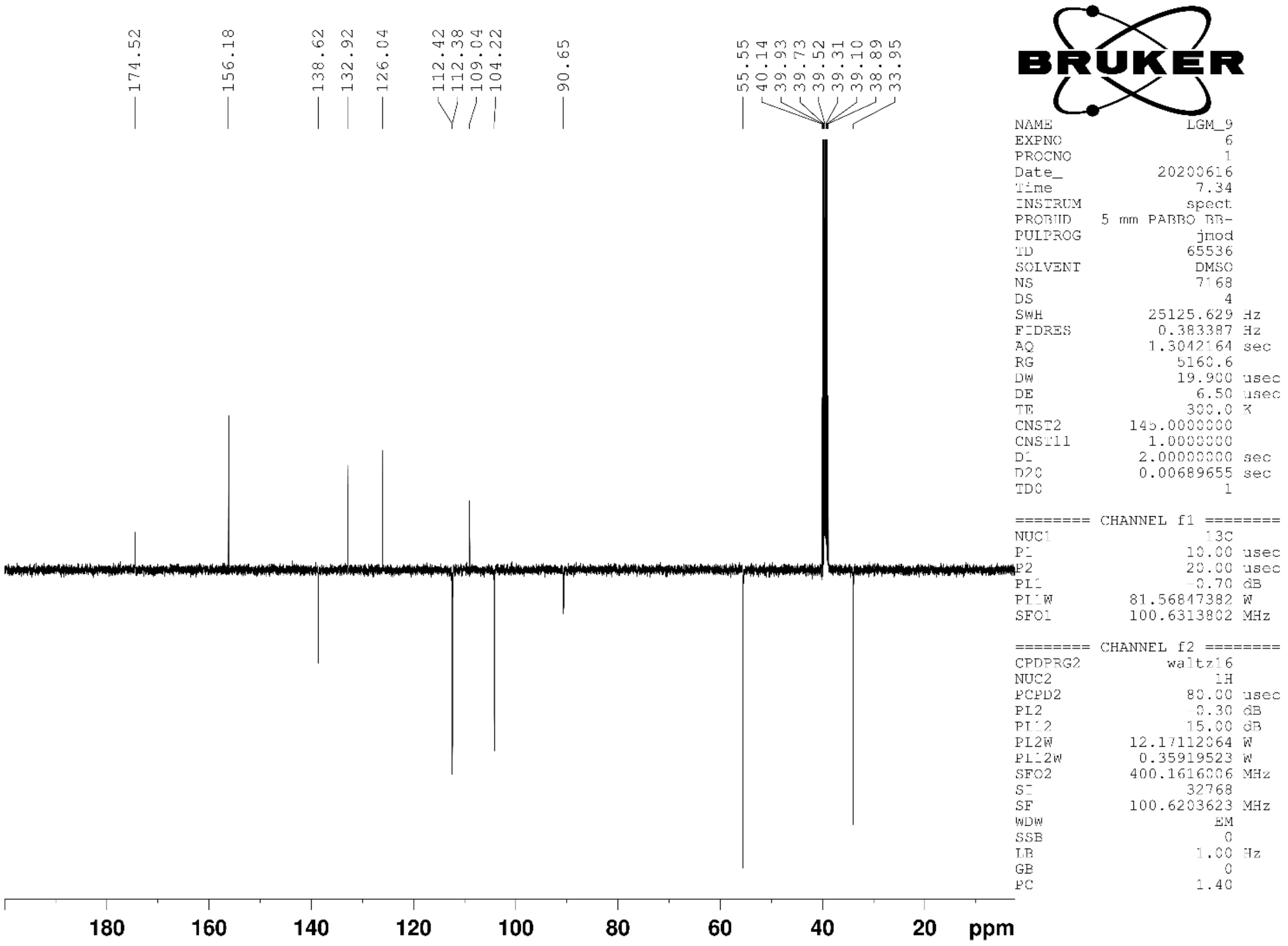

S4.3. ^{19}F-NMR spectrum of BF2BMIP (4c)

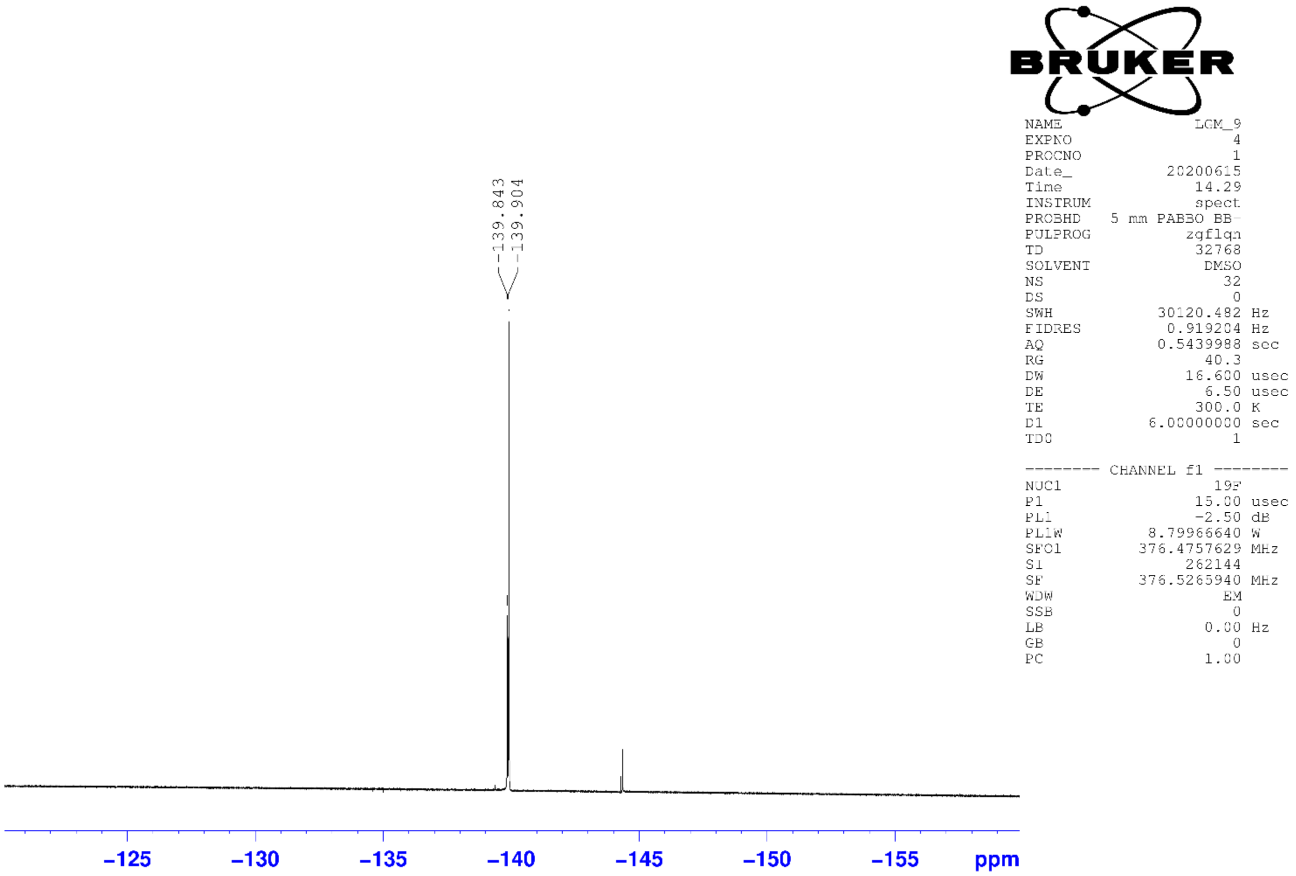

S4.4. ^{11}B-NMR spectrum of BF2BMIP (4c)

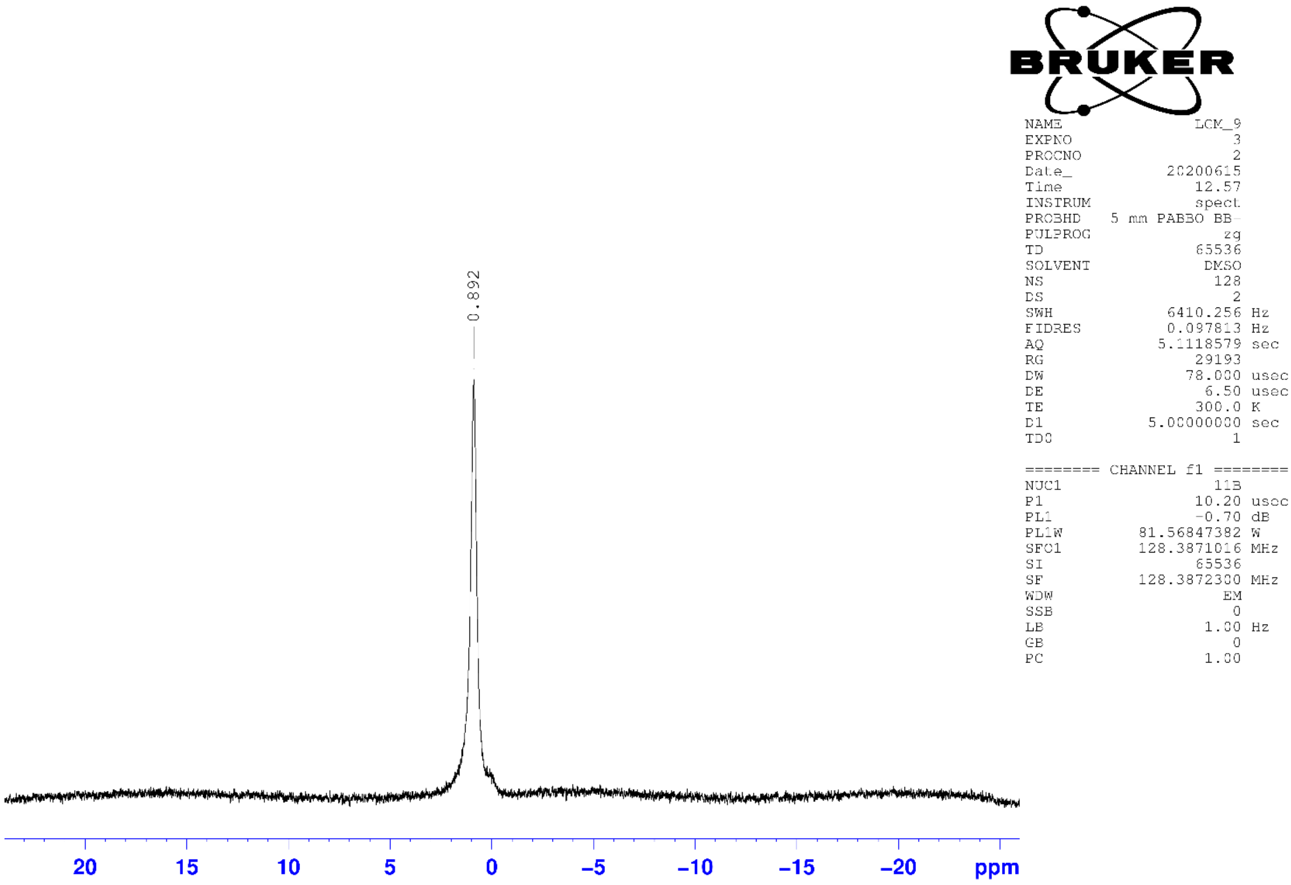

S5. IR spectra

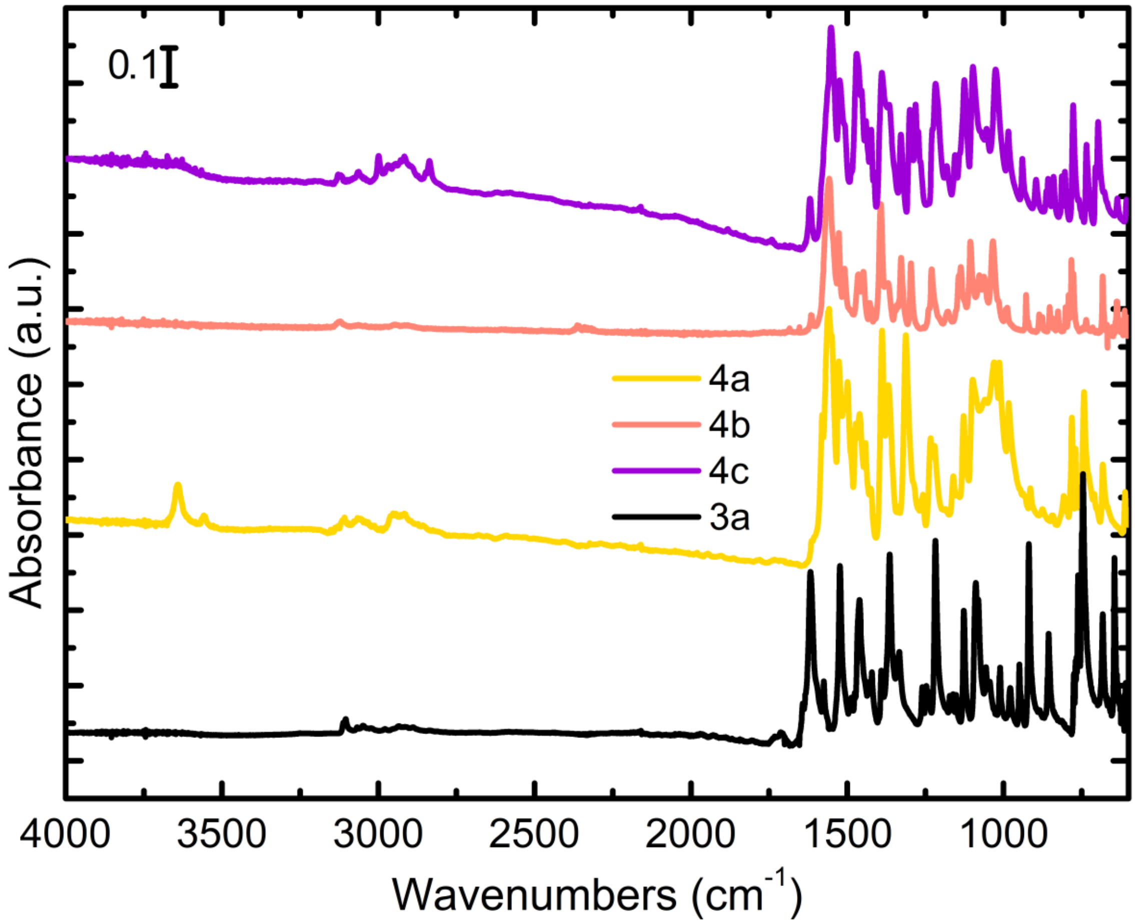

Figure S1. ATR-IR spectra recorded on loose powders in the air of **3a** before (black curve) and after difluoroboronation (**4a**, yellow curve), **4b** (pink), **4c** (violet). The peaks at ~3600 cm^{-1} in **4a** are associated to impurities (based on NMR spectra, boronic acids formed from the decomposition of BF_3).

Table S1. Carbonyl stretching frequency ($\tilde{\nu}_{C=O}$) as measured by ATR-IR in the air of the as-synthetized bdks (**3**) and BF_2bdks (**4**). The corresponding experimental shift of this frequency are also reported ($\Delta\tilde{\nu}_{C=O}$). All the values are in cm^{-1}.

Entry	$\tilde{\nu}_{C=O}$ **bdk 3**	$\tilde{\nu}_{C=O}$ **BF_2bdk 4**	$\Delta\tilde{\nu}_{C=O}$
**a**	1618	1560	-58
**b**	1630	1557	-73
**c**	1617	1555	-62

The computed DFT spectra for the three conformers of **3a** are reported in Figure S2. The calculations confirm the redshift of the carbonyl stretching mode after difluoroboronation (**4a**, yellow curve), although the computed $\Delta\tilde{\nu}_{C=O}$ are on average larger than the experimental values: -126 (trans-diketo, black curve), -163 (cis-diketo, pink), -42 cm^{-1} (keto-enol, violet) as computed at the B3LYP level. The difference between the computed and the experimental values is likely associated to the conditions used in the calculations (gas phase) and in the experiment (bulk phase).

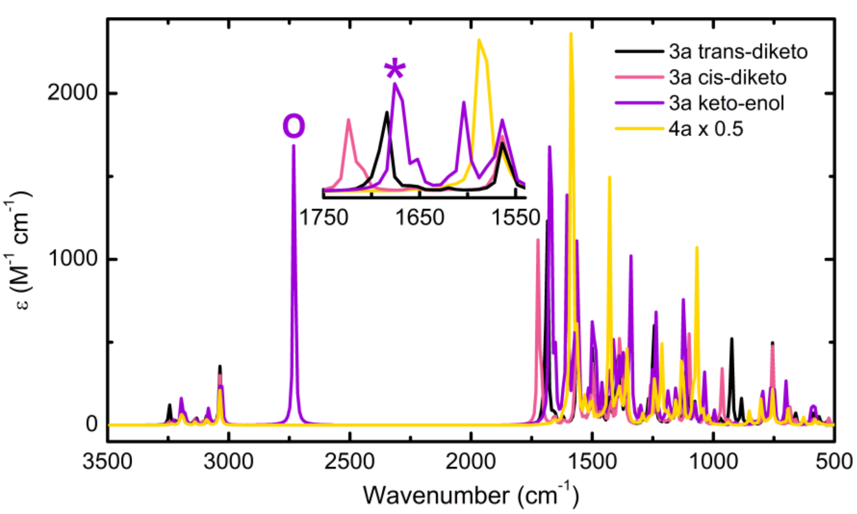

Figure S2. Theoretical IR spectra computed at the B3LYP/def2-TZVPD in the gas phase for the three possible conformers of **3a**: trans-diketo (black curve), cis-diketo (pink), keto-enol (violet). "O" and "*" mark the O-H stretching and bending modes in keto-enol, respectively. The spectrum of the corresponding BF_2bdk **4a** is also shown for comparison (yellow curve). Inset: enlarged view of the carbonyl stretching region.

S6. Supplementary experimental UV-Vis absorption spectroscopy data

The UV-Vis absorption spectra of **3a**, **3b**, and **3c** in different solvents are shown in Figure S3.

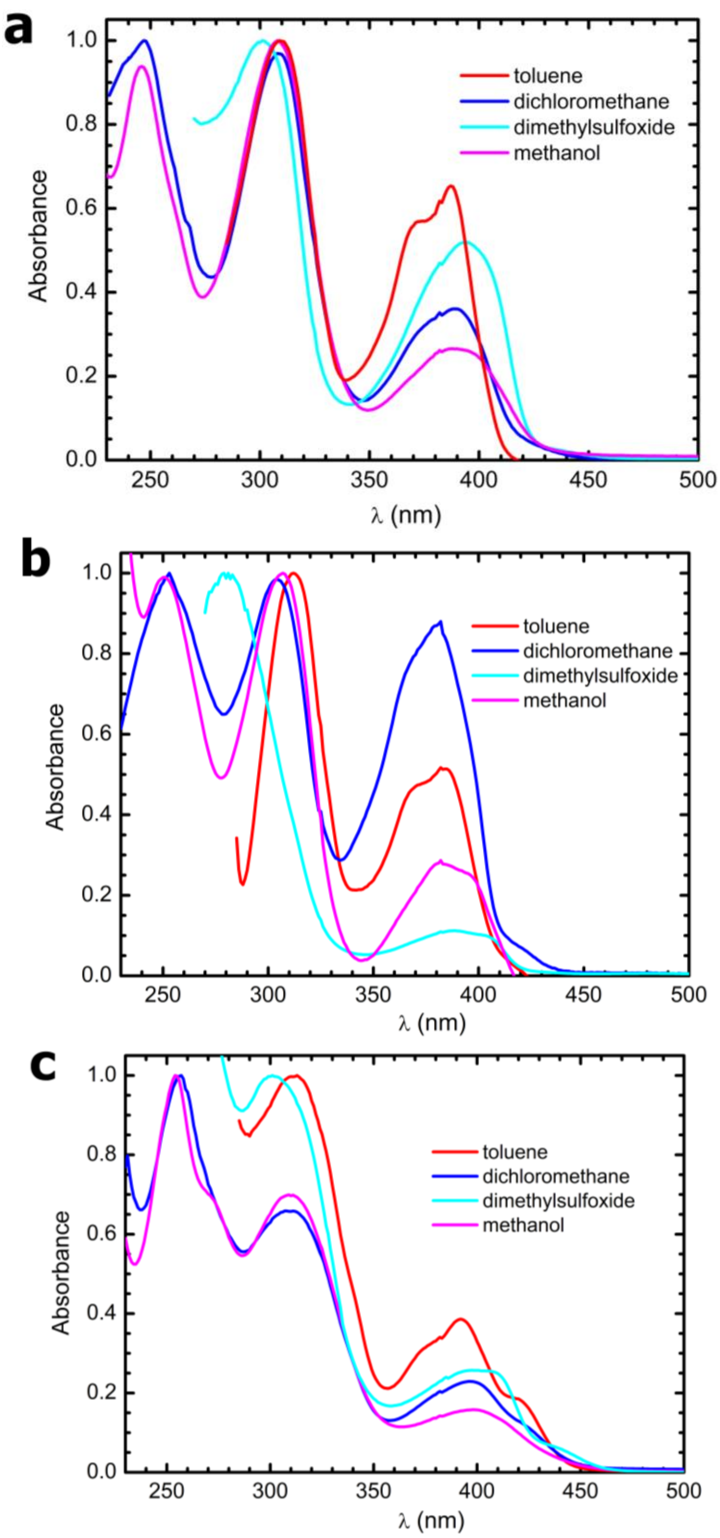

Figure S3. UV-Vis absorption spectra of HBIP (**3a**, top), BClIP (**3b**, middle) and BMIP (**3c**, below) in representative solvents.

S7. Supplementary theoretical UV-Vis absorption spectra and data

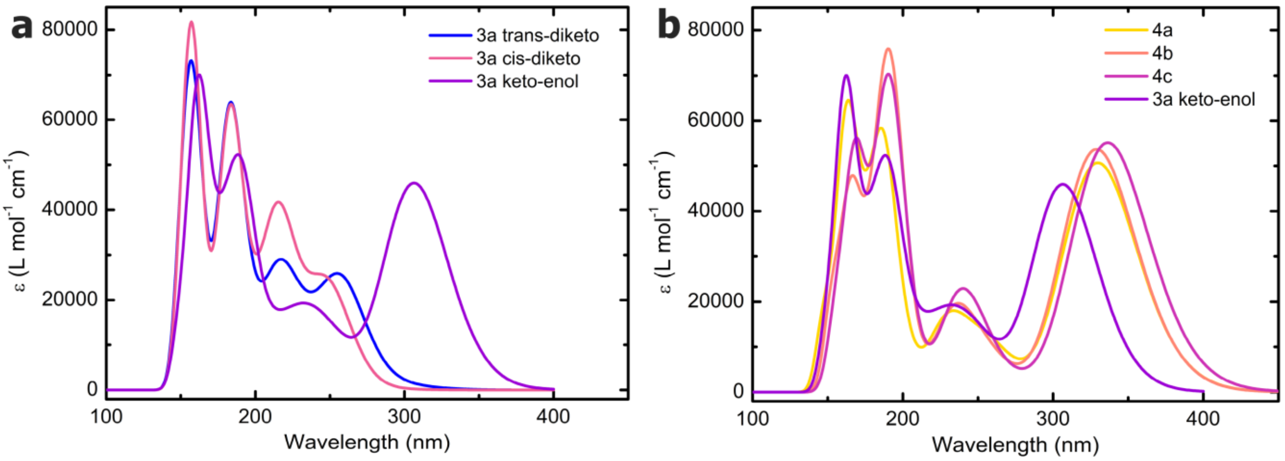

Figure S4. Electronic absorption spectra of (**a**) the three conformers of **3a** and (**b**) the BF_2bdks compounds as computed at the TDA-ωB97X-D/def2-TZVPD including the first 100 excitations.

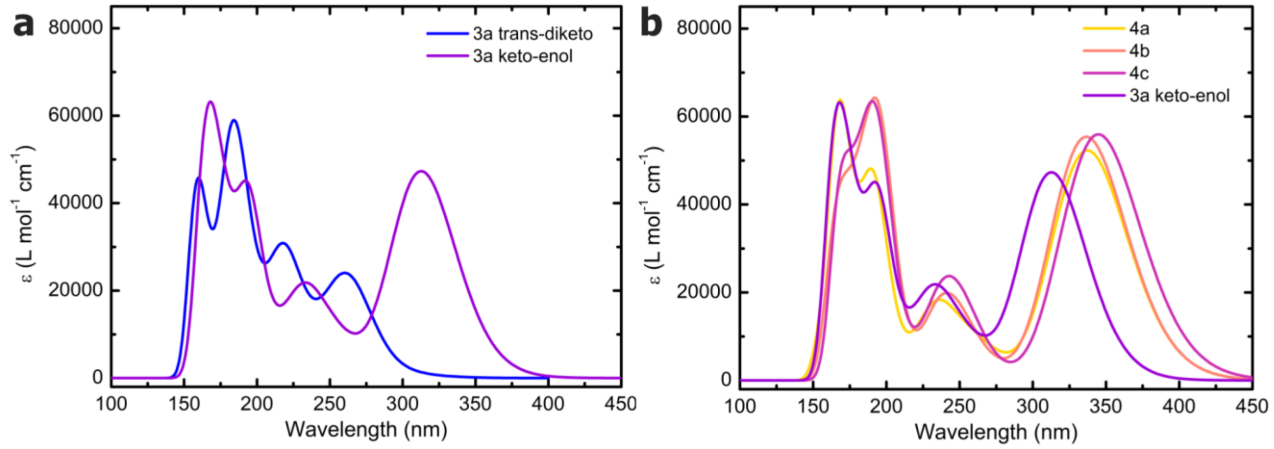

Figure S5. Electronic absorption spectra of (**a**) the three conformers of **3a** and (**b**) the BF_2bdks compounds as computed at the TDA-M06-2X/def2-TZVPD including the first 100 excitations.

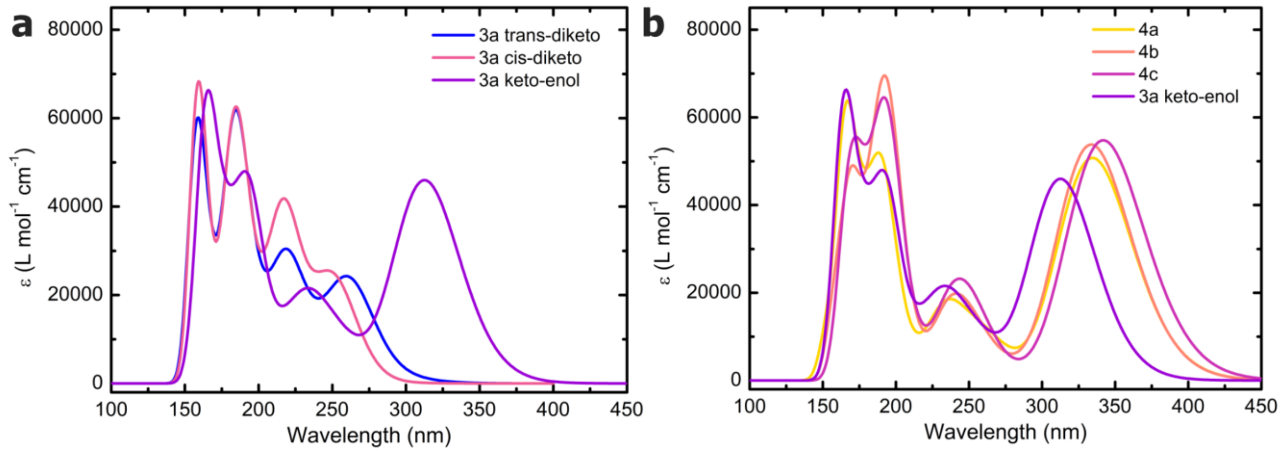

Figure S6. Electronic absorption spectra of (**a**) the three conformers of **3a** and (**b**) the BF_2bdks compounds as computed at the TDA-CAM-B3LYP-D3/def2-TZVPD including the first 100 excitations.

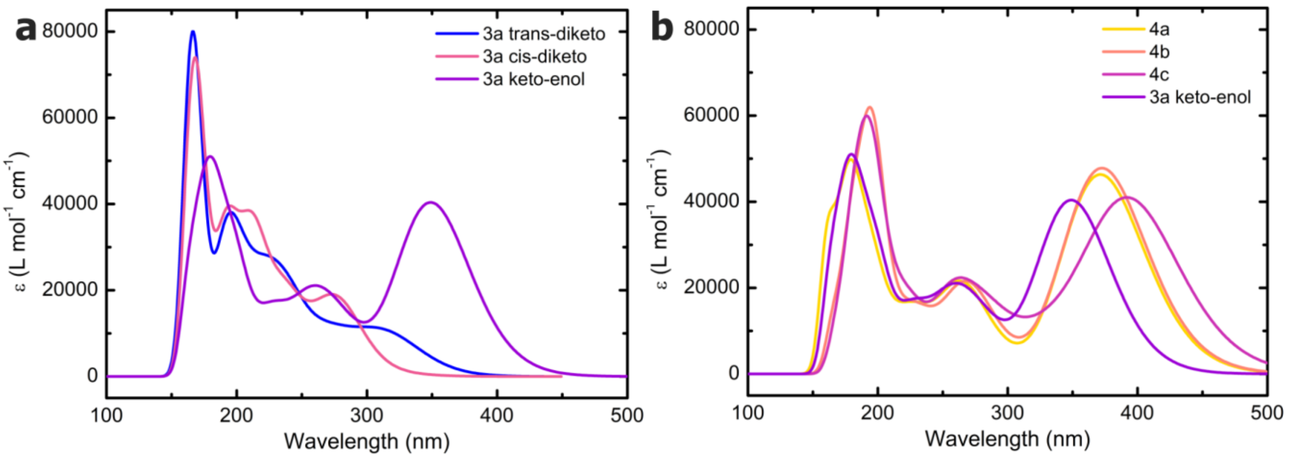

Figure S7. Electronic absorption spectra of (**a**) the three conformers of **3a** and (**b**) the BF_2bdks compounds as computed at the TDA-B3LYP-D3/def2-TZVPD including the first 100 excitations.

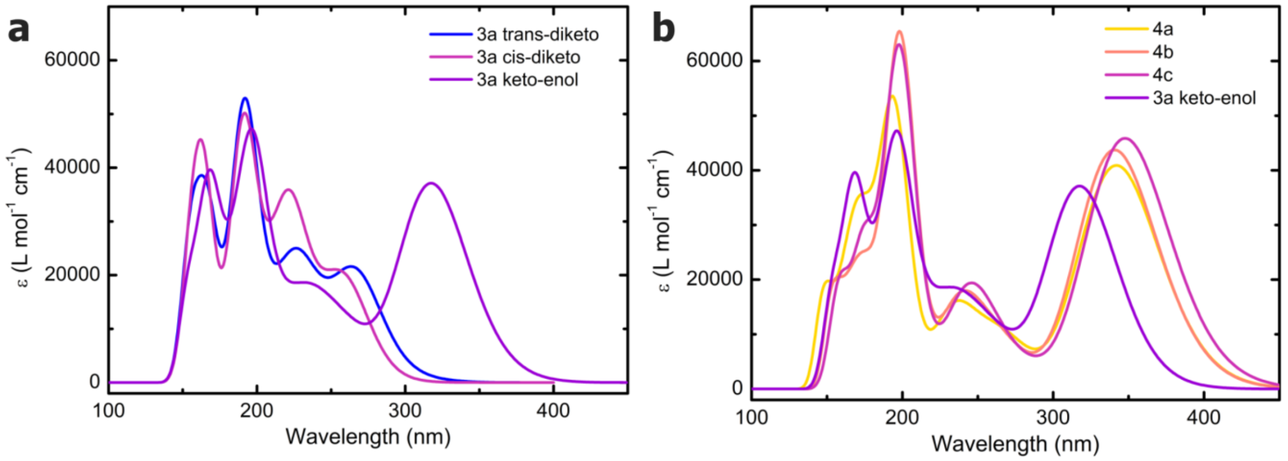

Figure S8. Electronic absorption spectra of (**a**) the three conformers of **3a** and (**b**) the BF_2bdks compounds as computed at the TD-ωB97X-D/def2-TZVPD including the first 100 excitations.

S7.1. TD-B3LYP-D3: Excitation energies and oscillator strengths of the first excited states

3a keto-enol

87 HOMO, 88 LUMO

```
Excited State   1:      Singlet-A      3.4247 eV  362.02 nm  f=0.7873  <S**2>=0.000
      87 -> 88         0.70215
 This state for optimization and/or second-order correction.
 Total Energy, E(TD-HF/TD-DFT) =  -1071.41850459
 Copying the excited state density for this state as the 1-particle RhoCI density.

 Excited State   2:      Singlet-A      3.9809 eV  311.45 nm  f=0.0001  <S**2>=0.000
      82 -> 88         0.67923

 Excited State   3:      Singlet-A      4.0132 eV  308.94 nm  f=0.0006  <S**2>=0.000
      84 -> 88         0.10257
      85 -> 88        -0.10853
      86 -> 88        -0.41945
      87 -> 89         0.53520
```

3a cis-diketo

87 HOMO, 88 LUMO

```
Excited State   1:      Singlet-A      3.8096 eV  325.45 nm  f=0.0075  <S**2>=0.000
      82 -> 88         0.16969
      84 -> 88        -0.16259
      87 -> 88         0.65105
 This state for optimization and/or second-order correction.
 Total Energy, E(TD-HF/TD-DFT) =  -1071.39857189
 Copying the excited state density for this state as the 1-particle RhoCI density.

 Excited State   2:      Singlet-A      3.8330 eV  323.46 nm  f=0.0020  <S**2>=0.000
      82 -> 88        -0.40108
      82 -> 90         0.13407
      84 -> 88         0.46566
      84 -> 90        -0.12042
      87 -> 88         0.25657

 Excited State   3:      Singlet-A      3.9552 eV  313.47 nm  f=0.0010  <S**2>=0.000
      82 -> 89         0.18655
      84 -> 89         0.61163
      84 -> 91         0.18304
      86 -> 89        -0.16251

 Excited State   4:      Singlet-A      4.2665 eV  290.60 nm  f=0.1496  <S**2>=0.000
      82 -> 88        -0.19292
      86 -> 88         0.48338
      87 -> 89         0.44092
```

3a trans-diketo

87 HOMO, 88 LUMO

```
Excited State   1:      Singlet-A      3.7419 eV  331.34 nm  f=0.0831  <S**2>=0.000
      82 -> 89         0.14560
      83 -> 88         0.57985
      87 -> 88        -0.34553
 This state for optimization and/or second-order correction.
 Total Energy, E(TD-HF/TD-DFT) =  -1071.41128221
 Copying the excited state density for this state as the 1-particle RhoCI density.

 Excited State   2:      Singlet-A      3.9447 eV  314.30 nm  f=0.0212  <S**2>=0.000
      82 -> 88        -0.12465
      86 -> 88         0.66244
      87 -> 89         0.14646

 Excited State   3:      Singlet-A      3.9450 eV  314.28 nm  f=0.1423  <S**2>=0.000
      83 -> 88         0.31898
      86 -> 89         0.10968
      87 -> 88         0.59930
```

4a

98 HOMO, 99 LUMO

```
Excited State   1:      Singlet-A      3.2070 eV  386.60 nm  f=0.8815  <S**2>=0.000
      98 -> 99         0.70450
 This state for optimization and/or second-order correction.
 Total Energy, E(TD-HF/TD-DFT) =  -1295.66360691
 Copying the excited state density for this state as the 1-particle RhoCI density.

 Excited State   2:      Singlet-A      3.8302 eV  323.70 nm  f=0.0283  <S**2>=0.000
      97 -> 99         0.67666
      98 ->100         0.16215

 Excited State   3:      Singlet-A      3.9196 eV  316.32 nm  f=0.0002  <S**2>=0.000
      95 -> 99         0.65628
      97 -> 99         0.12835
      98 ->100        -0.21155
```

4b

114 HOMO, 115 LUMO

```
Excited State   1:      Singlet-A      3.2105 eV  386.18 nm  f=0.9422  <S**2>=0.000
     114 -> 115        0.70129
 This state for optimization and/or second-order correction.
 Total Energy, E(TD-HF/TD-DFT) =  -2214.92008102
 Copying the excited state density for this state as the 1-particle RhoCI density.

 Excited State   2:      Singlet-A      3.6795 eV  336.96 nm  f=0.0009  <S**2>=0.000
     111 -> 115       -0.19951
     113 -> 115        0.66962

 Excited State   3:      Singlet-A      3.7437 eV  331.18 nm  f=0.0035  <S**2>=0.000
     112 -> 115        0.69260
```

4c

114 HOMO, 115 LUMO

```
Excited State   1:      Singlet-A      3.0596 eV  405.23 nm  f=0.8498  <S**2>=0.000
     114 -> 115        0.70059
 This state for optimization and/or second-order correction.
 Total Energy, E(TD-HF/TD-DFT) =  -1524.81651337
 Copying the excited state density for this state as the 1-particle RhoCI density.

 Excited State   2:      Singlet-A      3.3875 eV  366.00 nm  f=0.0006  <S**2>=0.000
     113 -> 115        0.69728

 Excited State   3:      Singlet-A      3.6135 eV  343.12 nm  f=0.1013  <S**2>=0.000
     112 -> 115        0.69440
```

S7.2. TDA-B3LYP-D3: Excitation energies and oscillator strengths of the first excited states

3a keto-enol

87 HOMO, 88 LUMO

```
Excited State   1:      Singlet-A      3.5491 eV  349.34 nm  f=0.9917  <S**2>=0.000
      87 -> 88         0.68865
 This state for optimization and/or second-order correction.
 Total Energy, E(CIS/TDA) =  -1071.41393316
 Copying the excited state density for this state as the 1-particle RhoCI density.

 Excited State   2:      Singlet-A      3.9946 eV  310.38 nm  f=0.0001  <S**2>=0.000
      82 -> 88         0.68211
```

```
 Excited State   3:      Singlet-A      4.0481 eV  306.27 nm  f=0.0013  <S**2>=0.000
      84 -> 88         0.11597
      85 -> 88        -0.13822
      86 -> 88        -0.38677
      87 -> 89         0.55022
```

3a cis-diketo

87 HOMO, 88 LUMO

```
Excited State   1:      Singlet-A      3.8161 eV  324.90 nm  f=0.0084  <S**2>=0.000
      82 -> 88         0.10938
      87 -> 88         0.67921
 This state for optimization and/or second-order correction.
 Total Energy, E(CIS/TDA) =  -1071.39833283
 Copying the excited state density for this state as the 1-particle RhoCI density.

 Excited State   2:      Singlet-A      3.8505 eV  321.99 nm  f=0.0028  <S**2>=0.000
      82 -> 88        -0.41869
      82 -> 90         0.13853
      84 -> 88         0.48938
      84 -> 90        -0.12342
      87 -> 88         0.15888

 Excited State   3:      Singlet-A      3.9753 eV  311.89 nm  f=0.0011  <S**2>=0.000
      82 -> 89         0.18370
      84 -> 89         0.61252
      84 -> 91         0.18039
      86 -> 89        -0.16374

 Excited State   4:      Singlet-A      4.3454 eV  285.32 nm  f=0.0011  <S**2>=0.000
      85 -> 88         0.65914
      86 -> 88        -0.13378
      87 -> 89        -0.13424

 Excited State   5:      Singlet-A      4.3697 eV  283.74 nm  f=0.1722  <S**2>=0.000
      82 -> 88        -0.30926
      84 -> 88        -0.17148
      85 -> 88         0.23400
      86 -> 88         0.41641
      87 -> 89         0.30592
```

3a trans-diketo

87 HOMO, 88 LUMO

```
Excited State   1:      Singlet-A      3.7661 eV  329.21 nm  f=0.0850  <S**2>=0.000
      82 -> 89         0.15024
      83 -> 88         0.59178
      87 -> 88        -0.31966
 This state for optimization and/or second-order correction.
 Total Energy, E(CIS/TDA) =  -1071.41039462
 Copying the excited state density for this state as the 1-particle RhoCI density.

 Excited State   2:      Singlet-A      3.9927 eV  310.52 nm  f=0.1414  <S**2>=0.000
      83 -> 88         0.29240
      86 -> 89         0.19591
      87 -> 88         0.59091

 Excited State   3:      Singlet-A      3.9932 eV  310.49 nm  f=0.0186  <S**2>=0.000
      82 -> 88        -0.16690
      83 -> 89        -0.12654
      86 -> 88         0.61689
      87 -> 89         0.23781
```

4a

98 HOMO, 99 LUMO

```
Excited State   1:      Singlet-A      3.3366 eV  371.59 nm  f=1.1408  <S**2>=0.000
      98 -> 99         0.69334
 This state for optimization and/or second-order correction.
 Total Energy, E(CIS/TDA) =  -1295.65884438
```

```
 Copying the excited state density for this state as the 1-particle RhoCI density.

 Excited State   2:      Singlet-A      3.9015 eV  317.79 nm  f=0.0190  <S**2>=0.000
      95 -> 99        -0.23319
      97 -> 99         0.58747
      98 ->100         0.29024

 Excited State   3:      Singlet-A      3.9639 eV  312.78 nm  f=0.0017  <S**2>=0.000
      95 -> 99         0.62057
      97 -> 99         0.29500
      98 ->100        -0.11722
```

4b

114 HOMO, 115 LUMO

```
Excited State   1:      Singlet-A      3.3228 eV  373.13 nm  f=1.1695  <S**2>=0.000
     112 -> 115       -0.14749
     114 -> 115        0.68405
 This state for optimization and/or second-order correction.
 Total Energy, E(CIS/TDA) =  -2214.91595357
 Copying the excited state density for this state as the 1-particle RhoCI density.

 Excited State   2:      Singlet-A      3.7238 eV  332.95 nm  f=0.0026  <S**2>=0.000
     111 -> 115       -0.25441
     113 -> 115        0.64664

 Excited State   3:      Singlet-A      3.8001 eV  326.27 nm  f=0.0386  <S**2>=0.000
     112 -> 115        0.67468
     114 -> 115        0.13424
```

4c

114 HOMO, 115 LUMO

```
Excited State   1:      Singlet-A      3.1431 eV  394.47 nm  f=0.9753  <S**2>=0.000
     112 -> 115       -0.15674
     114 -> 115        0.68445
 This state for optimization and/or second-order correction.
 Total Energy, E(CIS/TDA) =  -1524.81344658
 Copying the excited state density for this state as the 1-particle RhoCI density.

 Excited State   2:      Singlet-A      3.4386 eV  360.56 nm  f=0.0014  <S**2>=0.000
     111 -> 115       -0.11489
     113 -> 115        0.68862

 Excited State   3:      Singlet-A      3.7037 eV  334.76 nm  f=0.2405  <S**2>=0.000
     112 -> 115        0.67211
     114 -> 115        0.14773

 Excited State   4:      Singlet-A      3.9019 eV  317.75 nm  f=0.0002  <S**2>=0.000
     111 -> 115        0.45519
     114 -> 116        0.52300

 Excited State   5:      Singlet-A      4.2324 eV  292.94 nm  f=0.1124  <S**2>=0.000
     109 -> 115       -0.12204
     111 -> 115        0.49539
     114 -> 116       -0.44998
```

S7.3. TDA-ωB97X-D: Excitation energies and oscillator strengths of the first excited states

3a keto-enol

87 HOMO, 88 LUMO

```
Excited State   1:      Singlet-A      4.0442 eV  306.58 nm  f=1.1314  <S**2>=0.000
      83 ->  88        0.11660
      87 ->  88        0.66536
 This state for optimization and/or second-order correction.
 Total Energy, E(CIS/TDA) =  -1070.93226768
 Copying the excited state density for this state as the 1-particle RhoCI density.
```

```
 Excited State   2:      Singlet-A      4.3596 eV  284.39 nm  f=0.0009  <S**2>=0.000
      82 ->  88        0.64539
      82 ->  89        0.11441
      82 ->  91        0.15711
      83 ->  88        0.10033

 Excited State   3:      Singlet-A      4.8527 eV  255.50 nm  f=0.1610  <S**2>=0.000
      84 ->  88       -0.15318
      84 ->  96        0.11778
      86 ->  88       -0.33196
      86 ->  91        0.15728
      87 ->  89        0.51507
      87 ->  91        0.11107
```

3a cis-diketo

87 HOMO, 88 LUMO

```
Excited State   1:      Singlet-A      4.1641 eV  297.75 nm  f=0.0021  <S**2>=0.000
      82 ->  88        0.46321
      82 ->  89       -0.10256
      82 ->  91       -0.25114
      83 ->  88       -0.35194
      83 ->  89        0.11171
      83 ->  91        0.18796
 This state for optimization and/or second-order correction.
 Total Energy, E(CIS/TDA) =  -1070.92552459
 Copying the excited state density for this state as the 1-particle RhoCI density.

 Excited State   2:      Singlet-A      4.2544 eV  291.42 nm  f=0.0007  <S**2>=0.000
      82 ->  89        0.23394
      82 ->  93        0.12336
      83 ->  88        0.12474
      83 ->  89        0.53174
      83 ->  93        0.28043

 Excited State   3:      Singlet-A      4.9015 eV  252.95 nm  f=0.1682  <S**2>=0.000
      86 ->  88        0.35050
      87 ->  88        0.24831
      87 ->  89        0.49118

 Excited State   4:      Singlet-A      4.9949 eV  248.22 nm  f=0.3387  <S**2>=0.000
      84 ->  88        0.10186
      84 ->  91       -0.10223
      84 ->  95        0.11479
      86 ->  88        0.53685
      87 ->  88       -0.15358
      87 ->  89       -0.30638

 Excited State   5:      Singlet-A      5.0172 eV  247.12 nm  f=0.0180  <S**2>=0.000
      84 ->  88        0.14681
      85 ->  88        0.14524
      85 ->  89        0.46564
      85 ->  93       -0.15392
      87 ->  88        0.10567
      87 ->  93       -0.29011
      87 ->  98        0.20787
```

3a trans-diketo

87 HOMO, 88 LUMO

```
Excitation energies and oscillator strengths:

 Excited State   1:      Singlet-A      4.1293 eV  300.26 nm  f=0.0207  <S**2>=0.000
      82 ->  89        0.22324
      82 ->  93       -0.17836
      83 ->  88        0.59029
      83 ->  90       -0.16359
      87 ->  88        0.11313
 This state for optimization and/or second-order correction.
 Total Energy, E(CIS/TDA) =  -1070.93622230
 Copying the excited state density for this state as the 1-particle RhoCI density.

 Excited State   2:      Singlet-A      4.3627 eV  284.19 nm  f=0.0027  <S**2>=0.000
      82 ->  88        0.47919
```

```
      82 ->  90       -0.14227
      83 ->  89        0.37114
      83 ->  93       -0.26375

 Excited State   3:      Singlet-A      4.7932 eV  258.67 nm  f=0.4866  <S**2>=0.000
      86 ->  89        0.23471
      87 ->  88        0.60854
```

4a

98 HOMO, 99 LUMO

```
Excited State   1:      Singlet-A      3.7600 eV  329.75 nm  f=1.2508  <S**2>=0.000
      94 ->  99       -0.13207
      98 ->  99        0.66959
 This state for optimization and/or second-order correction.
 Total Energy, E(CIS/TDA) =  -1295.11002172
 Copying the excited state density for this state as the 1-particle RhoCI density.

 Excited State   2:      Singlet-A      4.7716 eV  259.84 nm  f=0.1825  <S**2>=0.000
      95 -> 102        0.10139
      97 ->  99        0.57703
      98 -> 100       -0.31810

 Excited State   3:      Singlet-A      4.8580 eV  255.22 nm  f=0.0001  <S**2>=0.000
      95 ->  99        0.47783
      96 -> 100        0.24504
      97 ->  99       -0.12465
      97 -> 102       -0.20931
      98 -> 100       -0.28672
      98 -> 106       -0.14907
```

4b

114 HOMO, 115 LUMO

```
Excited State   1:      Singlet-A      3.7686 eV  328.99 nm  f=1.3250  <S**2>=0.000
     110 -> 115       -0.12484
     114 -> 115        0.66924
 This state for optimization and/or second-order correction.
 Total Energy, E(CIS/TDA) =  -2214.33140936
 Copying the excited state density for this state as the 1-particle RhoCI density.

 Excited State   2:      Singlet-A      4.7138 eV  263.02 nm  f=0.0280  <S**2>=0.000
     112 -> 116       -0.12851
     112 -> 121       -0.11189
     113 -> 115        0.49343
     113 -> 117       -0.13983
     114 -> 116       -0.39032

 Excited State   3:      Singlet-A      4.7638 eV  260.26 nm  f=0.0431  <S**2>=0.000
     111 -> 116        0.11366
     111 -> 121        0.14911
     112 -> 115        0.47669
     112 -> 117       -0.14570
     113 -> 116       -0.31530
     114 -> 117       -0.24636
     114 -> 122        0.11315
```

4c

114 HOMO, 115 LUMO

```
Excited State   1:      Singlet-A      3.6857 eV  336.40 nm  f=1.3609  <S**2>=0.000
     110 -> 115       -0.11926
     112 -> 115       -0.11490
     113 -> 116       -0.10371
     114 -> 115        0.65979
 This state for optimization and/or second-order correction.
 Total Energy, E(CIS/TDA) =  -1524.17658990
 Copying the excited state density for this state as the 1-particle RhoCI density.

 Excited State   2:      Singlet-A      4.4779 eV  276.88 nm  f=0.0227  <S**2>=0.000
     113 -> 115        0.55153
```

```
     113 -> 118       -0.12259
     114 -> 116       -0.36084

 Excited State   3:      Singlet-A      4.5964 eV  269.74 nm  f=0.0219  <S**2>=0.000
     111 -> 127        0.12685
     112 -> 115        0.48297
     113 -> 116       -0.34185
     114 -> 118       -0.27987
```

S7.4. TDA-M06-2X: Excitation energies and oscillator strengths of the first excited states

3a keto-enol

87 HOMO, 88 LUMO

```
Excited State   1:      Singlet-A      3.9629 eV  312.86 nm  f=1.1661  <S**2>=0.000
      87 ->  88        0.67537
 This state for optimization and/or second-order correction.
 Total Energy, E(CIS/TDA) =  -1070.86805747
 Copying the excited state density for this state as the 1-particle RhoCI density.

 Excited State   2:      Singlet-A      4.3550 eV  284.70 nm  f=0.0001  <S**2>=0.000
      82 ->  88        0.63773
      82 ->  90       -0.12554
      82 ->  92        0.16984
      82 -> 102       -0.11073

 Excited State   3:      Singlet-A      4.7764 eV  259.57 nm  f=0.1360  <S**2>=0.000
      84 ->  88        0.17203
      86 ->  88        0.27093
      86 ->  92       -0.12580
      87 ->  90        0.56117
      87 ->  92       -0.12231

 Excited State   4:      Singlet-A      4.8098 eV  257.78 nm  f=0.0031  <S**2>=0.000
      86 ->  91       -0.10399
      87 ->  89        0.64902
      87 ->  93        0.17301
```

3a cis-diketo

87 HOMO, 88 LUMO

```
Excited State   1:      Singlet-A      4.0458 eV  306.45 nm  f=0.0169  <S**2>=0.000
      82 ->  89       -0.22005
      82 ->  93       -0.11152
      82 ->  95        0.16970
      83 ->  88        0.55502
      83 ->  92       -0.18225
      87 ->  88        0.10803
 This state for optimization and/or second-order correction.
 Total Energy, E(CIS/TDA) =  -1070.86858006
 Copying the excited state density for this state as the 1-particle RhoCI density.

 Excited State   2:      Singlet-A      4.2341 eV  292.82 nm  f=0.0026  <S**2>=0.000
      82 ->  88        0.44624
      82 ->  92       -0.15527
      83 ->  89       -0.33538
      83 ->  93       -0.15883
      83 ->  95        0.23885

 Excited State   3:      Singlet-A      4.7160 eV  262.90 nm  f=0.4831  <S**2>=0.000
      86 ->  89        0.15947
      87 ->  88        0.64319

 Excited State   4:      Singlet-A      4.7554 eV  260.73 nm  f=0.0523  <S**2>=0.000
      86 ->  88        0.63397
      87 ->  89        0.19995

 Excited State   5:      Singlet-A      5.0235 eV  246.81 nm  f=0.0455  <S**2>=0.000
      84 ->  89       -0.31351
      85 ->  88        0.45831
      85 ->  92        0.11502
```

```
      86 ->  89         0.15245
      86 ->  95         0.13755
      86 -> 101         0.10200
      87 ->  92         0.24128
      87 -> 102        -0.12257
```

3a trans-diketo

87 HOMO, 88 LUMO

```
Excited State   1:      Singlet-A      4.0458 eV  306.45 nm  f=0.0169  <S**2>=0.000
      82 ->  89        -0.22008
      82 ->  93         0.11158
      82 ->  95        -0.16965
      83 ->  88         0.55501
      83 ->  92        -0.18222
      87 ->  88         0.10816
 This state for optimization and/or second-order correction.
 Total Energy, E(CIS/TDA) =  -1070.86857954
 Copying the excited state density for this state as the 1-particle RhoCI density.

 Excited State   2:      Singlet-A      4.2341 eV  292.82 nm  f=0.0026  <S**2>=0.000
      82 ->  88         0.44625
      82 ->  92        -0.15526
      83 ->  89        -0.33542
      83 ->  93         0.15893
      83 ->  95        -0.23877

 Excited State   3:      Singlet-A      4.7160 eV  262.90 nm  f=0.4829  <S**2>=0.000
      86 ->  89         0.15943
      87 ->  88         0.64318
```

4a

98 HOMO, 99 LUMO

```
Excited State   1:      Singlet-A      3.6760 eV  337.28 nm  f=1.2909  <S**2>=0.000
      94 ->  99        -0.10776
      98 ->  99         0.67919
 This state for optimization and/or second-order correction.
 Total Energy, E(CIS/TDA) =  -1295.05039901
 Copying the excited state density for this state as the 1-particle RhoCI density.

 Excited State   2:      Singlet-A      4.6698 eV  265.50 nm  f=0.1373  <S**2>=0.000
      97 ->  99         0.63999
      98 -> 101        -0.19149

 Excited State   3:      Singlet-A      4.7548 eV  260.76 nm  f=0.0014  <S**2>=0.000
      95 ->  99         0.49836
      96 -> 101         0.15667
      97 ->  99        -0.13493
      97 -> 103        -0.16909
      98 -> 100         0.12466
      98 -> 101        -0.35686
```

4b

114 HOMO, 115 LUMO

```
Excited State   1:      Singlet-A      3.6835 eV  336.60 nm  f=1.3679  <S**2>=0.000
     110 -> 115        -0.10316
     114 -> 115         0.67952
 This state for optimization and/or second-order correction.
 Total Energy, E(CIS/TDA) =  -2214.25683363
 Copying the excited state density for this state as the 1-particle RhoCI density.

 Excited State   2:      Singlet-A      4.5870 eV  270.29 nm  f=0.0163  <S**2>=0.000
     111 -> 115        -0.10000
     113 -> 115         0.58309
     114 -> 116        -0.32225

 Excited State   3:      Singlet-A      4.6655 eV  265.75 nm  f=0.0148  <S**2>=0.000
     111 -> 123         0.10704
     112 -> 115         0.58978
     113 -> 116        -0.23552
```

```
     114 -> 118       -0.21862
```

4c

114 HOMO, 115 LUMO

```
Excited State   1:      Singlet-A      3.5958 eV  344.81 nm  f=1.3811  <S**2>=0.000
     114 -> 115        0.67700
 This state for optimization and/or second-order correction.
 Total Energy, E(CIS/TDA) =  -1524.09980894
 Copying the excited state density for this state as the 1-particle RhoCI density.

 Excited State   2:      Singlet-A      4.3489 eV  285.09 nm  f=0.0134  <S**2>=0.000
     113 -> 115        0.63020
     114 -> 116        0.10965
     114 -> 117       -0.24573

 Excited State   3:      Singlet-A      4.5163 eV  274.53 nm  f=0.0004  <S**2>=0.000
     112 -> 115        0.59954
     113 -> 117       -0.22164
     114 -> 119       -0.21858
```

S7.5. TDA-CAM-B3LYP-D3: Excitation energies and oscillator strengths of the first excited states

3a keto-enol

87 HOMO, 88 LUMO

```
Excited State   1:      Singlet-A      3.9641 eV  312.77 nm  f=1.1325  <S**2>=0.000
      83 ->  88        0.10074
      87 ->  88        0.67289
 This state for optimization and/or second-order correction.
 Total Energy, E(CIS/TDA) =  -1070.79296311
 Copying the excited state density for this state as the 1-particle RhoCI density.

 Excited State   2:      Singlet-A      4.4009 eV  281.72 nm  f=0.0001  <S**2>=0.000
      82 ->  88        0.65290
      82 ->  89        0.12235
      82 ->  92        0.16258

 Excited State   3:      Singlet-A      4.7644 eV  260.23 nm  f=0.1473  <S**2>=0.000
      84 ->  88       -0.15899
      84 ->  96        0.10652
      86 ->  88       -0.28953
      86 ->  92        0.13893
      87 ->  89        0.55348
      87 ->  92        0.10465
```

3a cis-diketo

87 HOMO, 88 LUMO

```
Excited State   1:      Singlet-A      4.1779 eV  296.76 nm  f=0.0017  <S**2>=0.000
      82 ->  88        0.46503
      82 ->  91        0.25389
      83 ->  88        0.34916
      83 ->  89       -0.11163
      83 ->  91        0.18547
 This state for optimization and/or second-order correction.
 Total Energy, E(CIS/TDA) =  -1070.77942857
 Copying the excited state density for this state as the 1-particle RhoCI density.

 Excited State   2:      Singlet-A      4.2617 eV  290.92 nm  f=0.0006  <S**2>=0.000
      82 ->  89       -0.22593
      82 ->  94        0.12422
      83 ->  88        0.11363
      83 ->  89        0.52285
      83 ->  90       -0.12195
      83 ->  94       -0.28656

 Excited State   3:      Singlet-A      4.8528 eV  255.49 nm  f=0.1571  <S**2>=0.000
      86 ->  88        0.30873
      87 ->  88        0.34030
```

```
     87 ->  89        0.46278

 Excited State   4:      Singlet-A      4.9498 eV  250.48 nm  f=0.3211  <S**2>=0.000
     84 ->  91        0.11146
     84 ->  97       -0.10318
     86 ->  88        0.56810
     87 ->  88       -0.20175
     87 ->  89       -0.22410

 Excited State   5:      Singlet-A      4.9925 eV  248.34 nm  f=0.0225  <S**2>=0.000
     84 ->  88        0.10528
     85 ->  88        0.12322
     85 ->  89        0.43328
     85 ->  90       -0.10180
     85 ->  94        0.14229
     87 ->  88        0.19197
     87 ->  89       -0.20233
     87 ->  94        0.26624
     87 ->  99       -0.12783
     87 -> 100        0.11226
     87 -> 101       -0.11527
```

3a trans-diketo

87 HOMO, 88 LUMO

```
Excited State   1:      Singlet-A      4.1349 eV  299.85 nm  f=0.0212  <S**2>=0.000
     82 ->  89       -0.22255
     82 ->  93        0.17606
     83 ->  88        0.58699
     83 ->  92       -0.16496
     87 ->  88        0.11999
 This state for optimization and/or second-order correction.
 Total Energy, E(CIS/TDA) =  -1070.79082359
 Copying the excited state density for this state as the 1-particle RhoCI density.

 Excited State   2:      Singlet-A      4.3668 eV  283.93 nm  f=0.0033  <S**2>=0.000
     82 ->  88        0.47661
     82 ->  92       -0.14440
     83 ->  89       -0.36777
     83 ->  93        0.25877

 Excited State   3:      Singlet-A      4.7218 eV  262.58 nm  f=0.4670  <S**2>=0.000
     86 ->  89        0.18284
     87 ->  88        0.63279

 Excited State   4:      Singlet-A      4.7505 eV  260.99 nm  f=0.0496  <S**2>=0.000
     84 ->  92        0.10658
     86 ->  88        0.61653
     87 ->  89        0.22490

 Excited State   5:      Singlet-A      4.9806 eV  248.93 nm  f=0.0510  <S**2>=0.000
     84 ->  89        0.32872
     85 ->  88        0.45321
     85 ->  92        0.11557
     86 ->  89       -0.13535
     86 ->  93       -0.16968
     86 ->  97        0.11895
     87 ->  92       -0.23559
     87 ->  98       -0.12718
```

4a

98 HOMO, 99 LUMO

```
Excited State   1:      Singlet-A      3.7065 eV  334.51 nm  f=1.2530  <S**2>=0.000
     94 ->  99        0.11252
     98 ->  99        0.67648
 This state for optimization and/or second-order correction.
 Total Energy, E(CIS/TDA) =  -1294.98467519
 Copying the excited state density for this state as the 1-particle RhoCI density.

 Excited State   2:      Singlet-A      4.6928 eV  264.20 nm  f=0.1657  <S**2>=0.000
     97 ->  99        0.60405
     98 -> 100       -0.28870
```

```
 Excited State   3:      Singlet-A      4.7604 eV  260.45 nm  f=0.0001  <S**2>=0.000
      95 ->  99         0.50282
      96 -> 100         0.19871
      97 ->  99         0.15992
      97 -> 103         0.18332
      98 -> 100         0.31898
      98 -> 107         0.12002
```

4b

114 HOMO, 115 LUMO

```
Excited State   1:      Singlet-A      3.7156 eV  333.68 nm  f=1.3283  <S**2>=0.000
     110 -> 115         0.10701
     114 -> 115         0.67646
 This state for optimization and/or second-order correction.
 Total Energy, E(CIS/TDA) =  -2214.24613977
 Copying the excited state density for this state as the 1-particle RhoCI density.

 Excited State   2:      Singlet-A      4.6102 eV  268.93 nm  f=0.0163  <S**2>=0.000
     111 -> 115        -0.13431
     112 -> 116        -0.10384
     113 -> 115         0.53778
     114 -> 116         0.35674

 Excited State   3:      Singlet-A      4.6716 eV  265.40 nm  f=0.0274  <S**2>=0.000
     111 -> 121         0.12260
     112 -> 115         0.55712
     113 -> 116        -0.26800
     114 -> 118         0.22231

 Excited State   4:      Singlet-A      4.7522 eV  260.90 nm  f=0.0776  <S**2>=0.000
     111 -> 115         0.52307
     112 -> 116         0.23946
     113 -> 115         0.22574
     113 -> 118         0.21945
     114 -> 121         0.16116

 Excited State   5:      Singlet-A      5.0066 eV  247.64 nm  f=0.1107  <S**2>=0.000
     111 -> 118        -0.10566
     112 -> 116         0.12742
     113 -> 115        -0.35434
     114 -> 116         0.54050
```

4c

114 HOMO, 115 LUMO

```
Excited State   1:      Singlet-A      3.6268 eV  341.85 nm  f=1.3519  <S**2>=0.000
     110 -> 115         0.10187
     114 -> 115         0.67085
 This state for optimization and/or second-order correction.
 Total Energy, E(CIS/TDA) =  -1524.04043612
 Copying the excited state density for this state as the 1-particle RhoCI density.

 Excited State   2:      Singlet-A      4.3615 eV  284.27 nm  f=0.0132  <S**2>=0.000
     113 -> 115         0.60504
     114 -> 116         0.30459

 Excited State   3:      Singlet-A      4.5069 eV  275.10 nm  f=0.0046  <S**2>=0.000
     111 -> 129         0.10380
     112 -> 115         0.56684
     113 -> 116        -0.28012
     114 -> 119        -0.22848

 Excited State   4:      Singlet-A      4.6826 eV  264.78 nm  f=0.0067  <S**2>=0.000
     111 -> 115         0.40546
     112 -> 116         0.20431
     113 -> 115         0.24377
     113 -> 119        -0.30455
     114 -> 116        -0.27631
     114 -> 129         0.12609

 Excited State   5:      Singlet-A      4.8875 eV  253.68 nm  f=0.2098  <S**2>=0.000
```

```
     111 -> 115        0.30616
     112 -> 116        0.25966
     113 -> 115       -0.21638
     114 -> 116        0.48503
```

S7.3. TD-ωB97X-D: Excitation energies and oscillator strengths of the first excited states

3a keto-enol

87 HOMO, 88 LUMO

```
Excited State   1:      Singlet-A      3.9016 eV  317.78 nm  f=0.9123  <S**2>=0.000
      83 ->  88        0.13946
      86 ->  89       -0.10641
      87 ->  88        0.66706
 This state for optimization and/or second-order correction.
 Total Energy, E(TD-HF/TD-DFT) =  -1070.93750704
 Copying the excited state density for this state as the 1-particle RhoCI density.

 Excited State   2:      Singlet-A      4.3362 eV  285.93 nm  f=0.0006  <S**2>=0.000
      82 ->  88        0.64507
      82 ->  89        0.11498
      82 ->  91        0.15750
      83 ->  88        0.10138

 Excited State   3:      Singlet-A      4.6779 eV  265.04 nm  f=0.1734  <S**2>=0.000
      86 ->  88       -0.41743
      86 ->  91        0.14807
      87 ->  89        0.50054

 Excited State   4:      Singlet-A      4.8843 eV  253.84 nm  f=0.0122  <S**2>=0.000
      84 ->  88       -0.29301
      84 ->  89        0.24499
      84 ->  91        0.14525
      85 ->  88        0.15980
      86 ->  88        0.17677
      86 ->  89        0.26976
      86 ->  96       -0.11071
      87 ->  91        0.33302
      87 ->  96       -0.12250

 Excited State   5:      Singlet-A      4.9032 eV  252.87 nm  f=0.0087  <S**2>=0.000
      84 ->  88        0.13896
      84 ->  89       -0.10884
      85 ->  88        0.32141
      85 ->  89        0.31754
      85 ->  91       -0.18387
      86 ->  88       -0.23053
      86 ->  91       -0.23172
      86 ->  97       -0.16957
      87 ->  89       -0.12062
      87 ->  96        0.12134
      87 ->  97        0.13343
```

3a cis-diketo

87 HOMO, 88 LUMO

```
Excited State   1:      Singlet-A      4.1346 eV  299.87 nm  f=0.0020  <S**2>=0.000
      82 ->  88        0.46341
      82 ->  89       -0.10278
      82 ->  91       -0.25212
      83 ->  88       -0.35098
      83 ->  89        0.11241
      83 ->  91        0.18863
 This state for optimization and/or second-order correction.
 Total Energy, E(TD-HF/TD-DFT) =  -1070.92660669
 Copying the excited state density for this state as the 1-particle RhoCI density.

 Excited State   2:      Singlet-A      4.2242 eV  293.51 nm  f=0.0006  <S**2>=0.000
      82 ->  89        0.23480
      82 ->  93        0.12407
      83 ->  88        0.12462
      83 ->  89        0.53109
```

```
     83 ->  93        0.28142

 Excited State   3:      Singlet-A      4.7166 eV  262.87 nm  f=0.1403  <S**2>=0.000
     86 ->  88        0.35015
     87 ->  88        0.22517
     87 ->  89        0.52687

 Excited State   4:      Singlet-A      4.8000 eV  258.30 nm  f=0.2824  <S**2>=0.000
     86 ->  88        0.56583
     87 ->  88       -0.10361
     87 ->  89       -0.33578

 Excited State   5:      Singlet-A      4.9327 eV  251.35 nm  f=0.0341  <S**2>=0.000
     84 ->  88        0.19235
     85 ->  88        0.15281
     85 ->  89        0.47810
     85 ->  93       -0.16407
     86 ->  91        0.10137
     87 ->  93       -0.27254
     87 ->  98        0.18316
```

3a trans-diketo

87 HOMO, 88 LUMO

```
Excitation energies and oscillator strengths:

 Excited State   1:      Singlet-A      4.1012 eV  302.31 nm  f=0.0189  <S**2>=0.000
     82 ->  89        0.22287
     82 ->  93       -0.17807
     83 ->  88        0.59010
     83 ->  90       -0.16479
     87 ->  88        0.11583
 This state for optimization and/or second-order correction.
 Total Energy, E(TD-HF/TD-DFT) =  -1070.93725221
 Copying the excited state density for this state as the 1-particle RhoCI density.

 Excited State   2:      Singlet-A      4.3316 eV  286.23 nm  f=0.0027  <S**2>=0.000
     82 ->  88        0.47829
     82 ->  90       -0.14284
     83 ->  89        0.37109
     83 ->  93       -0.26471

 Excited State   3:      Singlet-A      4.6346 eV  267.52 nm  f=0.4189  <S**2>=0.000
     86 ->  89        0.29562
     87 ->  88        0.59945

 Excited State   4:      Singlet-A      4.6373 eV  267.36 nm  f=0.0414  <S**2>=0.000
     86 ->  88        0.58281
     87 ->  89        0.32970

 Excited State   5:      Singlet-A      4.9272 eV  251.63 nm  f=0.0439  <S**2>=0.000
     84 ->  89        0.36085
     85 ->  88        0.41951
     85 ->  90        0.16037
     86 ->  89        0.10856
     86 ->  93        0.18138
     86 ->  97       -0.12495
     87 ->  90        0.23506
     87 ->  98       -0.11822
```

4a

98 HOMO, 99 LUMO

```
Excitation energies and oscillator strengths:

 Excited State   1:      Singlet-A      3.6254 eV  341.98 nm  f=1.0097  <S**2>=0.000
     94 ->  99       -0.15585
     98 ->  99        0.67085
 This state for optimization and/or second-order correction.
 Total Energy, E(TD-HF/TD-DFT) =  -1295.11496532
 Copying the excited state density for this state as the 1-particle RhoCI density.

 Excited State   2:      Singlet-A      4.5762 eV  270.94 nm  f=0.1701  <S**2>=0.000
```

```
      97 ->  99         0.56293
      98 -> 100        -0.36801

 Excited State   3:      Singlet-A      4.7832 eV  259.21 nm  f=0.0003  <S**2>=0.000
      95 ->  99         0.47658
      95 -> 102        -0.11702
      96 -> 100         0.27009
      97 ->  99        -0.14143
      97 -> 102        -0.21204
      98 -> 100        -0.25790
      98 -> 106        -0.14636

 Excited State   4:      Singlet-A      4.8025 eV  258.17 nm  f=0.0648  <S**2>=0.000
      95 -> 100         0.28352
      96 ->  99         0.50349
      96 -> 102        -0.14362
      97 -> 100         0.17187
      97 -> 106         0.13438
      98 -> 102         0.23073
      98 -> 108         0.10280

 Excited State   5:      Singlet-A      5.0807 eV  244.03 nm  f=0.1051  <S**2>=0.000
      94 ->  99         0.45014
      95 -> 100         0.18933
      96 -> 102        -0.18454
      97 -> 100        -0.29876
      98 -> 102        -0.27431
      98 -> 108         0.11797
```

4b

114 HOMO, 115 LUMO

```
Excitation energies and oscillator strengths:

 Excited State   1:      Singlet-A      3.6388 eV  340.73 nm  f=1.0796  <S**2>=0.000
     110 -> 115        -0.14825
     114 -> 115         0.66936
 This state for optimization and/or second-order correction.
 Total Energy, E(TD-HF/TD-DFT) =  -2214.33618114
 Copying the excited state density for this state as the 1-particle RhoCI density.

 Excited State   2:      Singlet-A      4.5644 eV  271.63 nm  f=0.0748  <S**2>=0.000
     111 -> 115         0.13026
     111 -> 117        -0.10265
     112 -> 121        -0.10000
     113 -> 115         0.49870
     114 -> 116        -0.41782

 Excited State   3:      Singlet-A      4.6708 eV  265.45 nm  f=0.0575  <S**2>=0.000
     111 -> 116         0.11549
     111 -> 121         0.13595
     112 -> 115         0.47184
     112 -> 117        -0.16465
     113 -> 116        -0.33965
     114 -> 117        -0.24691

 Excited State   4:      Singlet-A      4.7037 eV  263.59 nm  f=0.0509  <S**2>=0.000
     111 -> 115         0.49841
     112 -> 116         0.31817
     113 -> 117         0.26383
     114 -> 121        -0.17568

 Excited State   5:      Singlet-A      5.0308 eV  246.45 nm  f=0.0829  <S**2>=0.000
     110 -> 115         0.36462
     111 -> 116        -0.32967
     112 -> 115         0.18564
     112 -> 117         0.31604
     113 -> 121        -0.18041
     114 -> 117        -0.16958
     114 -> 122        -0.15799
```

4c

114 HOMO, 115 LUMO

```
Excitation energies and oscillator strengths:

 Excited State   1:      Singlet-A      3.5655 eV  347.73 nm  f=1.1315  <S**2>=0.000
     110 -> 115       -0.13902
     112 -> 115       -0.14567
     113 -> 116       -0.10200
     114 -> 115        0.65508
 This state for optimization and/or second-order correction.
 Total Energy, E(TD-HF/TD-DFT) =  -1524.18100447
 Copying the excited state density for this state as the 1-particle RhoCI density.

 Excited State   2:      Singlet-A      4.3391 eV  285.74 nm  f=0.0336  <S**2>=0.000
     113 -> 115        0.52757
     113 -> 118       -0.15175
     114 -> 116       -0.39917

 Excited State   3:      Singlet-A      4.4691 eV  277.42 nm  f=0.0391  <S**2>=0.000
     111 -> 127        0.10889
     112 -> 115        0.45614
     112 -> 118       -0.12401
     113 -> 116       -0.37477
     114 -> 118       -0.29415

 Excited State   4:      Singlet-A      4.6275 eV  267.93 nm  f=0.0395  <S**2>=0.000
     111 -> 115        0.43794
     112 -> 116        0.30124
     113 -> 115        0.17670
     113 -> 118        0.32155
     114 -> 124        0.13538
     114 -> 127       -0.13597

 Excited State   5:      Singlet-A      4.8964 eV  253.21 nm  f=0.1641  <S**2>=0.000
     111 -> 115       -0.22738
     111 -> 118        0.10715
     112 -> 116       -0.20511
     112 -> 124        0.11024
     113 -> 115        0.38124
     113 -> 118        0.17603
     114 -> 116        0.43332
```

S8. Supplementary electronic-state transition spectroscopy data

Table S2. Fluorescence decay times, τ_i, and relative amplitudes, f_i, of compounds **3a**, **3b**, and **3c**, as retrieved from fitting the experimental decay patterns measured upon excitation at 280 nm to a biexponential decay model function. The reported values are averaged over three parallels. The pertaining errors are the corresponding standard deviations.

Compound	Solvent	τ_1 (ps)	f_1	τ_2 (ps)	f_2
**3a**	ethyl acetate	-	0	2072 ± 11	1
	dichloromethane	222 ± 1	0.93 ± 0.01	2248 ± 4	0.07 ± 0.01
	acetonitrile	209 ± 1	0.97 ± 0.01	2368 ± 24	0.03 ± 0.01
	dimethylformamide	249 ± 1	0.58 ± 0.01	2163 ± 1	0.42 ± 0.01
	dimethyl sulfoxide	258 ± 1	0.94 ± 0.01	2188 ± 24	0.06 ± 0.01
	butanol	431 ± 1	0.98 ± 0.01	2653 ± 1	0.02 ± 0.01
	ethanol	360 ± 1	≅ 1	≅ 3660	<0.01
	methanol	388 ± 2	0.97 ± 0.01	2434 ± 21	0.03 ± 0.01
**3b**	ethyl acetate	202 ± 2	0.95 ± 0.01	1940 ± 8	0.05 ± 0.01
	dichloromethane	262 ± 3	0.97 ± 0.01	2057 ± 13	0.03 ± 0.01
	acetonitrile	205 ± 2	0.96 ± 0.01	2207 ± 29	0.04 ± 0.01
	dimethylformamide	257 ± 2	0.91 ± 0.01	2933 ± 1	0.09 ± 0.01
	dimethyl sulfoxide	271 ± 1	0.96 ± 0.01	2471 ± 5	0.04 ± 0.01
	butanol	451 ± 4	0.75 ± 0.01	4011 ± 20	0.25 ± 0.01
	ethanol	281 ± 2	0.99 ± 0.01	2612 ± 71	0.01 ± 0.01
	methanol	275 ± 1	0.89 ± 0.01	2460 ± 14	0.11 ± 0.01
**3c**	ethyl acetate	309 ± 2	0.89 ± 0.03	1669 ± 22	0.11 ± 0.03
	dichloromethane	735 ± 52	0.30 ± 0.01	1602 ± 17	0.70 ± 0.01
	acetonitrile	501 ± 15	0.30 ± 0.01	1863 ± 9	0.70 ± 0.01
	dimethylformamide	362 ± 5	0.76 ± 0.01	2325 ± 7	0.24 ± 0.01
	dimethyl sulfoxide	406 ± 3	0.71 ± 0.01	2232 ± 4	0.29 ± 0.01
	butanol	730 ± 6	0.37 ± 0.01	2501 ± 6	0.63 ± 0.01
	ethanol	644 ± 23	0.45 ± 0.01	1590 ± 12	0.55 ± 0.01
	methanol	545 ± 1	0.98 ± 0.01	2657 ± 24	0.02 ± 0.01

Table S3. Fluorescence decay times, τ_i, and relative amplitudes, f_i, of compounds **3a**, **3b**, and **3c**, as retrieved from fitting the experimental decay patterns measured upon excitation at 420 nm to a biexponential decay model function. The reported values are averaged over three parallels. The pertaining errors are the corresponding standard deviations.

Compound	Solvent	τ_1 (ps)	f_1	τ_2 (ps)	f_2	τ_3 (ps)	f_3
**3a**	toluene	-	0	361 ± 3	0.87± 0.01	2058 ± 5	0.13 ± 0.01
	ethyl acetate	30 ± 2	0.13 ± 0.01	-	0	2059 ± 3	0.87 ± 0.01
	dichloromethane	11 ± 4	0.28 ± 0.04	282 ± 2	0.63 ± 0.03	2467 ± 1	0.09 ± 0.01
	acetone	19 ± 1	0.37 ± 0.02	277 ± 1	0.21 ± 0.01	2154 ± 8	0.42 ± 0.03
	acetonitrile	16 ± 1	0.30 ± 0.03	243 ± 2	0.63 ± 0.03	2454 ± 18	0.07 ± 0.01
	dimethylformamide	-	0	273 ± 8	0.32 ± 0.01	2098 ± 1	0.68 ± 0.01
	dimethyl sulfoxide	-	0	291 ± 4	0.92 ± 0.01	2244 ± 9	0.08 ± 0.01
	butanol	-	0	405 ± 6	≅ 1	≅ 3700	<0.001
	ethanol	-	0	353 ± 10	≅ 1	≅ 2800	<0.01
	methanol	-	0	371 ± 6	0.96 ± 0.01	2224 ± 1	0.04 ± 0.01
**3b**	toluene	-	0	412 ± 4	0.94 ± 0.01	1269 ± 24	0.06 ± 0.01
	ethyl acetate	29 ± 2	0.35 ± 0.01	213 ± 1	0.64 ± 0.01	2226 ± 7	0.01 ± 0.01
	dichloromethane	-	0	320 ± 1	0.92 ± 0.01	2336 ± 6	0.08 ± 0.01
	acetone	53 ± 3	0.46 ± 0.01	202 ± 3	0.53 ± 0.01	2010 ± 46	0.01 ± 0.01
	acetonitrile	51 ± 1	0.61 ± 0.01	236 ± 1	0.33 ± 0.01	2069 ± 2	0.06 ± 0.01
	dimethylformamide	75 ± 9	0.19 ± 0.03	207 ± 4	0.77 ± 0.03	1345 ± 61	0.04 ± 0.01
	dimethyl sulfoxide	-	0	247 ± 1	0.97 ± 0.01	2333 ± 6	0.03 ± 0.01
	butanol	-	0	328 ± 2	0.97 ± 0.01	3198 ± 15	0.03 ± 0.01
	ethanol	-	0	252 ± 2	0.98 ± 0.01	1720 ± 41	0.02 ± 0.01
	methanol	-	0	279 ± 2	0.90 ± 0.01	2031 ± 6	0.10 ± 0.01
**3c**	toluene	-	0	416 ± 1	0.98 ± 0.01	2360 ± 15	0.02 ± 0.01
	ethyl acetate	32 ± 1	0.39 ± 0.02	229 ± 1	0.59 ± 0.02	2259 ± 17	0.02 ± 0.01
	dichloromethane	22 ± 1	0.28 ± 0.01	371 ± 1	0.71 ± 0.01	≅ 3700	<0.01
	acetone	38 ± 1	0.51 ± 0.01	196 ± 1	0.47 ± 0.01	1553 ± 37	0.02 ± 0.01
	acetonitrile	38 ± 1	0.49 ± 0.01	225 ± 1	0.38 ± 0.01	2017 ± 2	0.12 ± 0.01
	dimethylformamide	-	0	320 ± 1	0.77 ± 0.01	2197 ± 2	0.23 ± 0.01
	dimethyl sulfoxide	-	0	349 ± 1	0.98 ± 0.01	3117 ± 8	0.02 ± 0.01
	butanol	-	0	397 ± 1	0.91 ± 0.01	1270 ± 5	0.09 ± 0.01
	ethanol	-	0	546 ± 2	0.92 ± 0.01	1716 ± 10	0.08 ± 0.01
	methanol	-	0	579 ± 1	0.93 ± 0.01	1943 ± 15	0.07 ± 0.01

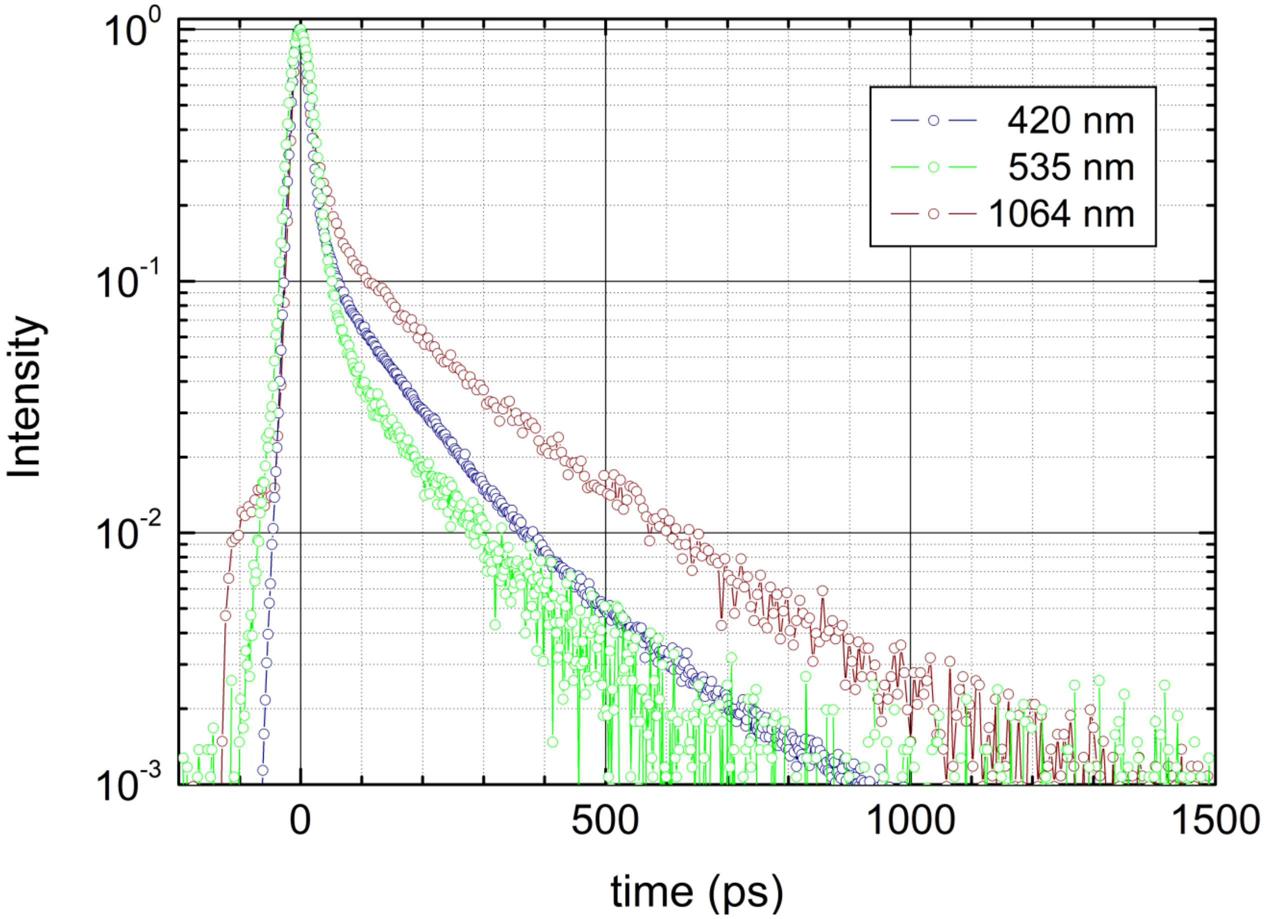

Figure S9. The TCSPC apparatus instrumental response to <10 ps laser pulses at 420 nm (violet, laser pulse duration 2.8 ps), 532 nm (green, laser pulse duration 6.4 ps), and 1064 nm (dark red, laser pulse duration 9 ps). The full-width at half maximum of the temporal point-spread functions is in any case <30 ps.

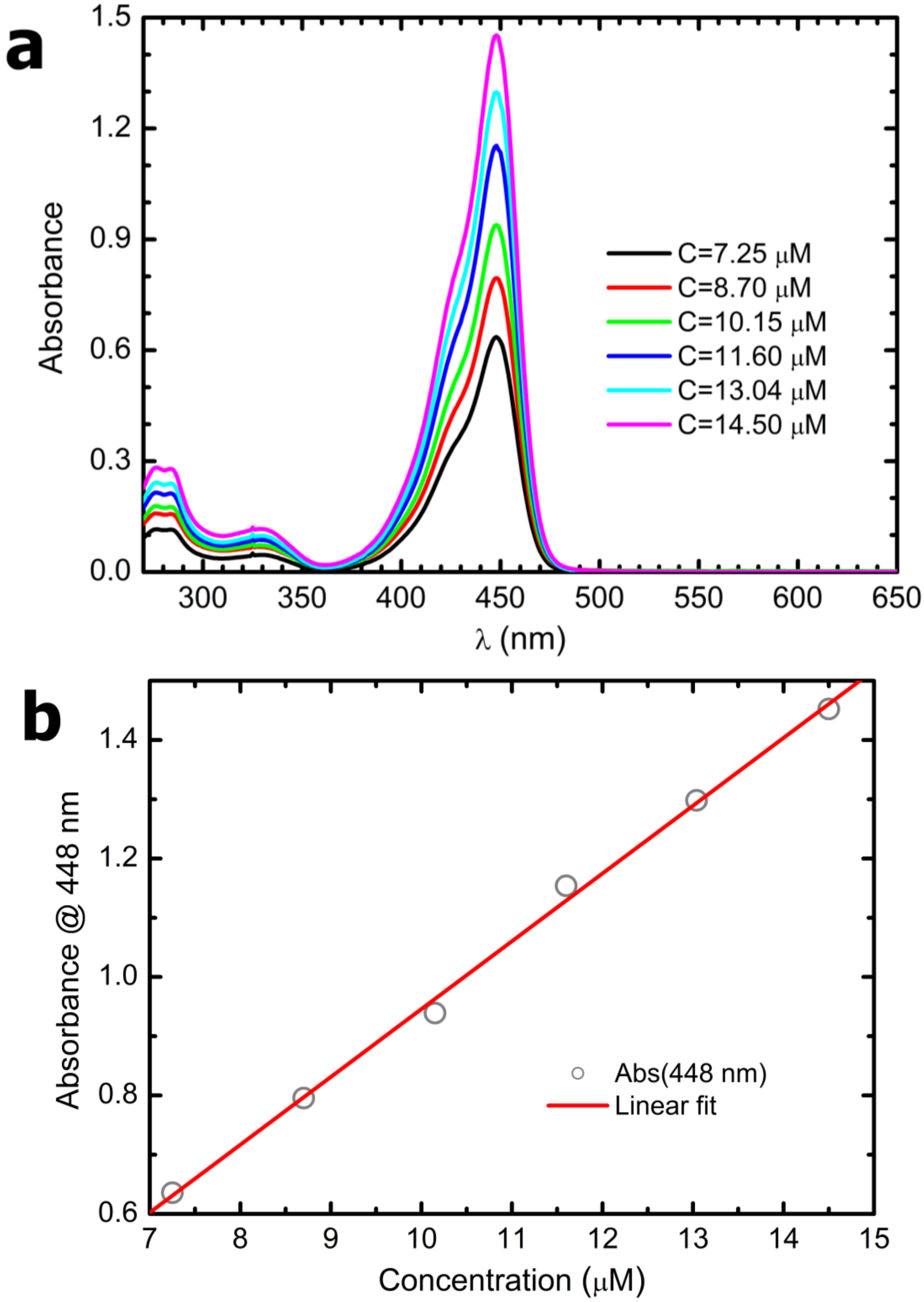

Figure S10. Exemplary dataset for determination of molar extinction coefficient values (namely 4a in dimethyl sulfoxide). a) Absorption spectra recorded for six solutions obtained by dilution of the stock at known concentration (determined by weight, see Materials and Methods); b) peak absorbance versus nominal concentration (dots) and linear regression of the data (red line). The slope is the molar extinction coefficient (in the case at hand 0.1144 $\mu M^{-1}cm^{-1}$).

S9. Supplementary theoretical emission data

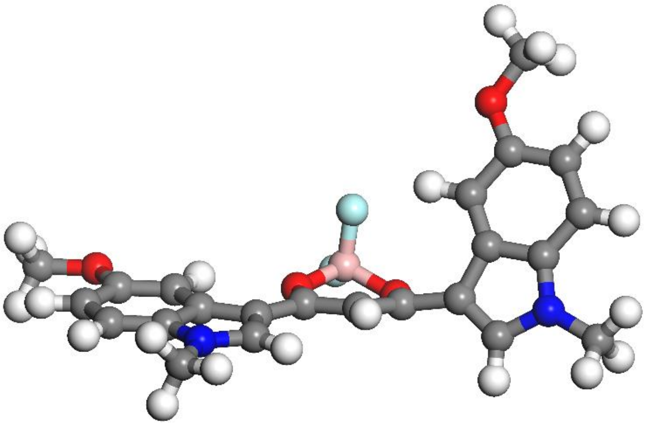

Figure S11. Global minimum geometry for the first excited state of **4c** as obtained at the TD- and TDA-B3LYP-D3/def2-TZVPD. Color code: red (oxygen), pink (boron), blue (nitrogen), light blue (fluoride), grey (carbon), white (hydrogen).

Table S4. Excited state properties of **3a** keto-enol, **3a** trans-diketo, **4a**, **4b**, and **4c** as computed at DFT/TDA-DFT with ωB97X-D/def2-TZVPD. Vertical (ΔE_{vert}), adiabatic (ΔE_{adia}), and ZPVE-corrected adiabatic ($\Delta E_{\mathrm{adiaZPVE}}$) excitation energies for the $S_0 \rightarrow S_1$ transition in the gas phase. The HOMO to LUMO energy difference in S_0 ($\Delta E_{\mathrm{HOMO\text{-}LUMO}}$) and the vertical de-excitation energy for $S_1 \rightarrow S_0$ transition ($\Delta E'_{\mathrm{vert}}$) is also showed for comparison. The energies are defined in Figure 8. All the energies are reported in eV. The absorption wavelength ($\lambda_{\mathrm{abs\omega B97}}$, nm) corresponding to the $S_0 \rightarrow S_1$ transition is also showed with its oscillator strength ($f_{\mathrm{abs\omega B97}}$, a.u.), the dipole moment associated with the transition ($\mu_{S0 \rightarrow S1}$ a.u.), and the radiative decay rates of the S_1 state ($k_{\mathrm{rad\omega B97}}$, s^{-1}), defined as in Equation 4. $k_{\mathrm{rad\omega B97}}$ is computed considering the intensity and the maximum of the corresponding band in the computed spectrum. The emission wavelength ($\lambda_{\mathrm{fluo\omega B97}}$) corresponding to $S_1 \rightarrow S_0$ computed on S_1 geometry and its oscillator strength ($f_{\mathrm{fluo\omega B97}}$) is also reported.

	3a keto-enol	3a trans-diketo [1]	4a	4b	4c
$\Delta E_{\mathrm{HOMO\text{-}LUMO}}$	7.51	8.13	7.15	7.19	7.04
ΔE_{vert}	4.04	4.13	3.76	3.77	3.69
ΔE_{adia}	3.86	3.95	3.65	3.65	3.57
$\Delta E_{\mathrm{adiaZPVE}}$	3.73	3.84	3.54	3.54	3.46
$\Delta E'_{\mathrm{vert}}$	-3.69	-3.68	-3.54	-3.54	-3.46
$\mu_{S0 \rightarrow S1}$	11.4	(0.2, 0.0, 4.1)	13.6	14.4	15.1
$\lambda_{\mathrm{abs\omega B97}}$	306.6	255.1 (300.26, 284.19, 258.67)	329.8	329.0	336.4
$f_{\mathrm{abs\omega B97}}$	1.13	(0.02, 0.00, 0.49)	1.25	1.33	1.36
$k_{\mathrm{rad\omega B97}}$	9.17×10^{9}	2.01×10^{9}	1.04×10^{10}	1.17×10^{10}	1.21×10^{10}
$\lambda_{\mathrm{fluo\omega B97}}$	335.8	263.6 (336.74, 298.3, 266.1)	350.6	350.4	357.8
$f_{\mathrm{fluo\omega B97}}$	1.10	(0.014, 0.01, 0.49)	1.26	1.33	1.37

[1] The lowest energy band in the absorption, emission, and excitation spectra of **3a** *trans*-diketo is the result of the convolution of more than one signal. The wavelength value is the maximum of the band resulting from the convolution of these signals, while in parentheses are reported the three lowest energy transitions contributing to this band. For **3a** trans-diketo, k_{radB3} is computed for the third transition, i.e. the one having the higher oscillator strength.

Table S5. Excited state properties of **3a** keto-enol, **3a** trans-diketo, **4a**, **4b**, and **4c** as computed at DFT/TD-DFT with ωB97X-D/def2-TZVPD. Vertical (ΔE_{vert}), adiabatic (ΔE_{adia}), and ZPVE-corrected adiabatic ($\Delta E_{adiaZPVE}$) excitation energies for the $S_0 \rightarrow S_1$ transition in the gas phase. The HOMO to LUMO energy difference in S_0 ($\Delta E_{HOMO\text{-}LUMO}$) and the vertical de-excitation energy for $S_1 \rightarrow S_0$ transition ($\Delta E'_{vert}$) is also showed for comparison. The energies are defined in Figure 8. All the energies are reported in eV. The absorption wavelength ($\lambda_{abs\omega B97}$, nm) corresponding to the $S_0 \rightarrow S_1$ transition is also showed with its oscillator strength ($f_{abs\omega B97}$, a.u.), the dipole moment associated with the transition ($\mu_{S0\rightarrow S1}$ a.u.), and the radiative decay rates of the S_1 state ($k_{rad\omega B97}$, s^{-1}), defined as in Equation 4. $k_{rad\omega B97}$ is computed considering the intensity and the maximum of the corresponding band in the computed spectrum. The emission wavelength ($\lambda_{fluo\omega B97}$) corresponding to $S_1 \rightarrow S_0$ computed on S_1 geometry and its oscillator strength ($f_{fluo\omega B97}$) is also reported.

	3a keto-enol	3a trans-diketo [1]	4a	4b	4c
$\Delta E_{HOMO\text{-}LUMO}$	7.51	8.13	7.15	7.19	7.04
ΔE_{vert}	3.90	4.10	3.63	3.64	3.57
ΔE_{adia}	3.69	3.92	3.50	3.51	3.44
$\Delta E_{adiaZPVE}$	3.57	3.81	3.40	3.41	3.33
$\Delta E'_{vert}$	-3.49	-3.65	-3.38	-3.38	-3.33
$\mu_{S0\rightarrow S1}$	9.5	(0.2, 0.0, 3.7)	11.4	12.1	13.0
$\lambda_{abs\omega B97}$	317.8	263.5 (302.31, 286.23, 267.52)	342.0	340.7	347.7
$f_{abs\omega B97}$	0.91	(0.02, 0.00, 0.42)	1.01	1.08	1.13
$k_{rad\omega B97}$	5.75 x 10^9	1.44 x 10^9	6.55 x 10^9	7.51 x 10^9	8.08 x 10^9
$\lambda_{fluo\omega B97}$	355.2	272.4 (339.36, 300.46, 277.1, 274.44)	367.2	366.4	372.9
$f_{fluo\omega B97}$	0.84	(0.01, 0.01, 0.15, 0.34)	0.99	1.06	1.12

[1] The lowest energy band in the absorption, emission, and excitation spectra of **3a** *trans*-diketo is the result of the convolution of more than one signal. The wavelength value is the maximum of the band resulting from the convolution of these signals, while in parentheses are reported the three lowest energy transitions contributing to this band. For **3a** trans-diketo, k_{radB3} is computed for the third transition, i.e. the one having the higher oscillator strength.

Table S6. Excited state properties of **3a** keto-enol, **3a** trans-diketo, **4a**, **4b**, and **4c** as computed at DFT/TDA-DFT with M06-2X/def2-TZVPD. Vertical (ΔE_{vert}), adiabatic (ΔE_{adia}), and ZPVE-corrected adiabatic ($\Delta E_{adiaZPVE}$) excitation energies for the $S_0 \rightarrow S_1$ transition in the gas phase. The HOMO to LUMO energy difference in S_0 ($\Delta E_{HOMO\text{-}LUMO}$) and the vertical de-excitation energy for $S_1 \rightarrow S_0$ transition ($\Delta E'_{vert}$) is also showed for comparison. The energies are defined in Figure 8. All the energies are reported in eV. The absorption wavelength (λ_{absM06}, nm) corresponding to the $S_0 \rightarrow S_1$ transition is also showed with its oscillator strength (f_{absM06}, a.u.), the dipole moment associated with the transition ($\mu_{S0\rightarrow S1}$ a.u.), and the radiative decay rates of the S_1 state (k_{radM06}, s^{-1}), defined as in Equation (4). k_{radM06} is computed considering the intensity and the maximum of the corresponding band in the computed spectrum. The emission wavelength ($\lambda_{fluoM06}$) corresponding to $S_1 \rightarrow S_0$ computed on S_1 geometry, and its oscillator strength ($f_{fluoM06}$) is also reported.

	3a keto-enol	3a trans-diketo [1]	4a	4b	4c
$\Delta E_{HOMO\text{-}LUMO}$	5.95	6.57	5.60	5.64	5.51
ΔE_{vert}	3.96	4.05	3.68	3.57	3.60
ΔE_{adia}	3.80	3.90	3.57	3.68	3.49
$\Delta E_{adiaZPVE}$	3.66	3.77	3.46	3.46	3.37
$\Delta E'_{vert}$	-3.64	-3.69	-3.47	-3.58	-3.38
$\mu_{S0\rightarrow S1}$	12.0	(0.2, 0.0, 4.2)	14.3	15.2	15.7
λ_{absM06}	312.9	260.0 (306.45, 292.82, 262.90)	337.3	336.6	344.8
f_{absM06}	1.17	(0.02, 0.00, 0.48)	1.29	1.37	1.38
k_{radM06}	9.54 x 10^9	1.95 x 10^9	1.08 x 10^{10}	1.11 x 10^{10}	1.21 x 10^{10}
$\lambda_{fluoM06}$	340.2	268.4 (336.35, 302.58, 270.81, 268.45)	357.7	357.8	366.5
$f_{fluoM06}$	1.15	(0.011, 0.009, 0.376, 0.1749)	1.31	1.39	1.41

[1] The lowest energy band in the absorption, emission, and excitation spectra of **3a** *trans*-diketo is the result of the convolution of more than one signal. The wavelength value is the maximum of the band resulting from the convolution of these signals, while in parentheses are reported the three lowest energy transitions contributing to this band. For **3a** trans-diketo, k_{radB3} is computed for the third transition, i.e. the one having the higher oscillator strength.

Table S7. Excited state properties of **3a** keto-enol, **3a** trans-diketo, **4a**, **4b**, and **4c** as computed at DFT/TDA-DFT with CAM-B3LYP-D3/def2-TZVPD. Vertical (ΔE_{vert}), adiabatic (ΔE_{adia}), and ZPVE-corrected adiabatic ($\Delta E_{adiaZPVE}$) excitation energies for the $S_0 \rightarrow S_1$ transition in the gas phase. The HOMO to LUMO energy difference in S_0 ($\Delta E_{HOMO\text{-}LUMO}$) and the vertical de-excitation energy for $S_1 \rightarrow S_0$ transition ($\Delta E'_{vert}$) is also showed for comparison. The energies are defined in Figure 8. All the energies are reported in eV. The absorption wavelength (λ_{absCAM}, nm) corresponding to the $S_0 \rightarrow S_1$ transition is also showed with its oscillator strength (f_{absCAM}, a.u.), the dipole moment associated with the transition ($\mu_{S0\rightarrow S1}$ a.u.), and the radiative decay rates of the S_1 state (k_{radCAM}, s^{-1}), defined as in Equation (4). k_{radCAM} is computed considering the intensity and the maximum of the corresponding band in the computed spectrum. The emission wavelength ($\lambda_{fluoCAM}$) corresponding to $S_1 \rightarrow S_0$ computed on S_1 geometry, and its oscillator strength ($f_{fluoCAM}$) is also reported.

	**3a keto-enol**	**3a trans-diketo** [1]	**4a**	**4b**	**4c**
$\Delta E_{HOMO\text{-}LUMO}$	6.32	6.94	5.99	6.03	5.89
ΔE_{vert}	3.96	4.13	3.71	3.72	3.63
ΔE_{adia}	3.81	3.96	3.60	3.61	3.52
$\Delta E_{adiaZPVE}$	3.68	3.82	3.49	3.50	3.41
$\Delta E'_{vert}$	-3.66	-3.69	-3.50	-3.50	-3.42
$\mu_{S0\rightarrow S1}$	11.7	(0.2, 0.0, 4.0)	13.8	14.6	15.2
λ_{absCAM}	312.8	259.3 (299.85, 283.93, 262.58)	334.5	333.7	341.9
f_{absCAM}	1.13	(0.02, 0.00, 0.47)	1.25	1.33	1.35
k_{radCAM}	9.00×10^9	1.66×10^9	1.03×10^{10}	1.16×10^{10}	1.17×10^{10}
$\lambda_{fluoCAM}$	339.2	269.2 (335.65, 297.65, 272.1, 270.22)	354.5	354.3	363.0
$f_{fluoCAM}$	1.10	(0.01, 0.01, 0.28, 0.25)	1.26	1.34	1.37

[1] The lowest energy band in the absorption, emission, and excitation spectra of **3a** *trans*-diketo is the result of the convolution of more than one signal. The wavelength value is the maximum of the band resulting from the convolution of these signals, while in parentheses are reported the three lowest energy transitions contributing to this band. For **3a** trans-diketo, k_{radB3} is computed for the third transition, i.e. the one having the higher oscillator strength.

Table S8. Excited state properties of **3a** keto-enol, **3a** trans-diketo, **4a**, **4b**, and **4c** as computed at DFT/TDA-DFT with B3LYP-D3/def2-TZVPD. Vertical (ΔE_{vert}), adiabatic (ΔE_{adia}), and ZPVE-corrected adiabatic ($\Delta E_{adiaZPVE}$) excitation energies for the $S_0 \rightarrow S_1$ transition in the gas phase. The HOMO to LUMO energy difference in S_0 ($\Delta E_{HOMO\text{-}LUMO}$) and the vertical de-excitation energy for $S_1 \rightarrow S_0$ transition ($\Delta E'_{vert}$) is also showed for comparison. The energies are defined in Figure 8. All the energies are reported in eV. The absorption wavelength ($\lambda_{abs\omega B97}$, nm) corresponding to the $S_0 \rightarrow S_1$ transition is also showed with its oscillator strength ($f_{abs\omega B97}$, a.u.), the dipole moment associated with the transition ($\mu_{S0\rightarrow S1}$ a.u.), and the radiative decay rates of the S_1 state ($k_{rad\omega B97}$, s^{-1}), defined as in Equation 4. $k_{rad\omega B97}$ is computed considering the intensity and the maximum of the corresponding band in the computed spectrum. The emission wavelength ($\lambda_{fluoB3TDA}$) corresponding to $S_1 \rightarrow S_0$ computed on S_1 geometry, and its oscillator strength ($f_{fluoB3TDA}$) is also reported. The S_1 used in the energy and emission spectra calculations have been the CAM-B3LYP-D3 geometries.

	**3a keto-enol**	**3a trans-diketo** [1]	**4a**	**4b**	**4c** [2]
$\Delta E_{HOMO\text{-}LUMO}$	3.84	4.41	3.58	3.61	3.49
ΔE_{vert}	3.55	3.77	3.34	3.32	3.14
ΔE_{adia}	3.49	3.59	3.31	3.30	3.10
$\Delta E_{adiaZPVE}$	3.48	3.57	3.33	3.30	3.13
$\Delta E'_{vert}$	-3.37	-3.34	-3.24	-3.21	-3.03
$\mu_{S0\rightarrow S1}$	11.4	(0.92, 1.45, 0.19)	14.0	14.4	12.7
$\lambda_{absB3TDA}$	349.3	308.8 (329.21, 310.52, 310.49)	371.6	373.1	394.5
$f_{absB3TDA}$	0.99	(0.09, 0.14, 0.02)	1.14	1.17	0.98
$k_{radB3TDA}$	6.18×10^9	4.82×10^7	7.69×10^9	8.05×10^9	5.30×10^9
$\lambda_{fluoB3TDA}$	368.1	314 (371.08,328.8, 320.12)	383.1	385.7	409.8
$f_{fluoB3TDA}$	0.97	(0.03, 0.11, 0.03)	1.19	1.23	1.03

[1] The lowest energy band in the absorption, emission, and excitation spectra of **3a** *trans*-diketo is the result of the convolution of more than one signal. The wavelength value is the maximum of the band resulting from the convolution of these signals, while in parentheses are reported the three lowest energy transitions contributing to this band.

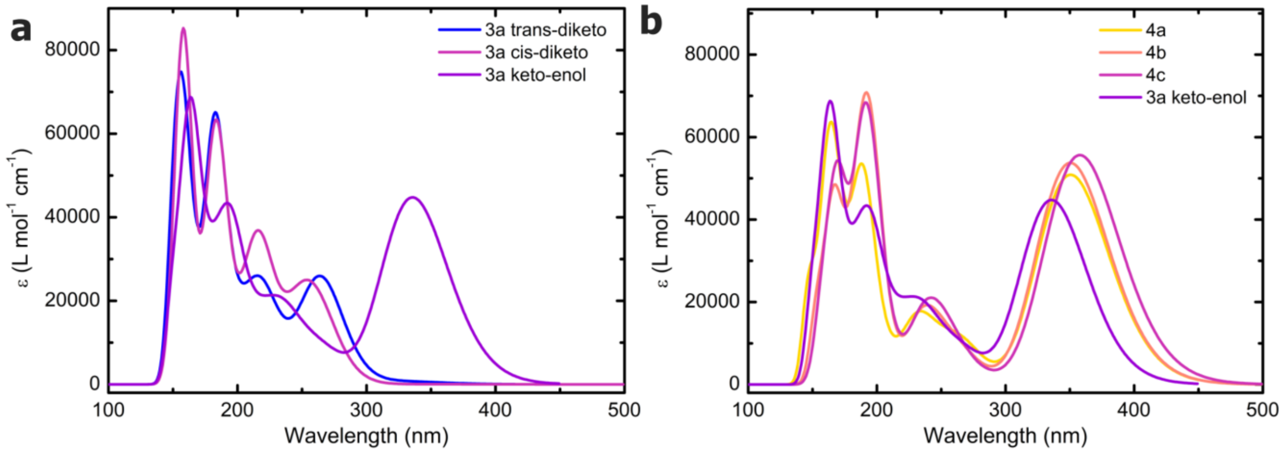

Figure S12. Electronic fluorescence spectra of (**a**) the three conformers of **3a** and (**b**) the BF_2bdks compounds as computed at the TDA-ωB97X-D/def2-TZVPD for S_1 including the first 100 excitations.

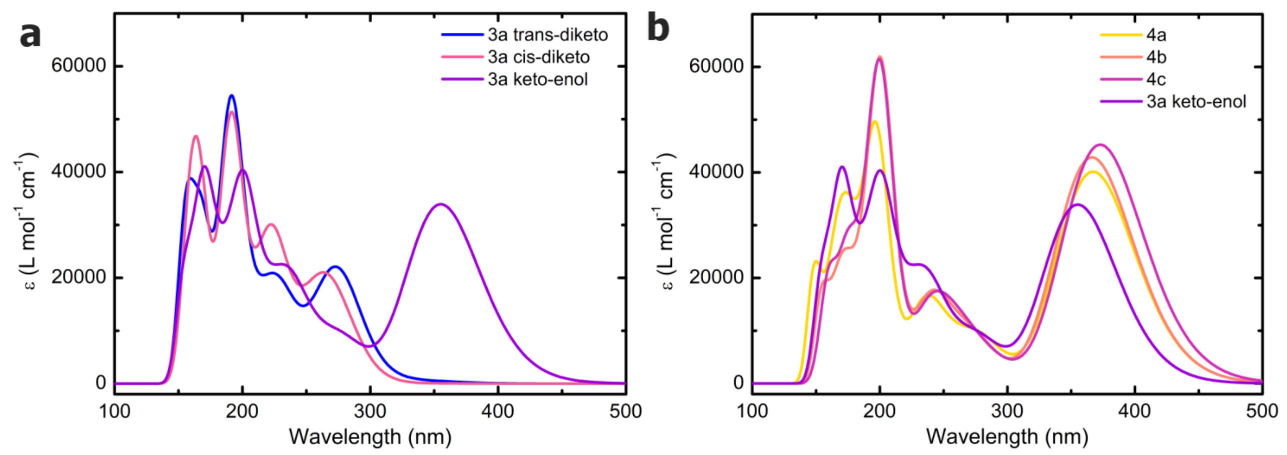

Figure S13. Electronic fluorescence spectra of (**a**) the three conformers of **3a** and (**b**) the BF_2bdks compounds as computed at the TD-ωB97X-D/def2-TZVPD for S_1 including the first 100 excitations.

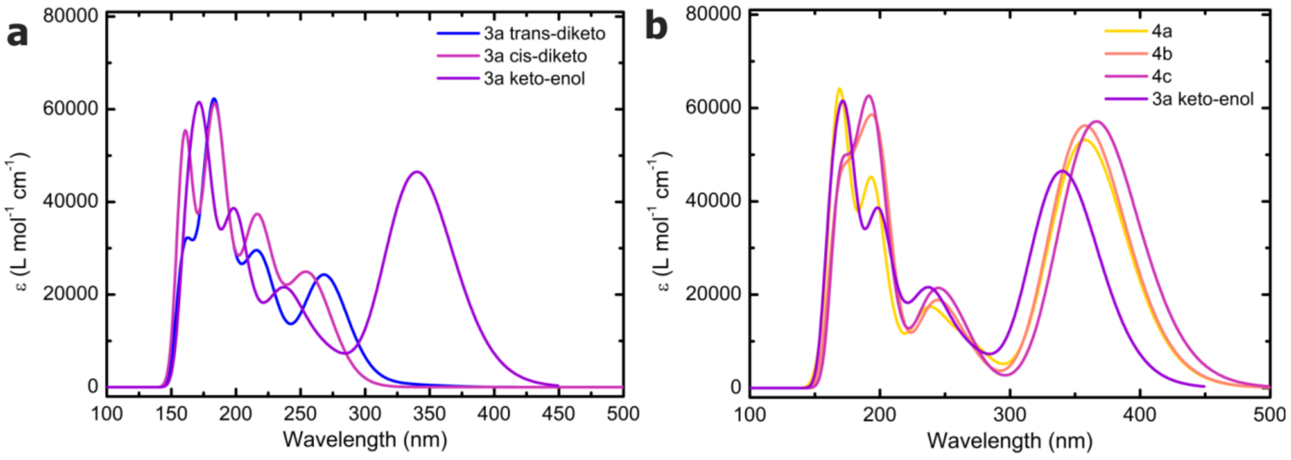

Figure S14. Electronic fluorescence spectra of (**a**) the three conformers of **3a** and (**b**) the BF_2bdks compounds as computed at the TDA-M06-2X/def2-TZVPD for S_1 including the first 100 excitations.

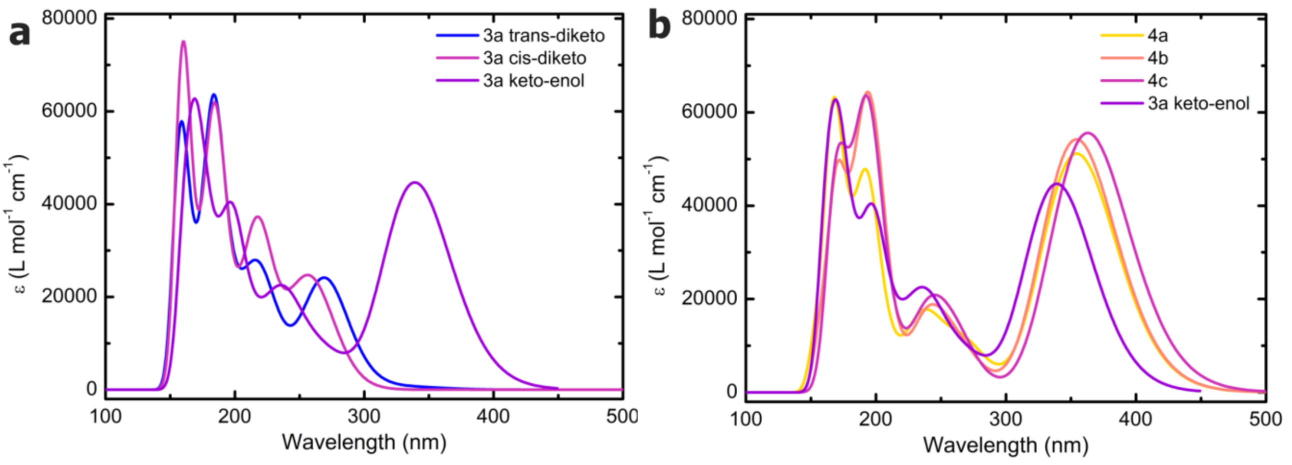

Figure S15. Electronic fluorescence spectra of (**a**) the three conformers of **3a** and (**b**) the BF_2bdks compounds as computed at the TDA-CAM-B3LYP-D3/def2-TZVPD for S_1 including the first 100 excitations.

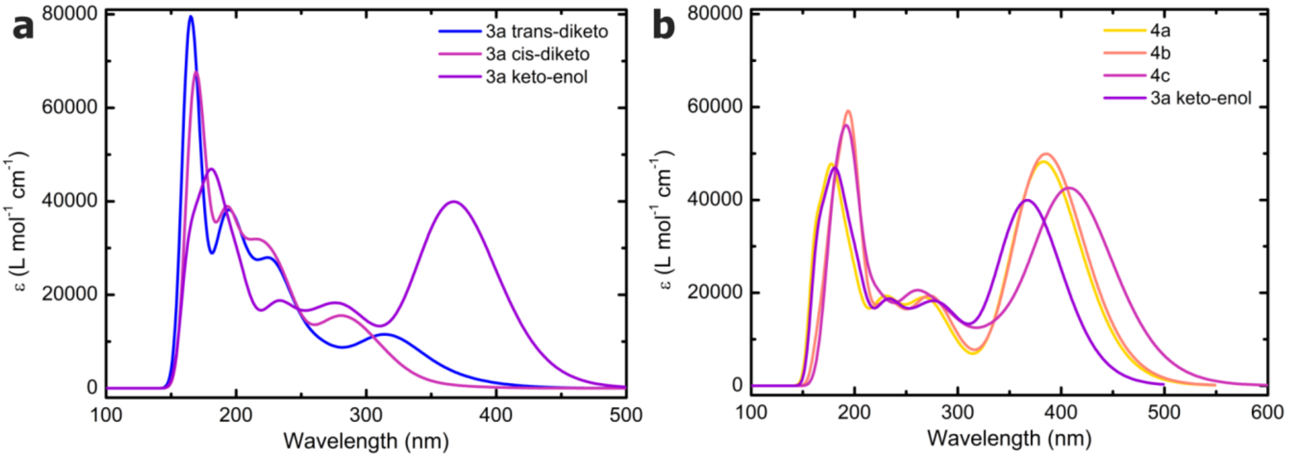

Figure S16. Electronic fluorescence spectra of (**a**) the three conformers of **3a** and (**b**) the BF_2bdks compounds as computed at the TDA-B3LYP-D3/def2-TZVPD for S_1 including the first 100 excitations. The spectra are obtained on the TDA-CAM-B3LYP-D3 geometry for S_1.
